# Efficient Synthesis of Chemically Recyclable Polyamides via Substituent Effects‐Enabled Mechanistic Pathway

**DOI:** 10.1002/anie.202516735

**Published:** 2025-09-14

**Authors:** Youwei Ma, Chihui Zheng, Davide Raphaël Bréas, Gadi Slor, Alain Phillipe Alexandre Molleyres, Qiyue Liao, Francesco Stellacci

**Affiliations:** ^1^ Institute of Materials École Polytechnique Fédérale de Lausanne (EPFL) Lausanne 1015 Switzerland; ^2^ Institute of Bioengineering École Polytechnique Fédérale de Lausanne (EPFL) Lausanne 1015 Switzerland

**Keywords:** Amidation and imination, Chemical recycling, Dimethyl acetone‐1,3‐dicarboxylate, Polyamides, Substituent effects

## Abstract

Imination and amidation are two fundamental condensation reactions central to modern chemical synthesis, and devising energy‐efficient ways to trigger them is highly relevant in advancing low‐carbon manufacturing, with most approaches relying on the use of catalysts. Here, we revisit the dimethyl acetone‐1,3‐dicarboxylate (**DADC**) chemistry, and show that it can react with a broad range of small‐molecule and macromolecular amines at moderate temperatures (80‒120 °C) in the absence of any catalysts. This represents a significant reduction in processing temperatures compared to traditional polycondensation methods for polyamide synthesis, which often require temperatures exceeding 230 °C. Mechanistic and model studies reveal that the high reactivity of **DADC** toward amines arises from its synergistic substituent effects; Specifically, the two ester groups in the symmetric β‐position of **DADC**’s ketone facilitate initial imination via conjugation and electron‐withdrawing effects, generating a β‐enamino intermediate. This β‐enamine subsequently engages in intramolecular hydrogen bonding with one ester group, reducing steric hindrance on the remaining ester and thus promoting its amidation. Moreover, we demonstrate that the **DADC**‐synthesized polyamides are thermally reprocessable, and chemically recyclable under either acidic or basic conditions at mild temperatures, and the chemical recycling is possible both for the neat polymer and its mixture with other plastics.

## Graphical abstract

The joint presence of ketone and diester functionalities in dimethyl acetone‐1,3‐dicarboxylate enhances its reactivity toward amines compared to the individual reactivity of each group, due to substituent effects. This increased reactivity enables the efficient synthesis of polyamides under mild conditions, and the resulting polymers are chemically recyclable.

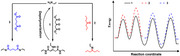

## Introduction

The reactions of amines with aldehydes/ketones (imination) and with carboxylic acids/esters (amidation) are well‐established condensation processes that yield imines and amides, respectively, accompanied by by‐products such as water or alcohols.^[^ ^1^, ^2^, ^3^, ^4^, ^5^ ^]^ These reactions play an indispensable role in organic synthesis, allowing the transformation of amine‐containing compounds into a diverse array of functional materials, with the selected examples including drug intermediates,^[^ ^6^ ^]^ covalent organic frameworks,^[^ ^7^, ^8^, ^9^, ^10^ ^]^ polyamides,^[^ ^11^, ^12^ ^]^ synthetic peptides, and proteins.^[^ ^13^, ^14^ ^]^ Typically, imine formation occurs under acidic conditions and mild heating (60–130 °C),^[^ ^15^, ^16^, ^17^ ^]^ whereas amidation of carboxylic acids or esters requires higher thermal input (> 160 °C), given in the absence of any catalysts.^[^ ^18^, ^19^ ^]^ According to the Le Chatelier's principle, efficient removal of the by‐products is essential to drive both reactions toward completion and ensure high yields and/or conversions.^[^ ^20^ ^]^ As in the industrial production of polyamides through polycondensation pathway, this is often achieved by employing elevated temperatures (above 230 °C) in combination with reduced pressures (∼0.01–10 mbar).^[^ ^11^, ^21^, ^22^, ^23^, ^24^, ^25^ ^]^ Unfortunately, these harsh conditions signify substantial energy consumption and may be incompatible with thermally sensitive or volatile substrates.

Strategies resorting to the use of more reactive species, catalysts, and coupling reagents have been reported to mitigate the above issues.^[^ ^12^, ^25^, ^26^, ^27^, ^28^, ^29^ ^]^ For example, catalysts such as organic phosphites^[^ ^12^ ^]^ and organometallic salts,^[^ ^25^, ^29^ ^]^ as well as the coupling reagents like 1,3‐dicyclohexylcarbodiimide (DCC) in combination with 1‐hydroxybenzotriazole (HOBt) or 4‐(dimethylamino)pyridine (DMAP)^[^ ^27^, ^28^ ^]^ have been employed in the acid‒ or ester‒amine polycondensations, effectively reducing the reaction temperature to a range of 100‒190 °C.^[^ ^25^, ^27^, ^29^ ^]^ The choice of highly reactive acyl chlorides can even lower the polymerization temperature with amines to room temperature level.^[^ ^12^, ^30^, ^31^ ^]^ However, the incorporation of catalysts often requires post‐synthesis removal through additional purification steps to prevent long‐term leaching or product degradation.^[^ ^32^ ^]^ The preparation of acyl chlorides involves hazardous thionyl chloride,^[^ ^33^ ^]^ and also their reaction with amines produces corrosive HCl, raising safety, and environmental concerns. Similarly, in the case of imine formation, various catalysts including metal salts and oxides, molecular sieves, and montmorillonites have been shown to facilitate the reaction at ambient temperatures.^[^ ^15^, ^34^, ^35^, ^36^ ^]^ The underlying principle across these approaches is explicit; they can lower the energy barriers for imination and amidation reactions.

Beyond these strategies, substituent effects—referring to the influence of substituents on the physicochemical and electronic properties of a molecule—have also proven effective in dictating the kinetic and thermodynamic aspects of a reaction, imination and amidation included. Generally, the presence of electron‐withdrawing, less bulky, and/or conjugated substituents adjacent to the carbonyl motif (as in aldehydes, ketones, carboxylic acids, and esters) increases the reactivity of the latter toward amines for the formation of imines and amides,^[^ ^37^, ^38^, ^39^, ^40^, ^41^, ^42^, ^43^, ^44^, ^45^, ^46^, ^47^, ^48^, ^49^ ^]^ so does the alcohol part featuring good leaving groups (such as *tert*‐butyl and aryl moieties) in the case of esters.^[^ ^50^, ^51^, ^52^, ^53^ ^]^ Reported substituents that facilitate these two condensation reactions encompass aryl rings,^[^ ^37^, ^38^, ^39^, ^41^, ^43^, ^45^, ^46^ ^]^ halides,^[^ ^44^ ^]^ phosphates,^[^ ^37^, ^38^, ^40^, ^49^ ^]^ nitro groups,^[^ ^47^ ^]^ sulfonates.^[^ ^38^, ^48^ ^]^ These studies primarily focused on engineering substituent groups to enhance the electrophilicity of the carbonyl carbon, thereby improving the kinetics and conversions of imine and amide formation. However, the reciprocal influence—how the formed imines or amides might affect the properties of the substituent groups—remains largely unexplored, neither does the potential use of these substituents as reactive synthons to further induce reactions. We propose that if a synergistic interplay exists between the carbonyl center and its substituent groups, ideally promoting each other's reactivity, this could pave the way for more efficient syntheses of both small molecules and macromolecules in energy‐efficient and catalyst‐free manners. Such an investigation holds particular relevance in the context of advancing low‐carbon chemical manufacturing processes.

An intriguing example describes the presence of an ester substituent at the β‐position of a ketone in acetoacetates, significantly increasing the reactivity of the β‐ketone toward amines for the formation of β‐enamino esters,^[^ ^54^, ^55^, ^56^ ^]^ inferred from a substantial decrease in the reaction temperature (20 °C versus 80‒130 °C) compared to analogous reactions involving ketones lacking the β‐ester moiety.^[^ ^17^, ^57^, ^58^, ^59^ ^]^ In other words, β‐enamino esters can be synthesized with low energy input. Along with their dynamic nature, the Du Prez team has made a series of progress in applying the β‐enamino esters (known as vinylogous urethanes in their works) to synthesize thermally reprocessable and recyclable thermosets.^[^ ^60^, ^61^, ^62^, ^63^, ^64^, ^65^, ^66^, ^67^, ^68^, ^69^ ^]^

**Scheme 1:**
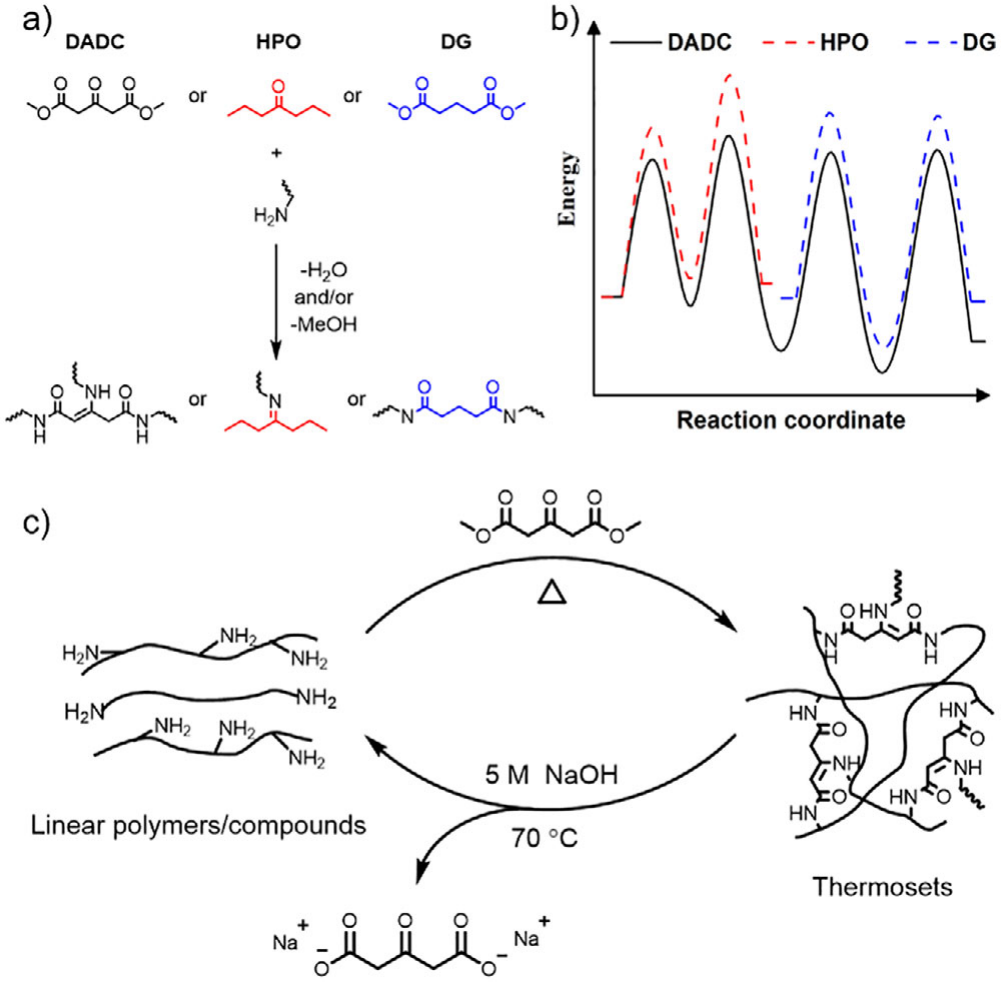
a) Scheme and. b) energy profiles of the reaction of dimethyl acetone‐1,3‐dicarboxylate (**DADC**; black), 4‐heptanone (**HPO**; red), or dimethyl glutarate (**DG**; blue) with an amine compound. c) Illustration of the conversion of amine polymers or compounds into thermosets featuring β‐enaminodiamide (**EADA**) linkages through the reaction with **DADC**, followed by the deconstruction or depolymerization of the thermosets to recover the starting amine materials in the presence of 5 M NaOH.

Motivated by these, here we sought to explore how the β‐enamino group impacts the reactivity of the ester substituent, specifically by selecting dimethyl acetone‐1,3‐dicarboxylate (**DADC**) as the testbed, since it consists of two esters in the symmetric β‐position of a ketone (Scheme 1a). Moreover, the diester functionality provides **DADC** with the potential to synthesize industrially relevant polyamides through polycondensation with bi‐ or multi‐functional amines. Intriguingly, **DADC** exhibits a markedly reduced activation barrier in its reaction with amines, in comparison to its two constituent motifs (i.e., ketone and ester) individually reacting with amines (Schemes 1a,b). This enhanced reactivity stems from synergistic substituent effects rooted in the structure of **DADC**, as elucidated through detailed kinetic and thermodynamic analyses using small‐molecule model studies and density functional theory (DFT) calculations. The chemistry knowledge thus gained translates to the catalyst‐free synthesis of various polyamide thermosets at moderate temperatures (80‒120 °C), ranging from elastomers to plastics, with the amine feedstocks in small‐molecule or macromolecular forms, derived from synthetic or biobased sources (Scheme 1c, top). Moreover, the resulting polyamide thermosets demonstrate good chemical recyclability under either basic conditions at 70 °C (Scheme 1c, bottom) or in the presence of acid, and are also thermally reprocessable.

## Results and Discussion

### Model and Mechanistic Studies on the Reaction of DADC and Amine

**Figure 1:**
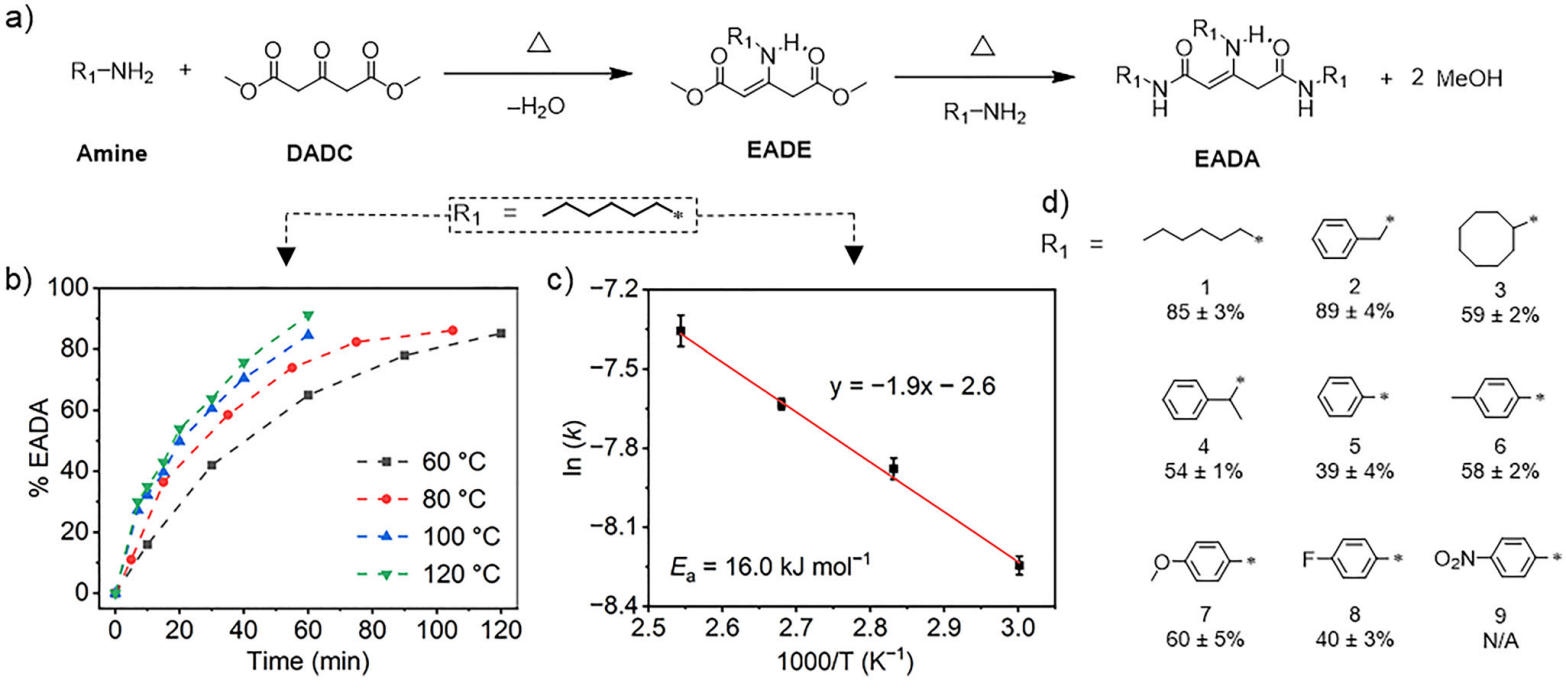
a) The reaction of **DADC** and an amine to form an β‐enaminodiester (**EADE**) prior to the final transformation into an β‐enaminodiamide (**EADA**) derivative. b) Plots showing the extent of conversion of **DADC** into the **EADA** derivative through the reaction with *n*‐hexylamine as a function of time at different temperatures. The dashed lines are guides to the eye. c) Arrhenius plot showing the logarithmic rate constants *k* for the conversion of **DADC** into the **EADA** derivative as a function of inverse temperature. d) Structures of the substituent R_1_ of various amines.

Dimethyl acetone‐1,3‐dicarboxylate (**DADC**) features a central ketone flanked symmetrically by two ester groups at the β‐positions. These two types of functionalities exhibit a reactivity difference toward amines, with the higher electrophilicity present in the ketone.^[^ ^70^ ^]^ Thus, we first investigated the reaction between the ketone and amine (i.e., imination) by mixing **DADC** and 10 eq. of *n*‐hexylamine (**Hea**) in CDCl_3_ at room temperature (Figure 1a, left). ^1^H NMR spectroscopic analysis of the mixture over time allowed to monitor the progress of the reaction (Figure S1). The reaction proceeds steadily and converts 68% of **DADC** into β‐iminodiester (**IDE**) and its tautomer β‐enaminodiester (**EADE**) after 24 h. By contrast, the ^1^H NMR signals of the mixture of 4‐heptanone (**HPO**) and 10 eq. of **Hea** remain barely changed upon the same treatment (Figure S2), suggesting a negligible reaction between them. This phenomenon mirrors the finding mentioned above: the presence of β‐ester can facilitate the reaction between ketone and amine, primarily due to its conjugation with the enol form of the ketone, and the electron‐withdrawing effect.^[^ ^54^, ^55^, ^56^, ^57^, ^58^, ^59^ ^]^

The impact of the formed β‐enamino group on the diester was then explored by reacting **DADC** and excess **Hea** (10 eq.) at higher temperatures including 60, 80, 100, and 120 °C (Figure 1a). The evolution of the reactions was monitored through tracking changes in the ^1^H NMR spectra of the reaction mixture over time (Figure S3). Across all temperatures tested, the signals (1 and 1′) attributed to the methyl groups of **DADC** and **EADE** gradually reduce, while two new signals (6′ and 7′) appear at *δ* = 7.6 and 7.4 ppm, and get intensified upon increasing the reaction time (Figure S3), suggesting the conversion of **DADC** and **EADE** into β‐enaminodiamide (**EADA**) compounds. Integration of the ^1^H NMR signals allowed us to quantify the fraction of the formed **EADA** (Figure 1b). At 60 °C, the extent of conversion for the reaction is 16% after 10 min, and increases substantially to 85% after 2 h. Same conversion is achieved in only 1 h upon increasing the reaction temperature to 100 °C. At 120 °C, the conversion further improves to 91% after 1 h of reaction (Figure 1b). This is in stark contrast to the tiny reaction progress between dimethyl glutarate (**DG**, lacking β‐enamino moiety) and 10 eq. of **Hea** under the same reaction conditions (120 °C, 1 h), as evidenced by no discernible changes in the ^1^H NMR spectra of their mixture (Figure S4). These results demonstrate that the β‐enamino motif can reciprocally promote the diester substituent to react with amines.

The condensation reactions of **DADC** proceeded in the presence of excess **Hea** (Figure S3), thus falling under pseudo first‐order conditions. Establishing the relationship between the extent of conversion of **DADC** and time allowed us to determine the kinetic rate constants (*k*) of the reaction at different temperatures *T* (Figure S5; Table S1). Increasing the reaction temperature from 60 to 120 °C leads to an increase in *k* from 2.63 × 10^‒4^ to 6.38 × 10^‒4^ s^‒1^, reflecting the temperature dependence of the reaction. Further Arrhenius analysis of ln(*k*) versus 1/*T* affords an activation energy *E*_a_ of 16 kJ mol^‒^^1^ (Figure 1c; Table S1).

Further investigations into the reaction kinetics involved varying the **Hea**–**DADC** ratio and either adding solvent or not. By lowering the **Hea** ratio from 10 to 6 and 3 eq., the conversion of **DADC** to **EADA** at 60 °C gradually decreases from 43% to 35% and then to 21% after 30 min of reaction (Figure S6). Moreover, we found that the direct mixture of **DADC** and **Hea** (10 eq.) can also afford **EADA** with a conversion of 74% at room temperature after 24 h. This result is in sharp contrast to the above experiment, carried out under nearly identical conditions except for the presence of CDCl_3_ solvent, which produces only the **EADE** intermediate (Figure S7). All together, these findings suggest that the kinetics of the reaction between **DADC** and **Hea** can be controlled by adjusting their stoichiometric ratio or by employing a solvent. Particularly, the use of a solvent appears to trap the complex reaction at the imination stage.

The presence of β‐enamino functionality differentiates the two esters, depending on their spatial proximity to the adjacent vinyl moiety. This vinyl group can conjugate with the neighboring ester carbonyl, adopting a planar conformation that facilitates intramolecular hydrogen bonding between the enamine and ester functionalities (Figure 1a, middle). In theory, such spatial proximity can induce differentiation in the reactivity of these two esters toward amines. We found that this is indeed the case, and that the conjugated ester exhibits a lower amidation capability. Initial evidence emerged from ^1^H NMR analysis of **DADC** and **Hea** (10 eq.) reactions at 60–120 °C, where the signal 6′ ascribed to the unconjugated amide proton appears earlier and with stronger intensity than that (signal 7′) of its conjugated counterpart (Figure S3). The reactivity of a conjugated ester toward an amine was then examined by the experiment, involving the reaction of methyl acetoacetate (**MAA**) and **Hea** (10 eq.) initially at room temperature for 24 h, and then at 120 °C for 1 h (Figure S8). At room temperature, 94% of **MAA** converts into vinylogous urethane according to ^1^H NMR analysis (Figure S8). This result again shows the enhanced imination capability of the ketone featuring an ester at its β‐position. However, subsequent heating at 120 °C for 1 h fails to produce detectable amounts of β‐enamino amide, as confirmed by both ^1^H NMR and high‐resolution mass spectrometry (HRMS) (Figures S8, S9). This echoes the finding in the works from Du Prez, Winne, and their coworkers,^[^ ^60^, ^64^ ^]^ and strongly suggests the diminished reactivity of the conjugated ester toward amines.

We next investigated the impact of amine substituents (R_1_) on the reactivity toward **DADC** by reacting it with a series of small‐molecule amines (3 eq.) at 120 °C for 12 h (Figure 1a,d). ^1^H NMR and HRMS analyses of the reaction mixtures reveal that alkyl amines such as **Hea** (1) and benzylamine (2) exhibit high conversion of 85% and 89%, respectively, into **EADA** derivatives (Figures S10–S13). In contrast, cyclooctylamine (3) only shows a conversion of 59% (Figures S14, S15), and the introduction of a methyl group at the α‐position of benzylamine (4) reduces the conversion to 54% (Figures S16, S17), highlighting the negative influence of steric hindrance on reactivity. Excluding the steric effect, aryl amines including aniline (5) and its derivatives (6–9) generally exhibit lower reactivity toward **DADC** than alkyl amines, as evidenced by the lower conversions (≤60 ± 5%) after the reaction (Figures S18–S27). This is primarily attributed to the conjugation between the aryl ring and the lone pair electrons of amine in suppressing the nucleophilicity of the latter. Among them (5‒9), the incorporation of electron‐donating methyl and methoxy substituents (6, 7) effectively enhances the reactivity of their aniline derivatives toward amines (Figures S20–S23). For the electron‐withdrawing substitution, fluoro group (8) does not show an obvious impact on the reactivity (Figures S24,S25), as evidenced by the comparable conversion (40% versus 39%) to that of aniline (Figures S18, S19). However, the strong electron‐withdrawing nitro substituent hinders the conversion of 4‐nitroaniline into **EADA**, leading instead to the formation of the **EADE** intermediate at 28% (Figures S26, S27).

**Figure 2:**
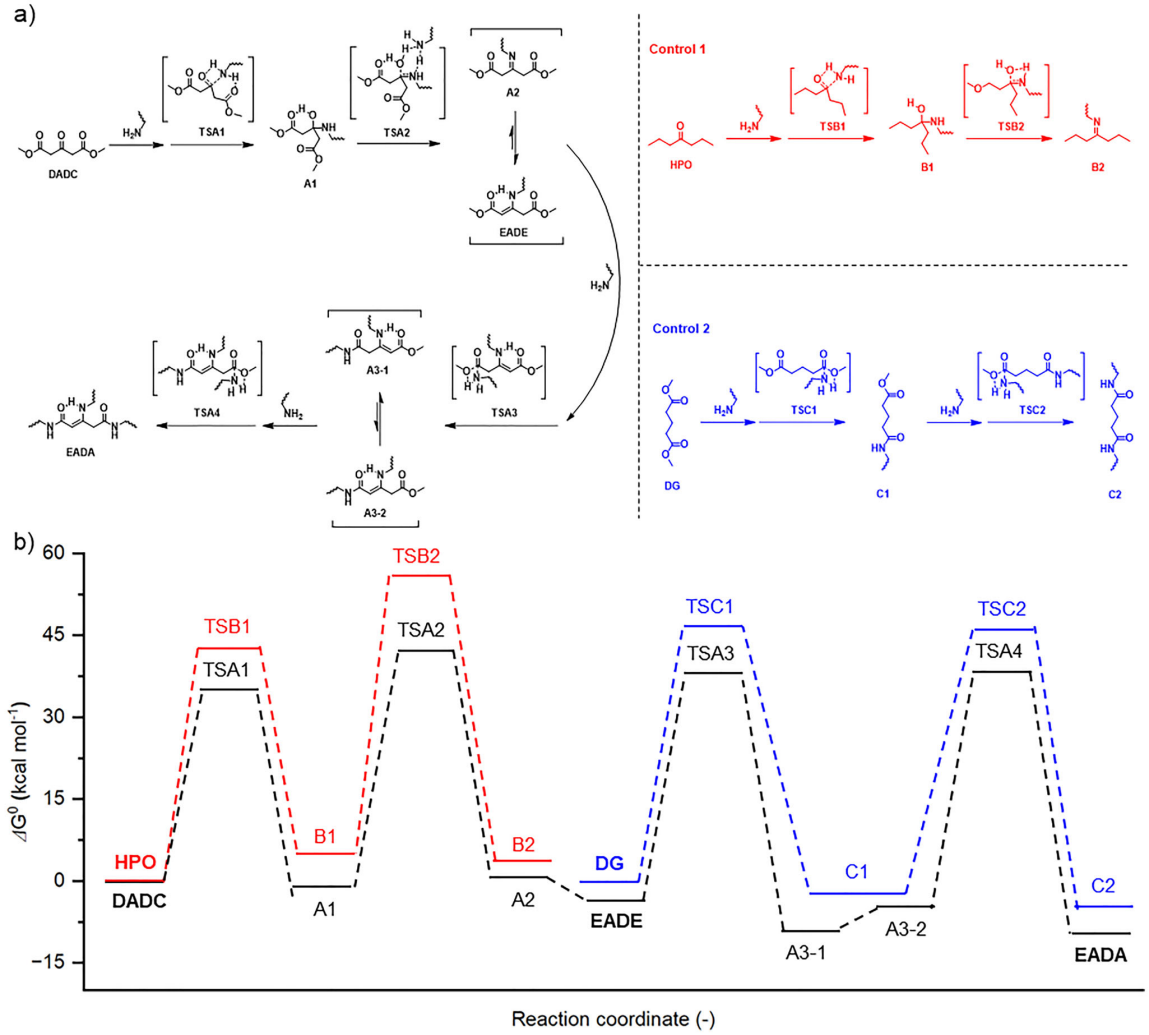
a) Proposed mechanism for the reaction of **DADC** (top), **HPO** (middle), or **DG** (bottom) with **Hea** to afford the corresponding **EADA** derivative, imine **B2**, or diamide **C2**. b) Gibbs free energies (*∆G* ^0^) of the stationary points along the reaction paths in (a), with the reaction between **Hea** and **DADC**, **HPO**, or **DG** highlighted in black, red, or blue color accordingly.

To explore the reaction mechanism, DFT calculations (M06‐2X/6–311++G(d,p) level) were then performed on the reaction of **DADC** and **Hea**, and also on the reactions between **HPO** or **DG** and **Hea** as the control. As shown in Figure 2a, all reactions proceed via an addition‒elimination mechanism. In the **DADC‒Hea** system, the nucleophilic attack of **Hea**’s amino group on **DADC**’s ketone first forms intermediate TSA1, with its two N**‒**H bonds stabilized by the ketone and one ester carbonyl in tetra‐ and hexa‐hedral conformations. The TSA1 exhibits a lower calculated Gibbs free energy (∆*G*_0_) of 35.7 kcal mol^−1^ compared to 42.5 kcal mol^−1^ of the corresponding intermediate TSB1 in the **HPO‒Hea** system (Figure 2a, control 1). This is primarily attributed to the absence of the intramolecular hydrogen bonding‐stabilized six‐membered ring in TSB1. Such the intramolecular hydrogen bond retains until the formation of imine A2 with the calculated ∆*G*_0_ of 1.1 kcal mol^−1^, which soon tautomerizes into more stable **EADE**, showing a ∆*G*_0_ of −3.0 kcal mol^−1^ (Figure 2b). In contrast, the resulting product B2 from the reaction of **HPO** and **Hea** exhibits a positive ∆*G*_0_ as 3.6 kcal mol^−1^, suggesting the thermodynamically unfavorable nature of this reaction. Notably, all intermediate states along the reaction path of **Hea** and **DADC** show lower ∆*G*_0_ values than the corresponding states in the imination of **HPO** (Figure 2b), which explains the faster reaction kinetics and higher reaction conversion in the former two‐involved experiment (Figures S1, S2).

Further attack of **Hea** on the ester moieties of **EADE** could proceed in two potential ways: targeting either the unconjugated or the conjugated ester moiety (Figure S28). DFT calculations analysis shows that the first pathway initially produces intermediate TSA3 with a ∆*G*_0_ of 38.6 kcal mol^−1^ prior to the formation of amide A3‐1, followed by the tautomerization of the A3‐1 into A3‐2 to deconjugate the unreacted ester (Figures S28a, S1). The subsequent reaction of **Hea** with this ester affords the intermediate TSA4 of ∆*G*_0_ of 38.2 kcal mol^−1^ before the final formation of **EADA** (Figures S28a, S1). In the pathway involving the attack on conjugated ester (Figures S28a, S2), the ∆*G*_0_ values of intermediates TSA3i and TSA4i are 49.4 and 45.8 kcal mol^−1^, respectively, both higher than those of TSA3 and TSA4 (Figure S28b). The higher ∆*G*_0_ strongly supports that **Hea** does not prefer to attack on the conjugated ester, which we attribute to the presence of electron‐donating amino group in lowering the electrophilicity of its ester carbon through resonance. This provides a theoretical explanation for the above experimental observations: 1) the earlier emergence of signal 6′ corresponding to the unconjugated amide proton in the ^1^H NMR spectra of the reaction mixture of **DADC** and **Hea** at high temperatures (Figure S3); 2) no formation of β‐enamino amide upon reacting **MAA** and **Hea** at 120 °C (Figures S8, S9).

Having established the optimized reaction pathway, a comparison with the reaction between **DG** and **Hea** was made (Figure 2a, control 2), to further elucidate the impact of the β‐enamino group on diester reactivity. Notably, the key intermediates TSA3 and TSA4 present in the reaction of **Hea** and **EADE** show lower ∆*G*_0_ than their corresponding counterparts TSC1 and TSC2 in the **DG**‒**Hea** reaction (Figure 2b). This energetic advantage is probably attributed to the presence of intramolecular hydrogen bonding in reducing conformational flexibility—thus furnishing less steric hinderance—, together with the weak electron‐withdrawing character of the β‐enamino motif (Figure 2a, left), which facilitates nucleophilic attack on the unconjugated ester. Following methanol elimination, TSA3 converts to monosubstituted amide A3‐1, and it can undergo tautomerization readily to form A3‐2, indicated by a low *E*_a_ of 4.0 kcal mol^−1^ (Figure 2b). This tautomerization step, which deconjugates the remaining ester, is a prerequisite in sustaining high momentum for the subsequent amidation. Moreover, the final product **EADA** exhibits a ∆*G*_0_ of −9.5 kcal mol^−1^, which is also lower than −4.9 kcal mol^−1^ of C2, formed from the reaction of **DG** and **Hea**. Collectively, these results reflect that the reaction of **Hea** with **EADE** exhibits enhanced kinetic and thermodynamic favorabilities relative to the **DG**‒**Hea** reaction, which theoretically justifies the higher reaction kinetics and conversion present in the former reaction (Figures S3, S4).

### Synthesis, Characterization, and Mechanical Properties of the EADA‐Containing Thermosets

**Figure 3:**
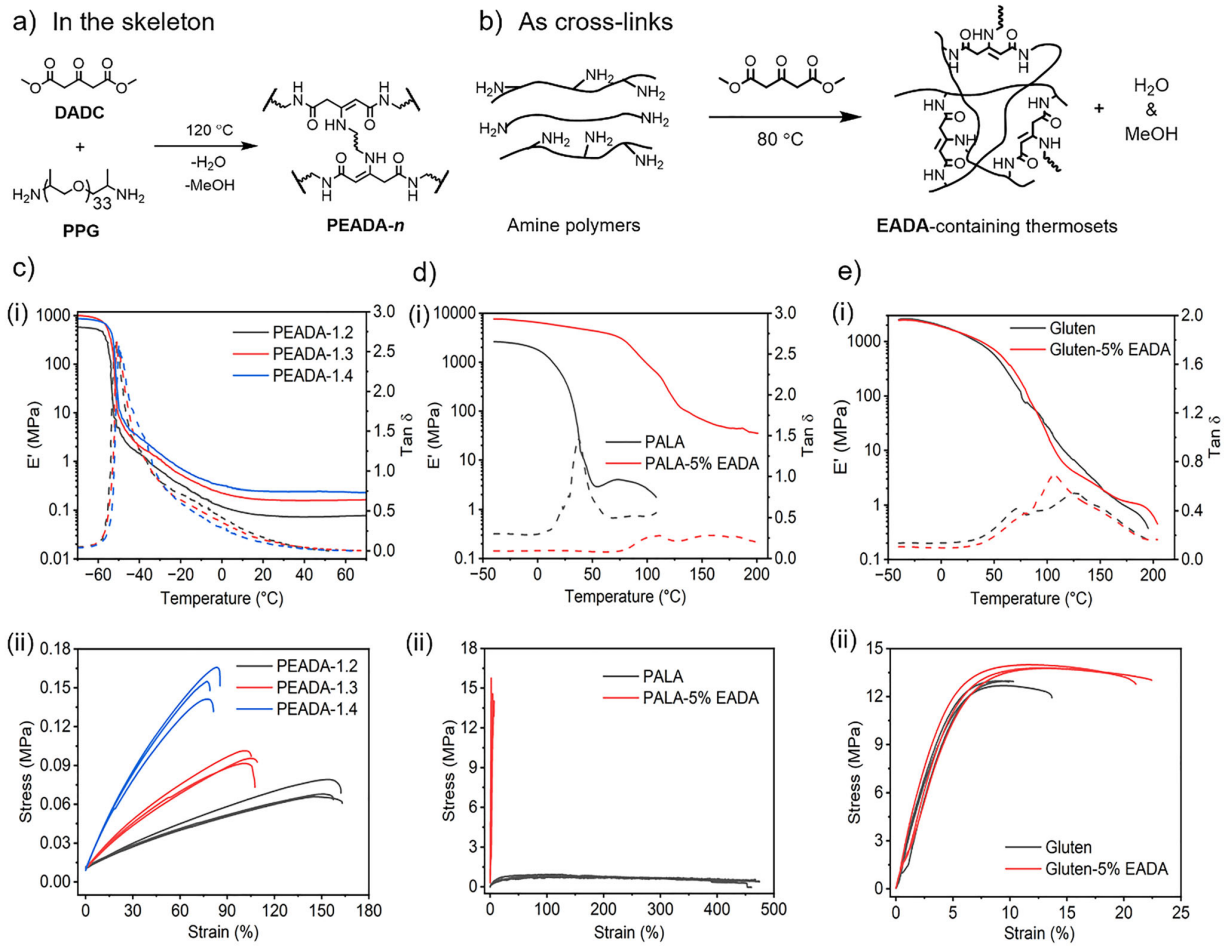
Synthesis of. a) poly(β‐enaminodiamide)s **(PEADA‐*n***) from the polymerization of **PPG** and **DADC** at 120 °C, and. b) other **EADA**‐containing thermosets through the reaction of **DADC** with polyallylamine (**PALA**) or gluten upon thermal treatment at 80 °C to form **PALA‐5%EADA** or **Gluten‐5%EADA**, respectively. (Thermo)mechanical analysis of. c) the **PEADA‐*n*** series, d) **PALA** and **PALA‐5%EADA**, e) gluten and **Gluten‐5%EADA**, with the DMA traces (*E*’ in solid lines and tan *δ* in dashed lines) and stress‒strain curves shown in (i) and (ii), respectively.

Based on the chemical framework established above, we next synthesized some representative thermosets comprising the **EADA** linkages either in the skeleton or as cross‐links, through reacting **DADC** and small‐molecule or macromolecular, synthetic or bio‐based amines at moderate temperatures (80‒120 °C) in the absence of any catalyst (Figure 3a,b). Engineering **EADA** bonds in the skeleton of the thermosets was systematically investigated by polymerizing **DADC** and various amounts of poly(propylene glycol) bis(2‐aminopropyl ether) (**PPG**) at 120 °C, which produces poly(β‐enaminodiamide)s **(PEADA‐*n***), with parameter ***n*** representing the molar feeding ratio of the reactive functional groups of **DADC** (i.e., ketone and ester) to **PPG** (i.e., amine) as 1.2, 1.3, or 1.4 (Figure 3a; synthesis recipes seen in Table S2). The starting materials **PPG**, **DADC**, and the as‐synthesized **PEADA‐*n*** were analyzed by Fourier transform infrared (FTIR) spectroscopy. It displays the disappearance of the ketone and ester carbonyl peaks of **DADC** at 1716 and 1737 cm^‒1^, respectively, and the appearance of three new vibrational bands at 1740, 1710, and 1670 cm^‒1^ in **PEADA‐1.2** (Figure S29b), which indicate the transformation of **DADC** to **EADA** bonds. Moreover, the intensities of these new bands increase with the value of ***n*** (Figure S29c), suggesting more **EADA** linkages formed.

Given the divergent reactivity of the ketone and ester moieties of **DADC** toward amines, **PEADA** can be synthesized in a two‐step synthetic approach through precise temperature control. In the first step, **PPG** and 0.8 eq. of **DADC** were mixed and reacted at room temperature for 1 week, and the mixture solution gradually developed a yellow coloration while remaining in the liquid phase (Figure S30a). Analyses of the solution by ^1^H NMR and FTIR spectroscopies show that two signals at *δ* = 8.5 and 4.4 ppm appear in the ^1^H NMR spectrum (Figure S30b), and the band at 1737 cm^‒1^ (ascribed to the stretching vibration of ester carbonyl) preserves and a new band at 1660 cm^‒1^ emerges in the FTIR spectrum (Figure S30c). These reflect the selective formation of the enamine intermediate from the reaction of the β‐ketone of **DADC** and the amino groups of **PPG**. Further thermal treatment at 120 °C triggered the amidation of the ester groups, yielding a brownish **PEADA‐1.2** film, as confirmed by FTIR analysis (Figure S30c). This sequential strategy effectively exploits the substantial kinetic disparity between the two reactions, enabling the formation of a stable, single‐component liquid precursor at ambient temperature. The resulting precursor not only exhibits excellent flowability but also can undergo thermal curing at 120 °C, characteristics that are particularly advantageous for applications such as coatings.

The chemical resistance of **PEADA‐*n*** networks was evaluated by immersing **PEADA‐1.2** films in neutral, basic, and acidic H_2_O, and various organic solvents (including acetone, tetrahydrofuran, toluene, acetonitrile, ethanol, and DMSO) at room temperature for 24 h (Figure S31). The material exhibited only swelling without dissolution in any of the cases investigated, suggesting the existence of the cross‐linking structure and robust resistance to these chemicals. We then measured the swelling ratio and gel fraction of the **PEADA‐*n*** films by putting them in DCM for 24 h (Figure S32a). As the value ***n*** increases from 1.2 to 1.4, the swelling ratio decreases from 1740% to 1320%, while the gel fraction slightly increases from 76% to 80% (Figure S32b), indicative of the increased cross‐link density.

The thermal properties of the **PEADA‐*n*** films were analyzed by thermal gravimetric analysis (TGA) and differential scanning calorimetry (DSC). The TGA curves show that all **PEADA‐*n*** films exhibit an almost identical thermal decomposition profile, with 5% weight loss occurring at ca. 350 °C (Figure S33a), demonstrating their excellent thermal stability. DSC traces reveal that the increase of value ***n*** leads to an increase in glass transition temperature (*T*_g_), from −58.2 °C of **PEADA‐1.2** to −57.3 °C of **PEADA‐1.4** (Figure S33b), which primarily results from the more restricted chain mobility imposed by the increased number of cross‐links.^[^ ^71^ ^]^

The thermomechanical properties of the **PEADA‐*n*** samples were studied by dynamic mechanical analysis (DMA) (Figure 3c,i). All samples exhibit a discernible drop in storage modulus (*E*′) at the temperature between −51.3 and −49.3 °C, correlating with a peak present in the tan *δ* plots, which is attributed to their *T*_g_ (Figure 3c,i). Following the transition, the materials enter a rubbery plateau, with *E*’ values ranging from 0.07 to 0.24 MPa, reconfirming the presence of a cross‐link architecture within them (Figure 3c,i). Specifically, the *E*′ value of the rubbery regime increases with the value of ***n***, suggesting a progressive rise in cross‐link density, consistent with the result seen in the swelling experiment (Figure S32). The increase of cross‐link density slightly shifts the tan *δ* peaks of **PEADA‐*n*** toward higher temperatures, demonstrating an increase in their *T*_g_, which is in good agreement with the finding in the DSC analysis (Figure S33b).

The mechanical properties of **PEADA‐*n*** were initially tested by uniaxial tensile testing (Figure 3c,ii; Table S3). The resulting stress–strain curves display the typical behavior of elastomeric polymers, with the stress at break of 0.08–0.15 MPa and the strain at break of 78%–160% (Figure 3c,ii). Increasing the number of cross‐links (i.e., the value ***n***) leads to a continuous increase in stress at break while a decrease in strain at break (Figure 3c,ii; Table S3). The elasticity of the polymer networks was investigated by subjecting **PEADA‐1.4** to cyclic tensile testing, which involves repeatedly stretching and releasing the film to a maximum strain of 50% for 20 consecutive cycles (Figure S34). The **PEADA‐1.4** exhibits a substantial hysteresis loop in the first cycle, followed by the narrowing of the loop from the second cycle onward. Concurrently, a gradual increase in residual strain is observed, which stabilizes at ca. 7% (Figure S34), indicating good elasticity of this elastomer.

By changing the amine feedstock from **PPG** to crystallizable 1,12‐dodecanediamine (**DDA**) in the polymerization with **DADC** at 120 °C, a poly(1,12‐dodecanediamide) **(PDDA**) film was accessed (Figure S35a). The presence of a sharp peak at 1660 cm^‒1^ and a shoulder peak at 1743 cm^‒1^ in the FTIR spectrum of **PDDA**, ascribed to the stretching vibrations of amide carbonyl and vinyl motifs, respectively, reveals the successful formation of **EADA** linkages in the polymer (Figure S35b). The **PDDA** networks exhibit a swelling ratio of 310% and a gel fraction of 86% (Figure S35c). Further analyses on the film by DMA and tensile testing measurements disclose that it exhibits a melting point of 70 °C (Figure S35d), and a plastic‐like tensile behavior, with stress and strain at break of 6.0 MPa and 50%, respectively (Figure S35e).

The versatility of the **DADC** chemistry was further explored by its application in cross‐linking synthetic or naturally occurring amine polymers, enabling a transformation from linear architectures to cross‐linked networks. Such topological modifications are known to enhance or tailor material properties.^[^ ^72^, ^73^, ^74^ ^]^ Apart from this, one key advantage of this chemistry consists in its high reactivity, enabling efficient amidation at relatively mild temperatures as low as 60 °C (Figure 1b). This feature is particularly beneficial for modifying thermally sensitive amine polymers, with the examples of polyallylamine (**PALA**),^[^ ^75^, ^76^ ^]^ chitosan,^[^ ^77^, ^78^ ^]^ proteins.^[^ ^79^, ^80^ ^]^

To verify this, the first example that we investigated here involved cross‐linking **PALA**, which was obtained through the deprotonation of polyallylamine hydrochloride (**PALA•HCl**) by NaOH. **DADC** was introduced in 5 mol% relative to the repeat units of **PALA**, and the mixture was heated at 80 °C to yield **EADA**‐cross‐linked **PALA** (termed as **PALA‐5%EADA**; synthesis scheme in Figure S37a). As shown in Figures S36 and S37b, ^1^H NMR and FTIR spectroscopic analyses confirm the successful deprotonation and subsequent cross‐linking reaction between **PALA** and **DADC**. The resulting **PALA‐5%EADA** shows a high gel fraction of 95% (Figure S37c). It was then subjected to DMA measurement, with the result compared with that of **PALA** (Figure 3d,i). **PALA‐5%EADA** exhibits a thermal transition at 110 °C, followed by a stable rubbery plateau in *E*’ extending up to 200 °C; on the contrary, a sharp decline in *E*’ for **PALA** happens at 36 °C prior to the melting down of the polymer at 108 °C (Figure 3d,i). This stark difference underscores the effectiveness of the cross‐linking in enhancing the thermomechanical properties of **PALA**. Moreover, analysis of these two materials by tensile testing reveals that upon cross‐linking, **PALA**, and **PALA‐5%EADA** display strikingly different tensile behaviors; the linear polymer is soft and extensible, whereas its cross‐linked counterpart becomes rigid and strong (Figure 3d,ii).

The second case is to transform biobased proteins, rich in amino groups on the side chains, into plastic films, while improving the mechanical properties of resulting plastics through the cross‐linking with **DADC**. Given the growing environmental concerns associated with synthetic, (mostly) non‐recyclable plastics, the efficient valorization of renewable proteins into bioplastics holds huge promises and has recently attracted significant attention.^[^ ^81^, ^82^, ^83^ ^]^ Here, we selected gluten as the representative to prepare a bioplastic, which involves initial kneading of the mixture of gluten, 30 wt% glycerol (as the plasticizer), and 5 wt% **DADC** into a dough, followed by compression‐molding the dough at 80 °C under 40 kN pressure for 20 min (Figure S38a). The resulting bioplastic, termed as **Gluten‐5%EADA**, was compared to a control film prepared under identical conditions but without **DADC**. FTIR spectra reveal that **Gluten‐5%EADA** exhibits enhanced intensities at 1743 and 1663 cm^‒1^ than the control gluten film (Figure S38b), suggesting the formation of **EADA** cross‐links. It exhibits both low swelling ratio (180%) and gel fraction (65%) (Figure S38c), with latter attributed to the dissolution of glycerol in the testing solvent (i.e., water). The cross‐link architecture endows the **Gluten‐5%EADA** film with slightly enhanced (thermo)mechanical properties, as demonstrated by the increases of *T*_g_, stress and strain at break to 108 °C, 13.3 MPa, and 22% from 73 °C, 12.3 MPa, and 12% of the uncross‐linked film in the DMA and tensile testing analyses (Figure 3e; Table S3).

### Chemical Recycling of EADA‐Containing Thermosets

Having demonstrated the feasibility of the **DADC** chemistry in the efficient synthesis of a wide range of amide‐containing thermosets—thereby addressing several challenges associated with their production and use—, we last endeavored to explore the chemical recyclability of the **EADA**‐cross‐linked networks. Such investigation is highly relevant in the pursuit of a circular materials economy, yet it is considerably more challenging than recycling thermoplastics because of the permanent cross‐link architecture present in thermosets.^[^ ^74^, ^84^ ^]^ As outlined earlier, **EADA** bonds form via two sequential condensation reactions: imination followed by amidation. While condensation reactions are, in principle, reversible—with the reaction equilibrium theoretically tunable by the addition and removal of by‐products—,^[^ ^85^, ^86^ ^]^ the amide bond is well known for its resonance‐stabilized structure, which confers high kinetic and thermal stability. Consequently, depolymerization of conventional polyamides typically requires harsh conditions, including elevated temperatures (often exceeding 250 °C) and, in some cases, increased pressure. ^[^ ^20^, ^87^ ^]^ Encouragingly, such energy‐intensive treatments are not necessary in the chemical recycling of our **EADA**‐containing thermosets.

We started the investigation by synthesizing a trihexyl‐substituted **EADA** model compound through the reaction of **DADC** and an excessive amount of **Hea** at 120 °C for 24 h. The successful synthesis of the **EADA** compound was confirmed by ^1^H NMR, ^13^C NMR spectroscopies and HRMS (Figures S39–S41). The hydrolysis of the **EADA** was carried out in the mixture solvent of methanol‐*d*_4_ and 5 M NaOH (2/1, *v/v*) at 70 °C, and the progress of the reaction was estimated via ^1^H NMR spectroscopy (Figure S42). The ^1^H NMR spectra show that the hydrolysis of the model compound proceeds steadily and generates **Hea** again. Integration of the ^1^H NMR signals exhibits a hydrolysis conversion of 6.4% after 1 h, 28.3% after 24 h, and 49.3% after 5 days. Clearly, the hydrolysis process is far from appealing under these conditions. By replacing methanol‐*d*_4_ into ethanol‐*d*_6_ in the mixture with 5 M NaOH and maintaining all other parameters constant, a large difference was noted (Figure S43); Conversion reaches 18.7% after 1 h and 91.2% after 3 days (Figure S43), compared to only 36.2% in the methanol system at the same time point (Figure S42). This acceleration is attributed to the improved solubility of the hydrophobic trihexyl‐substituted **EADA** in the ethanol‐d_6_/NaOH medium, which facilitates more effective interaction with hydroxide ions.

The high hydrolysis efficiency, achieved at the considerably low temperature (i.e., 70 °C), encouraged us to further elucidate the underlying mechanism. DFT calculations (M06‐2X/6–311++G(d,p) level) were then conducted on the base‐catalyzed hydrolysis of the trihexyl‐substituted **EADA** (Figure S44). According to the above ^1^H NMR analysis (Figures S42, S43), the signal *d*_2_ ascribed to the proton of the conjugated amide disappears first, followed by the concurrent disappearance of signals *d*_1_ and *e* corresponding to the unconjugated amide and imine, respectively. We therefore hypothesized the hydrolysis of the **EADA** compound to proceed in the pathway, consisting of an initial attack of a hydroxide on the carbonyl of the conjugated amide in eliminating a **Hea** molecule, and the subsequent attacks on the unconjugated amide and imine functionalities to generate two additional **Hea** molecules (Figure S44a). DFT calculations on the process show that, the calculated *E*_a_ is 35.0 kcal mol^−1^ (Figure S44b), which is lower than that (42.4 kcal mol^−1^) for the hydrolysis of a conventional amide, *N*‐hexylhexanamide (Figure S45). Such the discrepancy in *E*_a_ underpins the higher propensity of the **EADA** to hydrolyze, which we attribute to the presence of intramolecular hydrogen bonding within the β‐enamino ester of **EADA** that helps to stabilize the intermediate states along the hydrolysis pathway (Figure S44).

**Figure 4:**
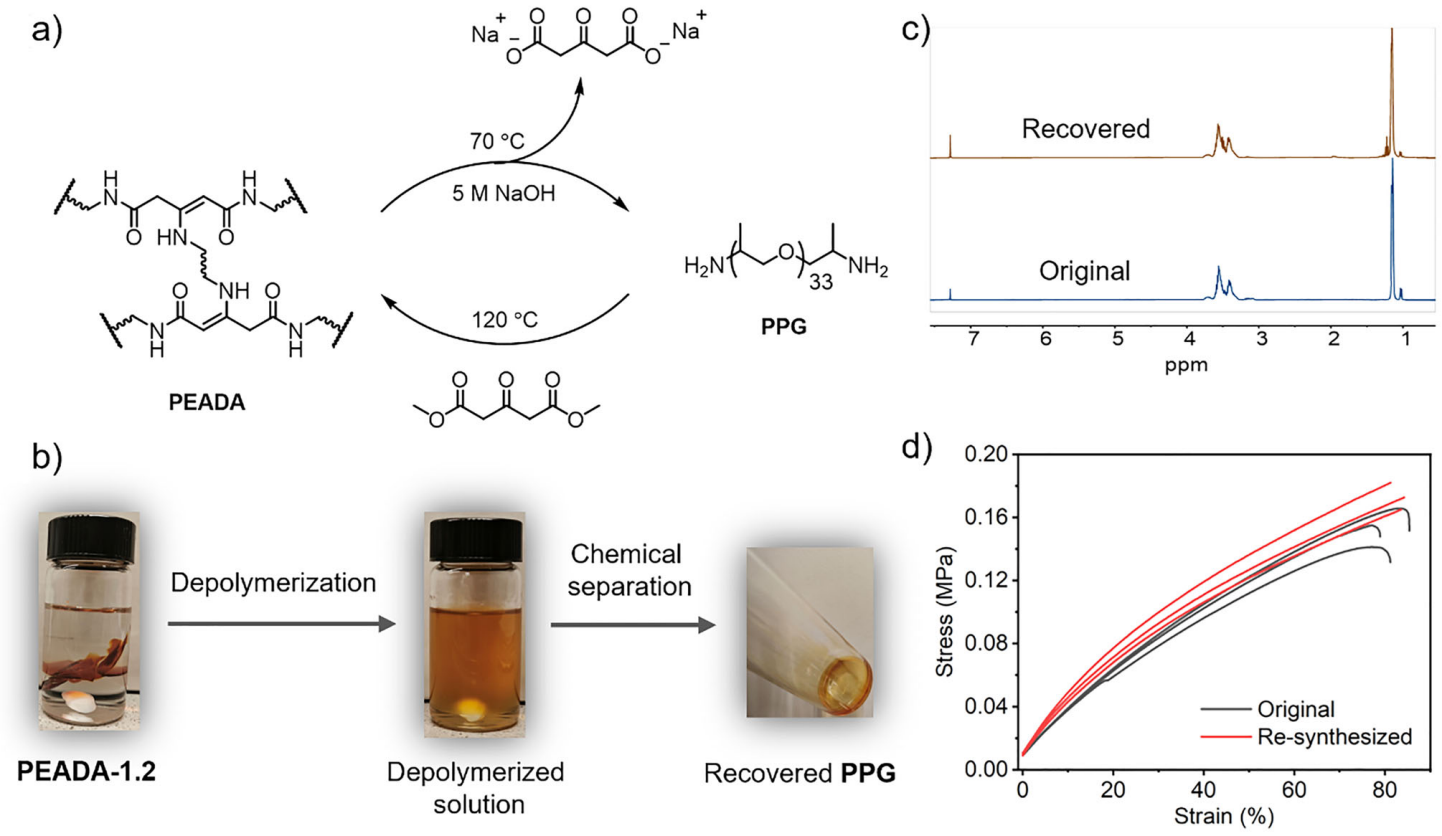
a) Depolymerization of **PEADA‐1.2** by 5 M NaOH at 70 °C and chemical separation of the depolymerized product to recover **PPG**, followed by the polymerization of the recovered **PPG** and 0.93 eq. of **DADC** to produce **PEADA‐1.4**. b) Photographs showing the depolymerization of **PEADA‐1.2** (left) by an ethanol‒5 M NaOH mixture solution (2:1, *v/v*) upon thermal treatment at 70 °C, and the subsequent separation treatment on the depolymerization solution (middle) to recover **PPG** (right). c) Comparison between the ^1^H NMR spectra (CDCl_3_, 400 MHz) of the original and recovered **PPG** from the depolymerization of **PEADA‐1.2** shown in (b). d) Stress–strain curves of the original (black) and re‐synthesized (red) **PEADA‐1.4**.

With this chemistry knowledge, we next set out to depolymerize **EADA**‐containing thermosets through the base‐catalyzed hydrolysis (Figure 4a, top). **PEADA‐1.2** was chosen as the representative, as its **EADA** motifs are embedded in the polymer skeleton—unlike **PALA‐5%EADA** and **Gluten‐5%EADA**, where they serve solely as cross‐links—, thereby presenting a greater depolymerization challenge. Specifically, 0.5 g of **PEADA‐1.2** was immersed in a mixture solution (30‐fold by weight) comprising ethanol and 5 M NaOH (2:1, *v/v*) and heated at 70 °C (Figure 4b). The polymer initially swelled and then dissolved completely, resulting in a brownish solution after 3 days. The solution should comprise **PPG** and a trace amount (< 33 mg) of sodium acetonedicarboxylate—which is not possible to recover due to its low concentration and tendency to undergo decarboxylation under basic conditions.^[^ ^88^, ^89^ ^]^ To recover **PPG**, ethanol was first removed by rotary evaporation, followed by extraction of the resulting mixture with DCM, and removal of the solvent by rotary evaporation, and vacuum drying at 60 °C. This process afforded **PPG** in a yield of 89%. The recovered **PPG** was analyzed by ^1^H NMR spectroscopy, and compared with the starting materials (Figure 4c), confirming its analytical purity.

To evaluate the selectivity and practicality of the depolymerization–separation protocol in more complex waste streams, we next applied the process to a mixture containing **PEADA‐1.2** (0.5 g) and various common plastics: polyethylene (from a plastic bag), polypropylene (from an Eppendorf microtube), polyurethane foam, poly(ethylene terephthalate), and nylon‐6,6 granules (Figure S46a). The mixture was put in 15 mL of ethanol‒5 M NaOH mixture solution (2:1, *v/v*) and treated at 70 °C for 1 day. Under these conditions, **PEADA‐1.2** selectively dissolved while other plastics remained intact as solids, which were then removed through vacuum filtration. Subsequent separation treatment of the resulting filtrate involved removal of ethanol by rotary evaporation, extraction with DCM, neutralization, and removal of the DCM phase (Figure S46a), resulting in the recovery of **PPG** in 91% yield and high purity, as verified the weight and ^1^H NMR spectroscopy (Figure S46b), respectively.

The **PPG** recovered from the depolymerization of both neat **PEADA‐1.2** and its mixture with other plastics was subsequently repurposed to synthesize **PEADA‐1.4** through the reaction with additional **DADC**. Gratifyingly, DMA and tensile testing analyses of the original and re‐synthesized **PEADA‐1.4** films show comparable (thermo)mechanical properties (Figures 4d and S47). These results confirm not only the chemical integrity and functionality of the recovered **PPG**, but also the feasibility of base‐assisted recycling for **EADA**‐containing thermosets.

Given the presence of an enamine motif in **EADA**, the polymer networks should be depolymerizable under acidic conditions as well (Figure S48a).^[^ ^74^, ^84^ ^]^ The above chemical resistance tests showed that **PEADA‐1.2** film is stable in 1 M HCl aqueous solution, which is primarily attributed to the hydrophobic nature of the polymer substrate in preventing effective contact between water and the **EADA** linkages. To induce depolymerization, an acidic organic medium consisting of CHCl_3_ and trifluoroacetic acid (TFA) was adopted. Under these conditions, the **PEADA‐1.2** film underwent gradual depolymerization‐driven dissolution at 60 °C, transforming into a brownish solution after 24 h (Figure S48b). ^1^H NMR analysis of the depolymerized solution confirms cleavage of the enamine bond (Figure S48c). Neutralization of the depolymerized solution with aqueous NaHCO_3_ (10 *w/v*%), followed by solution casting and vacuum drying, removed TFA and produced a new **PEADA‐1.2** film. The re‐synthesized film shows increased *E*’, *T*_g_, and stress at break while a slightly decreased strain at break, as determined by DMA and tensile testing measurements (Figures S48d,e), which probably results from the increase of cross‐link density during the recycling process. These results reflect that by embedding hydrolyzable **EADA** bonds in hydrophobic substrates, a stable yet depolymerizable polymer networks can be accessed.

In addition, the enamine motif is capable of undergoing transamination with free amine functionalities (Figure S49a), enabling thermal reprocessing of technologically relevant thermosets.^[^ ^60^, ^61^ ^]^ Along this line, **Gluten‐5%EADA** was first cut into small pieces and subsequently compression‐molded at 90 °C and a pressure of 20 kN for 20 min. To our delight, it refurnished a homogeneous film (Figure S49b) that possess stable thermomechanical properties, high strength and Young's modulus (Figure S49c,d), demonstrating the good reprocessability. The discrepancy in the (thermo)mechanical properties of the original and reprocessed **Gluten‐5%EADA** is probably attributed to the thermal denaturation of proteins at 90 °C.

## Conclusion

In summary, we have revisited dimethyl acetone‐1,3‐dicarboxylate (**DADC**) chemistry, focusing on its reactions with amines, and found that **DADC** is more reactive toward amines than the conventional imination and amidation reactions. Small‐molecule model and mechanistic studies reveal that the enhanced reactivity of **DADC** is attributed to its synergistic electronic and steric effects; The presence of two esters in the symmetric β‐position of a ketone first promotes the imination of the latter through conjugative and electron‐withdrawing effects, and that affords a β‐enamino functionality. Subsequently, the β‐enamino motif engages one ester through hydrogen bonding, reducing steric hinderance at the remaining ester site and accelerating its amidation. This mechanistic insight was then translated to effectively cross‐linking and/or polymerizing a broad range of amines—both petroleum‐derived and bio‐based—yielding thermoset plastics and elastomers featuring β‐enaminodiamide (**EADA**) linkages, either within the polymer skeleton or as cross‐links. All syntheses proceeded at ≤ 120 °C without the need for catalysts, in sharp contrast to conventional polyamide polycondensations that typically demand high temperatures (≥ 230 °C) and vacuum (0.01–10 mbar). Moreover, the **EADA** bonds are hydrolyzable under basic conditions upon heating at 70 °C, which allows a good recovery of the starting amine feedstocks, as demonstrated in a case study involving the hydrolysis‐induced depolymerization of a poly(β‐enaminodiamide) (**PEADA**). The recovered amines can repolymerize with **DADC**, and afford the **PEADA** of comparable (thermo)mechanical properties to the initially synthesized one. Moreover, the **EADA**‐containing thermosets can also be recycled by acid‐assisted depolymerization and thermal reprocessing, demonstrating the possibility to reuse them through multiple approaches. Overall, our findings underscore the potential of exploiting intrinsic substituent effects within molecular frameworks to develop thermosets that are not only energy‐efficient to manufacture—requiring relatively low temperatures, no vacuum, and catalysts—but also chemically recyclable in a closed‐loop manner. This strategy directly supports sustainable polymer production and advances the vision of a circular plastics economy.

## Supporting Information

The authors have not cited additional references within the Supporting Information.

## Conflict of Interests

The authors declare no conflict of interest.

## Supporting information



Supporting Information

## Data Availability

The data that support the findings of this study are available from the corresponding author upon reasonable request.
