# Supplementary material for: Efficient Synthesis of Chemically Recyclable Polyamides via Substituent Effects‐Enabled Mechanistic Pathway
Source: Angew Chem Int Ed Engl. 2025 Sep 14;64(46):e202516735. doi: 10.1002/anie.202516735 (PMC12603994; doi:10.1002/anie.202516735)
Supplement: Supplementary file 1 — Supporting Information [file ANIE-64-e202516735-s001.pdf]

Support Information

for

Efficient Synthesis of Chemically Recyclable Polyamides via Substituent Effects-Enabled Mechanistic Pathway

Youwei Ma,[1*] Chihui Zheng,[1] Davide Raphaël Bréas,[1] Gadi Slor,[1] Alain P. A. Molleyres,[1] Qiyue Liao,[1] Francesco Stellacci[1,2*]

1. Institute of Materials, École Polytechnique Fédérale de Lausanne (EPFL) Lausanne 1015, Switzerland

2. Institute of Bioengineering, École Polytechnique Fédérale de Lausanne (EPFL) Lausanne 1015, Switzerland

*Correspondence: Y. Ma, youwei.ma@epfl.ch; F. Stellacci, francesco.stellacci@epfl.ch

Contents

Materials and Instrumentation 2

Materials 2

Instrumentation 2

Synthesis and Characterization 4

Investigation of the reaction of **DADC**, **HPO**, **DG**, or **MAA** with **Hea** 4

Determination of the activation energy for the reaction of **DADC** and **Hea** 8

Investigation of the reactions of **DADC** and various small-molecule amines 13

Synthesis of **EADA**-cross-linked thermosets 33

Synthesis and hydrolysis of trihexyl-substituted **EADA** 43

Depolymerization of **PEADA-1.2** and its mixture with commercial plastics 50

Appendix 55

Materials and Instrumentation

Materials

Dimethyl acetone-1,3-dicarboxylate (97%, **DADC**), methyl acetoacetate (99%, **MAA**), dimethyl glutarate (98%, **DG**), *n*-hexylamine (99%, **Hea**), and ethanol-d_6 (99.5%) were purchased from Chemie Brunschwig AG. 4-Heptanone (98%, **HPO**), poly(allylamine hydrochloride) (**PALA·HCl**, M_w = 50 000 g mol^{-1}), cyclooctylamine (97%), benzylamine (99%), 2-methylbenzylamine (96%), aniline (99.5%), 4-methylaniline (98%), and 4-fluoroaniline (99%), 4-nitroaniline (99%), *p*-anisidine (99%), poly(propylene glycol) bis(2-aminopropyl ether) (**PPG**, M_n = 2000 g mol^{-1}), chloroform-*d* (99.8%, $CDCl_3$), 1,12-dodecanediamine (98%, **DDA**), DMSO-d_6 (99.9%), sodium hydroxide (> 95%, NaOH), ethanol (98%), dichloromethane (99.8%, DCM), methanol-d_4 (99.8%), and gluten (from wheat, ≥ 75% protein basis) were obtained from Sigma-Aldrich.

Instrumentation

Nuclear Magnetic Resonance (NMR) Spectroscopy. NMR spectroscopy was carried out at 297.2 K on a Bruker Avance DPX 400 spectrometer at frequencies of 400.19 MHz for ^{1}H nuclei and 100.63 MHz for ^{13}C nuclei. Spectra were calibrated to the residual solvent peak of $CDCl_3$ (7.26 ppm ^{1}H NMR), D_2O (4.79 ppm ^{1}H NMR), DMSO-d_6 (2.50 ppm ^{1}H NMR; 39.52 ppm ^{13}C NMR), methanol-d_4 (4.78 ppm, 3.31 ppm ^{1}H NMR), and ethanol-d_6 (5.19 ppm, 3.56 ppm, and 1.11 ppm ^{1}H NMR). Data were evaluated with the MestReNova software suite (v 12.0) and all chemical shifts (δ) are reported in parts per million (ppm) relative to tetramethylsilane with coupling constant (J) in Hz (multiplicity: s = singlet, d = doublet, t = triplet, q = quartet, m = multiplet, dd = double doublet).

Electrospray-Ionization (ESI) High-Resolution Mass Spectrometry (HRMS) data were acquired on a Q-ToF Ultima mass spectrometer (Waters) operated in positive mode. Data were processed using Mmass 5.5.0 software.

Fourier Transform Infrared (FTIR) Spectroscopy. FTIR was carried out on a Bruker Tensor 27 spectrometer. Samples were analyzed in attenuated total reflectance (ATR) mode using a diamond crystal.

Thermogravimetric analyses (TGA) were performed on TGA 8000 instrument (Perkin Elmer) with an autosampler. The temperature ranged from 25 °C to 800 °C with a heating rate of 10 °C min^{-1}. Tests were carried out under nitrogen atmosphere with a flow rate of 40 mL min^{-1}.

Differential Scanning Calorimetry (DSC) measurements were performed using differential scanning calorimeter (DSC Q100, TA Instruments) under nitrogen atmosphere operating at a heating/cooling rate of 10 °C min^{-1} in the temperature range of –70 to –40 °C using a sample mass of ca. 5 mg.

Dynamic Mechanical Analyses (DMA) were performed on the TA Instrument Model Q800 in tension mode. The temperature ranged from –70 to 70 °C for **PEADA-*n***, or from –50 to 200 °C for **PALA-** and

gluten-based films, with a heating rate of 5 °C min^{-1}, a frequency of 1 Hz, and a strain amplitude of 2% (for **PEADA-*n***) or 0.5% (for **PDDA-**, **PALA-** and gluten-based films).

Tensile testing. Stress–strain measurements were carried out at 20 mm min^{-1} with a Zwick/Roell Z010 tensile tester equipped with a 50 N load cell. Uniaxial tensile tests were carried out with dog–bone-shaped samples with dimensions of 40 × 5 × 0.2 mm (length × width × thickness) that were cut from the solution-casted films according to ASTM D1708. The testing procedure followed the standard method described in ASTM D1708.

Solubility experiment. The weighed samples (M_{initial}) were immersed in dichloromethane (DCM) and shaken in a shaking bed for 24 h. The DCM was changed every 6 h. The solvent was carefully removed with a syringe, and the surface was wiped with a tissue after swelling, after which the samples were weighed (M_{swelling}). The samples were dried under vacuum at 60 °C for 24 h to ensure complete removal of the solvent from the material. After drying, the samples were weighed again (M_{drying}). The swelling ratio was defined as $\mathrm{M_{swelling}/M_{initial}}$, whereas the gel fraction was defined as $M_{\mathrm{drying}}/M_{\mathrm{initial}}$.

Density Functional Theory (DFT) calculations were carried out using the Gaussian 16 software package at the M06-2X/6-311++G(d,p) level of theory. Transition states were located through constrained optimizations and further refined to ensure a single imaginary frequency along the reaction coordinate. All other structures were optimized using standard geometry optimization settings, and the frequency calculations were conducted to verify the nature of the stationary point and obtain zero-point energy (ZPE) corrections. For the hydrolysis process, implicit solvation effects were taken into consideration using the Solvation Model based on Density (SMD) with water as the solvent. All reported energies include ZPE corrections obtained from vibrational analysis. Structures optimized using the SMD solvation model are indicated by '(SMD, water)' following the compound name (see Appendix).

Synthesis and Characterization

Investigation of the reaction of DADC, HPO, DG, or MAA with Hea

Dimethyl acetone-1,3-dicarboxylate (**DADC**) features two types of reactive chemical motifs, including β-ketone and methyl carboxylate, which exhibit distinct reactivity toward amines. The reaction of amine toward the β-ketone motif at room temperature (i.e., 22 °C) was investigated first according to the following procedures. **DADC** (5.2 mg, 0.03 mmol) was mixed with 550 µL of $CDCl_3$ in a 2 mL Eppendorf microtube. *n*-Hexylamine (**Hea**; 30 mg, 0.3 mmol) was then added to the microtube, and shaken several times. The resulting mixture was immediately transferred to a NMR tube and subjected to analysis. The ^{1}H NMR spectra measured at different time intervals, and the protons assignment before and after the reaction are shown in Figure S1. The formed compounds during the reaction include β-iminodiester (**IDE**) and β-enaminodiester (**EADE**).

For the experiment without the use of $CDCl_3$, **DADC** (348 mg, 2 mmol) and **Hea** (2.02 g, 20 mmol) were added to a 20 mL small vial with a magnetic stir bar. After 24 h of reaction at room temperature, 30 µL aliquot of the mixture solution were taken out and sent for ^{1}H NMR analysis. The ^{1}H NMR spectrum obtained was compared with that obtained from the experiment conducted in the presence of $CDCl_3$, and shown in Figure S7.

The reaction of amine and 4-heptanone (**HPO**) was investigated by mixing **HPO** (3.5 mg, 0.03 mmol) and **Hea** (30 mg, 0.3 mmol) in 550 µL of $CDCl_3$ for ^{1}H NMR analysis at room temperature. The ^{1}H NMR spectra measured at the initial time and 24 h are shown in Figure S2.

The reaction of amine toward the methyl carboxylate moiety of **DADC** was investigated at high temperatures, and followed the next protocol. In a 20 mL small vial with a magnetic stir bar, 348 mg of **DADC** (2 mmol) was mixed with 2.02 g of **Hea** (20 mmol). The mixture was heated to 60 °C, 80 °C, 100 °C, or 120 °C. After different reaction time intervals, 30 µL aliquots of the reaction mixture were taken out with a pipet and dissolved in 550 µL of $CDCl_3$ for ^{1}H NMR analysis. ^{1}H NMR spectra of these solutions measured at different time intervals and the protons assignment for the reaction are shown in Figures S3, and the data were used to establish the reaction kinetics. The fraction of the compound thus produced, β-enaminodiamide (**EADA**), was calculated by integration of the ^{1}H NMR signals (Figures S3), according to the equation:

Fraction **EADA** (% **EADA**) = [**EADA**]/([**EADA**] + [**DADC**] + [**EADE**]) = peak 4'/[peak 4'+(peak 1'+ peak 1)/3]

The extent of conversion at specific temperature is plotted in Figure 1(b).

The reaction of amine and dimethyl glutarate (**DG**) was investigated following the same protocol, but only at 120 °C. First, **DG** (0.32 g, 2 mmol) and **Hea** (2.02 g, 20 mmol) were added to a 20 mL small vial. A small amount of the resulting mixture (30 µL) was first taken out for ^{1}H NMR measurement before it was heated to 120 °C for 1 h. After the reaction, another 30 µL aliquot of the reaction mixture was taken out and mixed with 550 µL of $CDCl_3$ for ^{1}H NMR analysis. The ^{1}H NMR spectra before and after the reaction are shown in Figure S4.

The reaction of amine and methyl acetoacetate (**MAA**) was studied first at room temperature and then at 120 °C. In a 20 mL small vial with a magnetic stir bar, **MAA** (0.23 g, 2 mmol) and **Hea** (2.02 g, 20 mmol) were added. Under room temperature, 30 µL aliquots of the mixture solution were taken out and sent for ^{1}H NMR analysis in the initial and 24 h of reaction times. The mixture was subsequently heated to 120 °C and kept for 1 h, before 30 µL of the solution was collected for ^{1}H NMR and HRMS analyses. The relevant ^{1}H NMR and HRMS spectra during the reaction are shown in Figures S8 and S9.

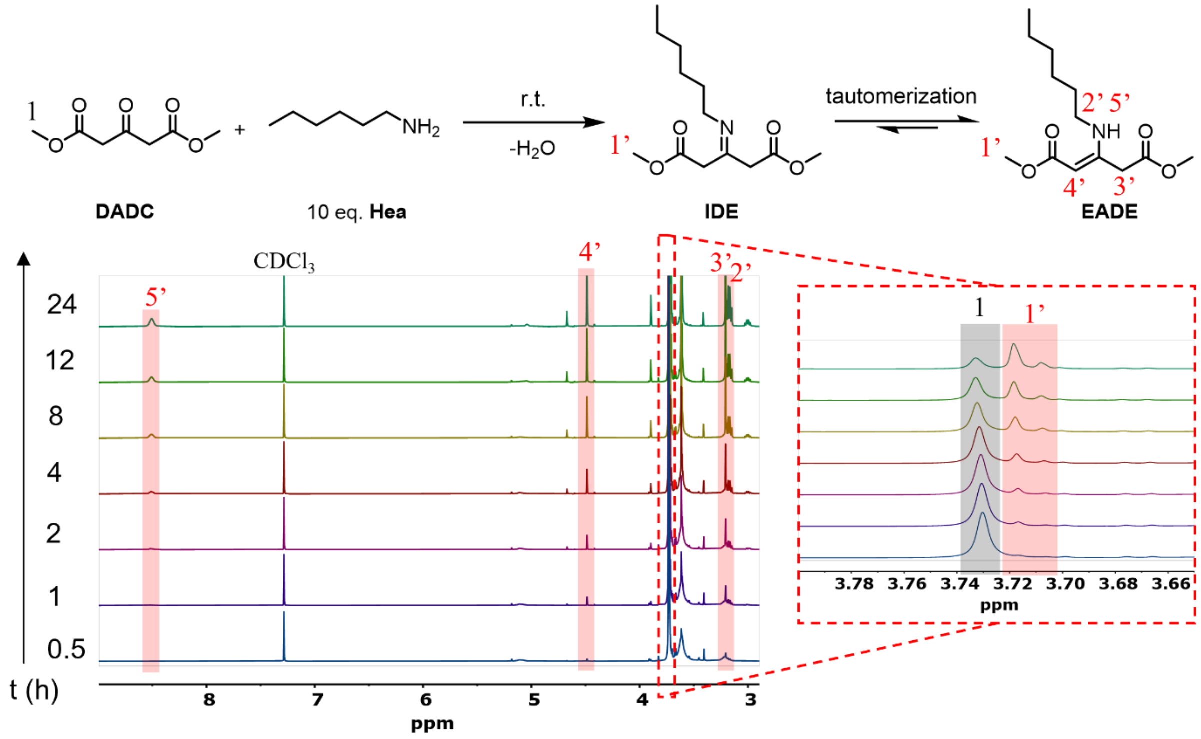

Figure S1. Scheme showing the reaction of **DADC** and 10 eq. of **Hea** at room temperature to afford **IDE** and its tautomer **EADE**, with the assignment of the key protons shown in the ^{1}H NMR spectra (Top). Evolution of the ^{1}H NMR signals ($CDCl_3$, 400 MHz) of the reaction mixture of **DADC** (5.2 mg) and **Hea** (30 mg) at room temperature over time (as indicated).

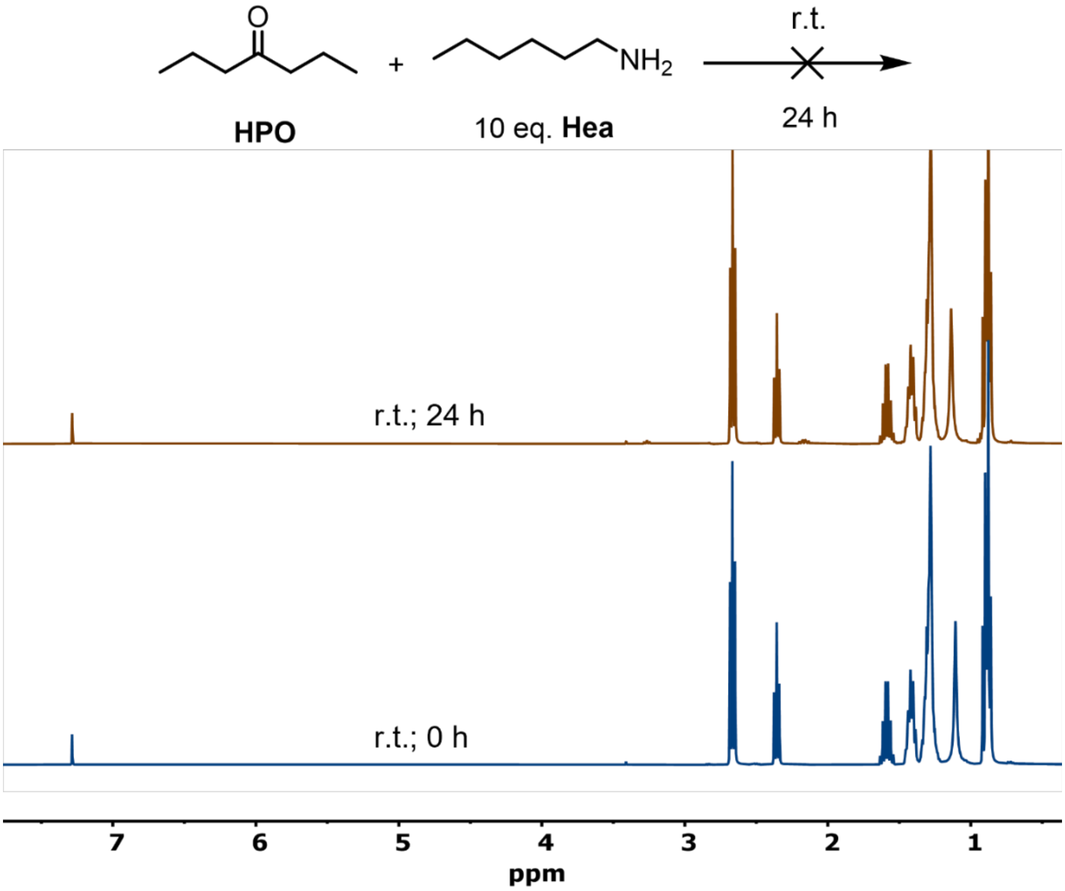

Figure S2. Scheme of the reaction mixture of **HPO** and 10 eq. of **Hea** at room temperature, and its ^{1}H NMR spectra ($CDCl_3$, 400 MHz) measured at the initial and 24 h-reaction times (as indicated).

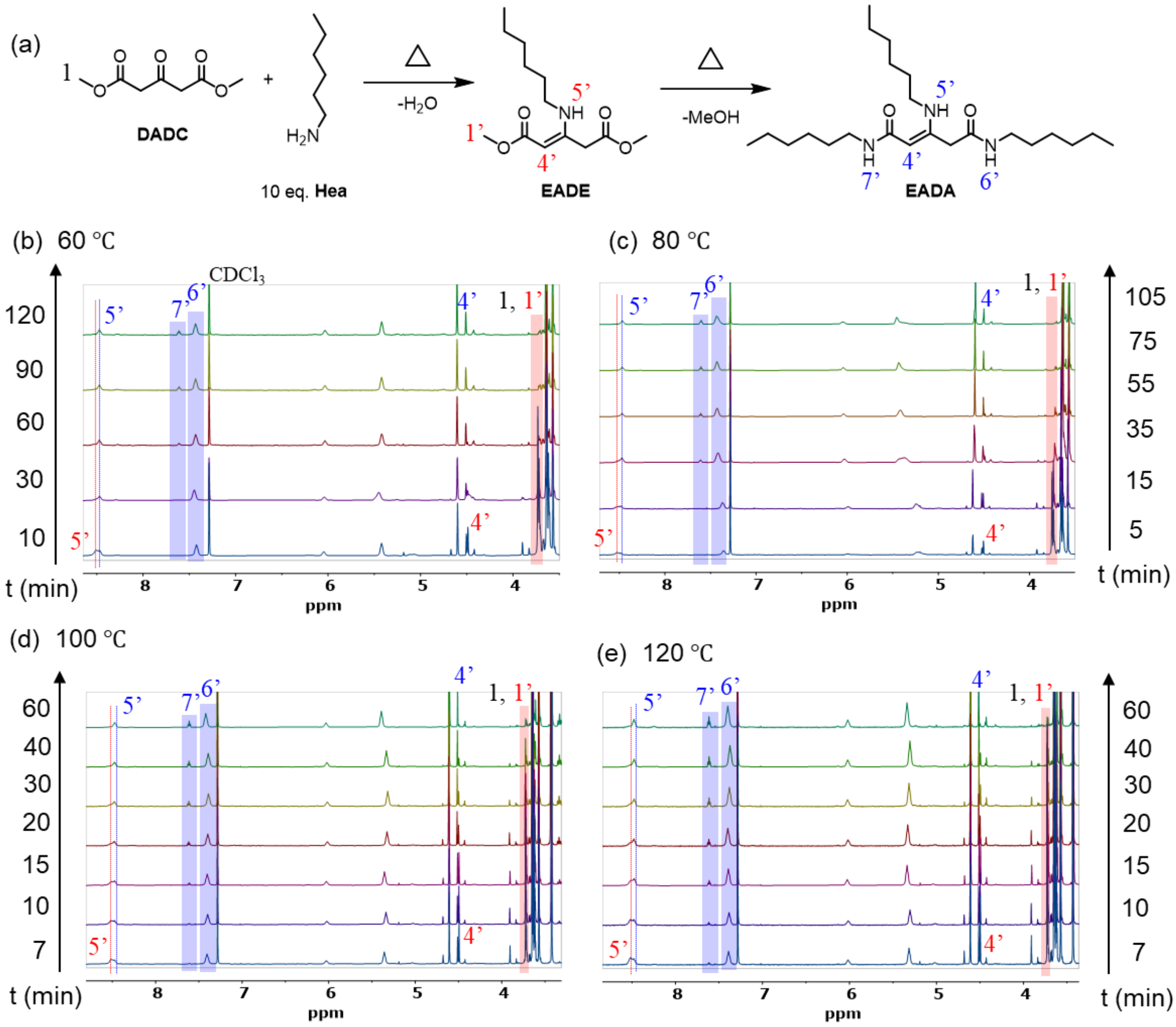

Figure S3. (a) Scheme showing the reaction of **DADC** and 10 eq. of **Hea** to first form **EADE** and then **EADA** upon thermal treatment, with the assignment of key protons for the integration of the ^{1}H NMR signals. Evolution of the ^{1}H NMR signals ($CDCl_3$, 400 MHz) of the reaction mixture (30 μL) of **DADC** and **Hea**. The samples were collected at different reaction times (as indicated), and the reactions were carried out at (b) 60 °C, (c) 80 °C, (d) 100 °C, and (e) 120 °C.

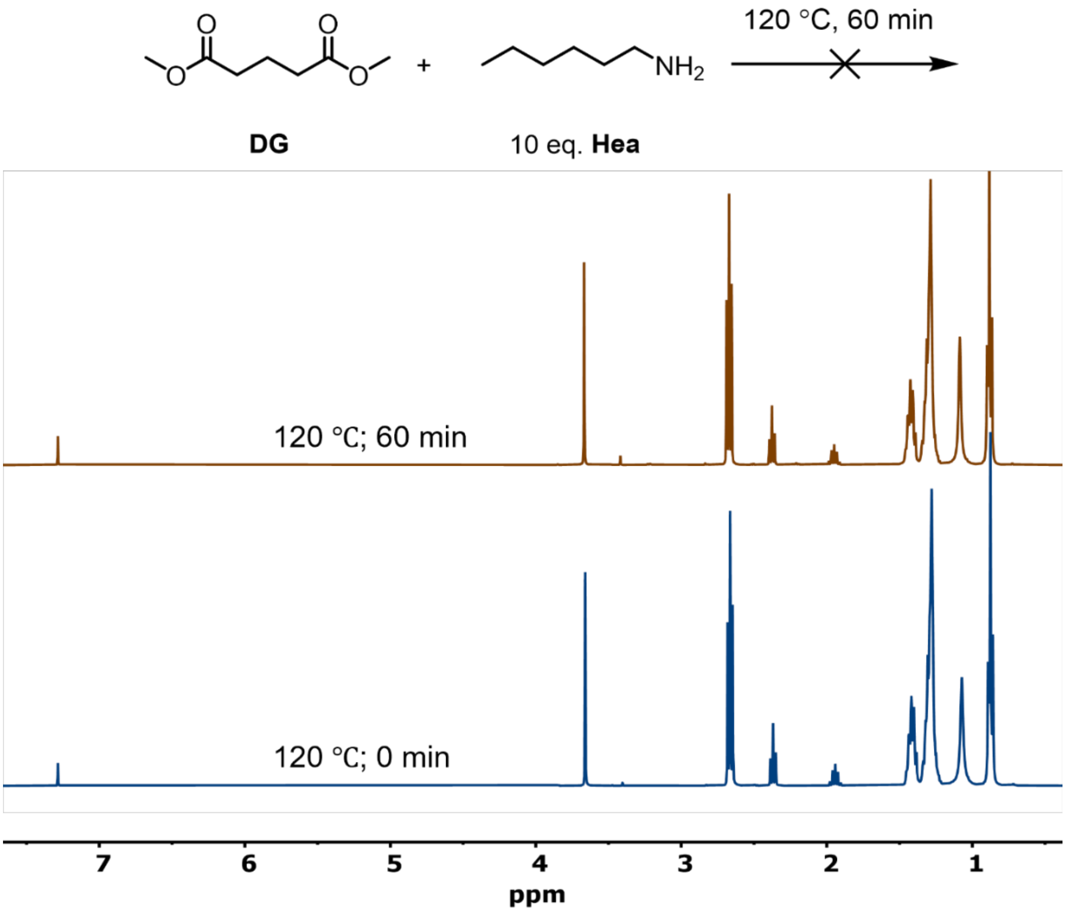

Figure S4. Scheme of the reaction mixture of **DG** and 10 eq. of **Hea** at 120 °C, and its ^{1}H NMR spectra ($CDCl_3$, 400 MHz) measured before and after 60 min of reaction time (as indicated).

Determination of the activation energy for the reaction of DADC and Hea

The rate constants (k) for the reaction of **DADC** and 10 eq. of **Hea** at different temperatures (60, 80, 100, and 120 °C) were obtained by monitoring the fraction of **EADA** in the reaction mixtures under pseudo first-order conditions (large excess of **Hea**) (Supplementary Equation 1). The activation energy E_a for the reaction was determined by plotting ln(k) *vs*. T^{-1} (Supplementary Equation 2).

Supplementary Equation 1:

ln(fraction **EADA**) = –ln(1 – %**EADA**) = –k(t), the fitting curves are shown in Figure S5.

Supplementary Equation 2:

$\ln(k) = \ln(A) - E_a/RT$, the fitting curve is shown in Figure 1(c).

where T is the temperature expressed in Kelvin, and R = 8.31 J K^{-1} mol^{-1}. The values for k and E_a thus determined are shown in Table S1.

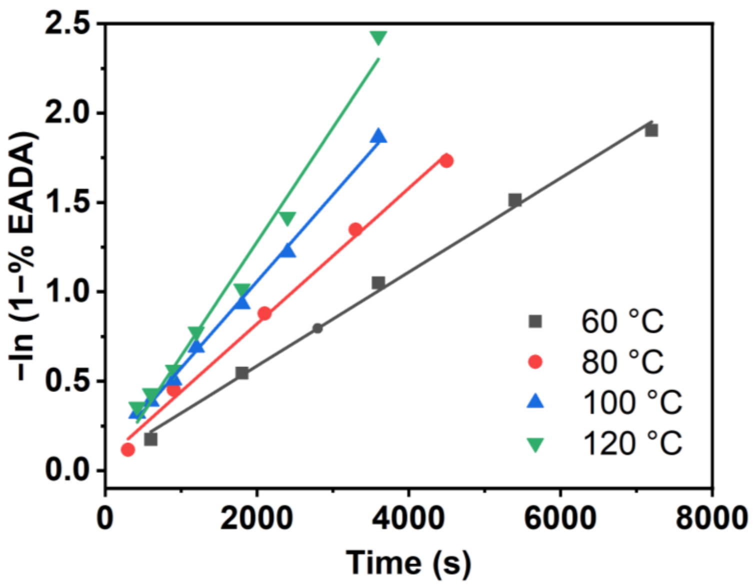

Figure S5. Fitting curves for the conversion of **DADC** into a **EADA** derivative at 60 ºC (black), 80 ºC (red), 100 ºC (blue), and 120 ºC (green). Note: the data point at 80 ºC and 6300s (associated to the ^{1}H NMR spectrum in Figure S3c) was not included due to its large deviation from the fitting curve, probably induced by experimental error. The slight deviation of the data points at 120 ℃ is probably attributed to the evaporation of **Hea** at such a high temperature.

Table S1. Pseudo-first-order rate constants k and activation energy E_a for the reaction of **DADC** and **Hea** (10 eq.).

**Temperature (ºC)**	**Rate constant *k* (s^{-1})**	**Activation energy E_a (kJ mol^{-1})**
60	$2.63 \times 10^{-4} \pm 9.38 \times 10^{-6}$	15.7 ± 0.7
80	$3.79 \times 10^{-4} \pm 1.58 \times 10^{-5}$	
100	$4.84 \times 10^{-4} \pm 1.05 \times 10^{-5}$	
120	$6.38 \times 10^{-4} \pm 3.89 \times 10^{-5}$	

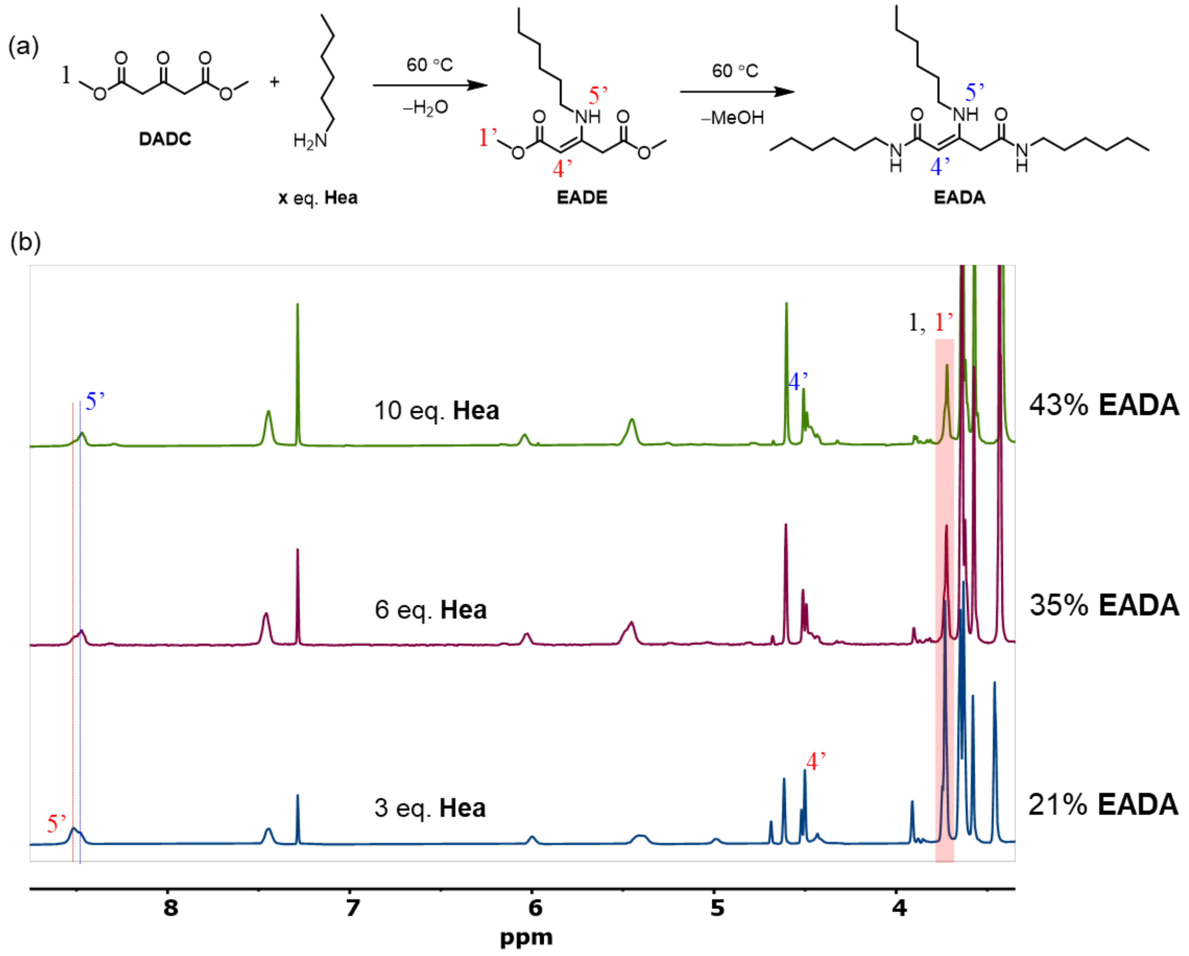

Figure S6. (a) Scheme showing the reaction of **DADC** and x eq. of **Hea** to first form **EADE** and then **EADA** upon thermal treatment, with the assignment of key protons for the integration of the ^{1}H NMR signals. (b) ^{1}H NMR spectra ($CDCl_3$, 400 MHz) of the reaction mixture (30 µL) of **DADC** and 3 eq., 6eq., and 10 eq. of **Hea** (as indicated).

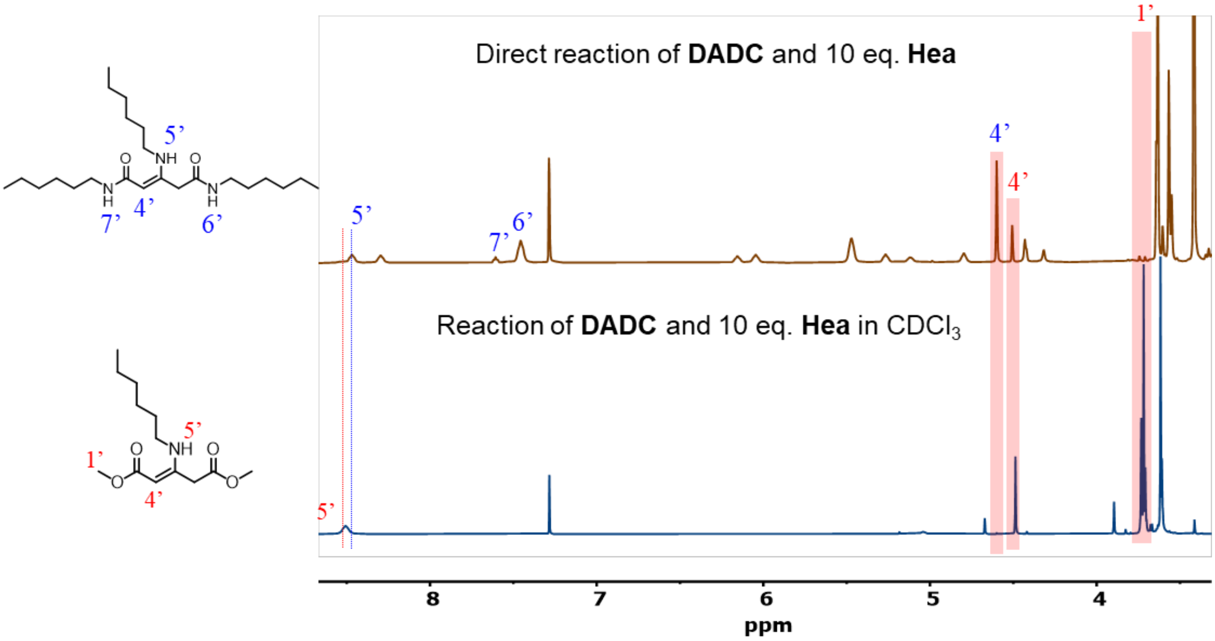

Figure S7. ^{1}H NMR spectra ($CDCl_3$, 400 MHz) of the reaction mixture (30 µL) of **DADC** and 10 eq. of **Hea**, with the reaction taking place in the presence (bottom) or absence (top) of $CDCl_3$.

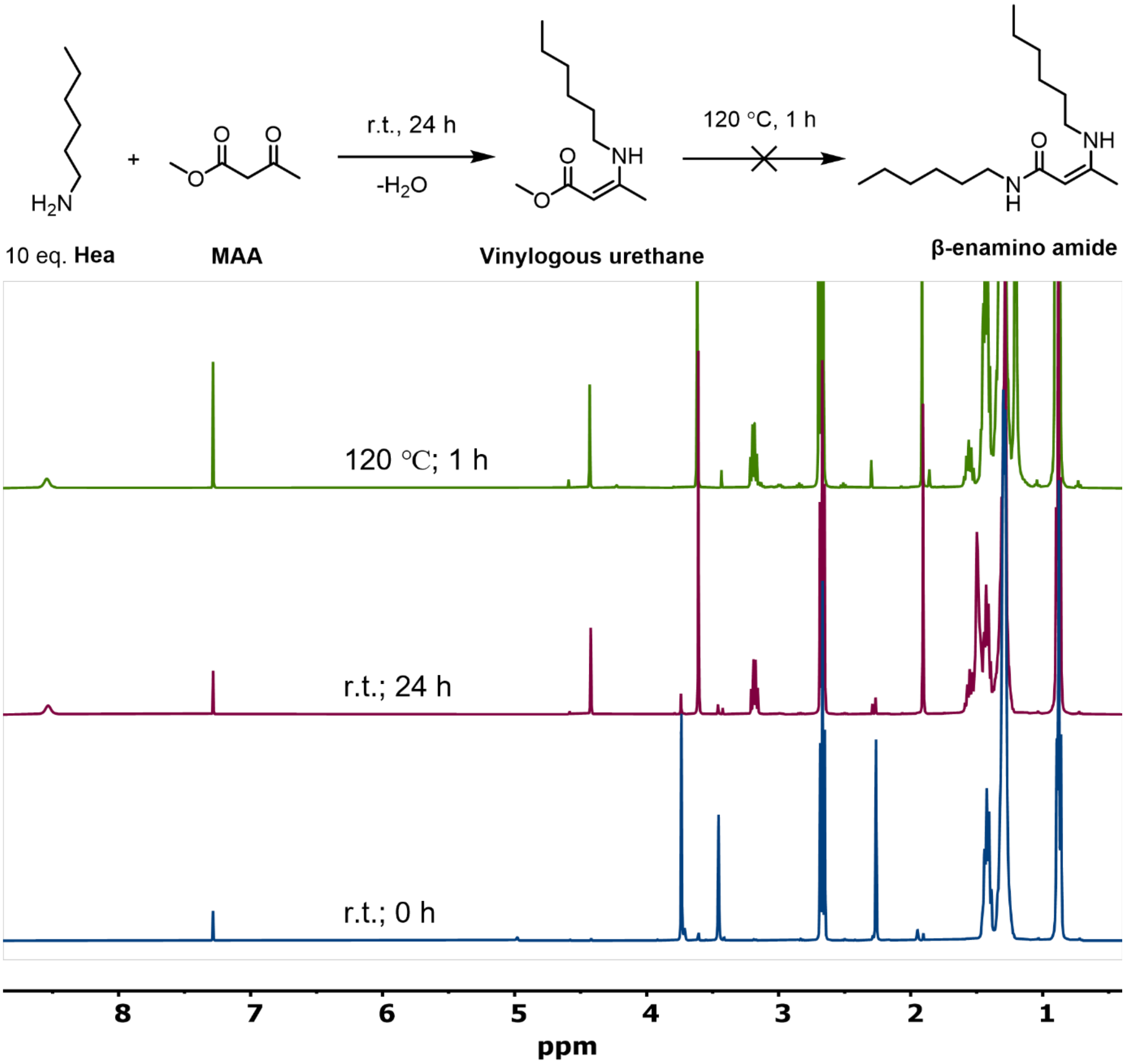

Figure S8. Scheme of the reaction mixture of **MAA** and 10 eq. of **Hea** first at room temperature and then at 120 °C, and its ^{1}H NMR spectra ($CDCl_3$, 400 MHz) measured at the initial and 24 h-reaction times under room temperature, and also in 1 h of reaction at 120 °C (as indicated).

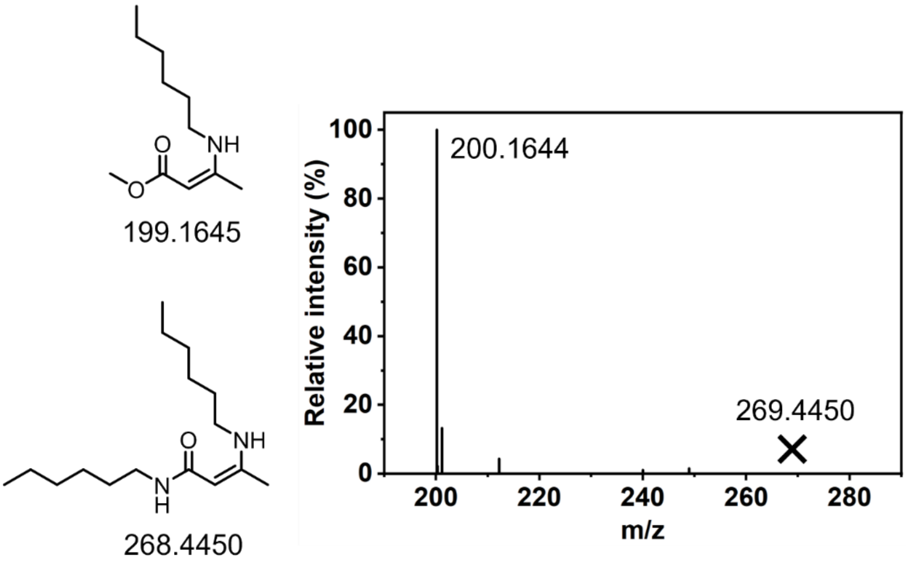

Figure S9. HRMS spectrum of the mixture of **MAA** and **Hea** (10 eq.) upon thermal treatment first at room temperature for 24 h and then at 120 °C for 1 h. HRMS (ESI/QTOF) m/z: $[M + H]^+$ Calcd for $C_{11}H_{22}NO_2^+$ 200.1645; Found 200.1644. $[M + H]^+$ Calcd for $C_{16}H_{32}N_2O^+$ 269.4450, not found.

Investigation of the reactions of DADC and various small-molecule amines

The reactions of **DADC** and various small-molecules amines including **Hea**, cyclooctylamine, benzylamine, 2-methylbenzylamine, aniline, 4-methylaniline, 4-fluoroaniline, 4-nitroaniline, and *p*-anisidine were studied at 120 °C for 12 h. The molar feeding ratio of amine to **DADC** was set as 3. Taking the reaction of **DADC** and **Hea** as the example, first, **DADC** (348 mg, 2 mmol) and **Hea** (607 mg, 6 mmol) were added in a 20 mL small vial with a magnetic stir bar. The mixture was subsequently heated to 120 °C and kept for 12 h, before 30 μL of the solution was collected for ^{1}H NMR and HRMS analyses. The relevant ^{1}H NMR and HRMS spectra for all the reactions are shown in Figures S10–S27.

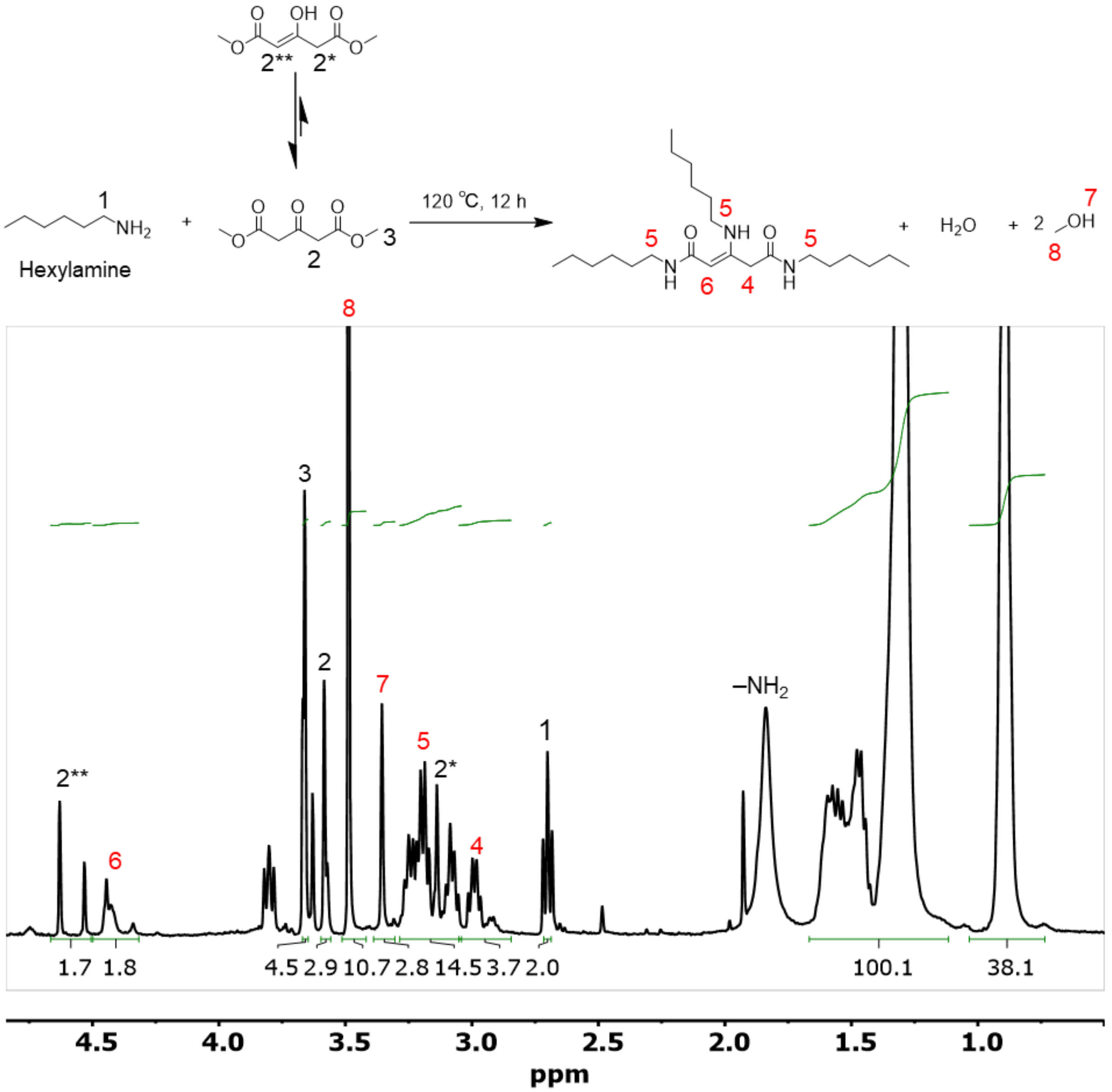

Figure S10. Scheme and ^{1}H NMR spectrum ($CDCl_3$, 400 MHz) of the reaction mixture of **DADC** and 3 eq. of **Hea** upon thermal treatment at 120 °C for 12 h.

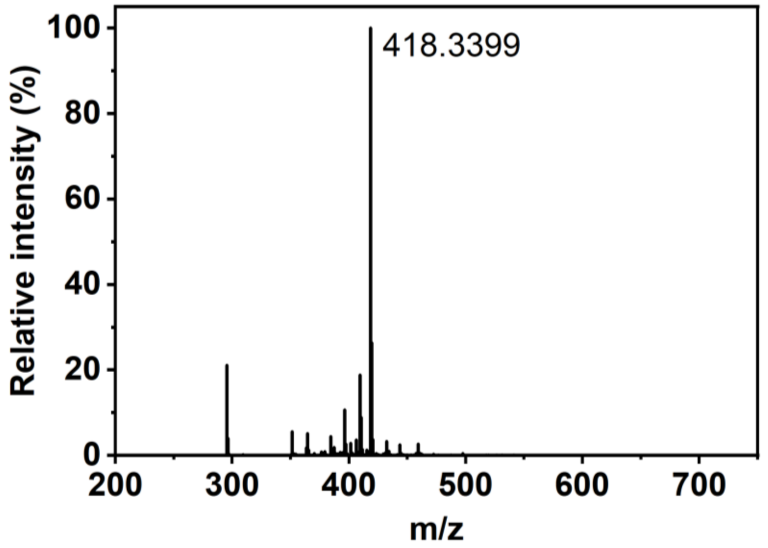

Figure S11. HRMS spectrum of the mixture of **DADC** and **Hea** (3 eq.) upon thermal treatment at 120 ℃ for 12 h. HRMS (ESI/QTOF) m/z: $[M + Na]^+$ Calcd for $C_{23}H_{45}N_3NaO^{2+}$ 418.3404; Found 418.3399.

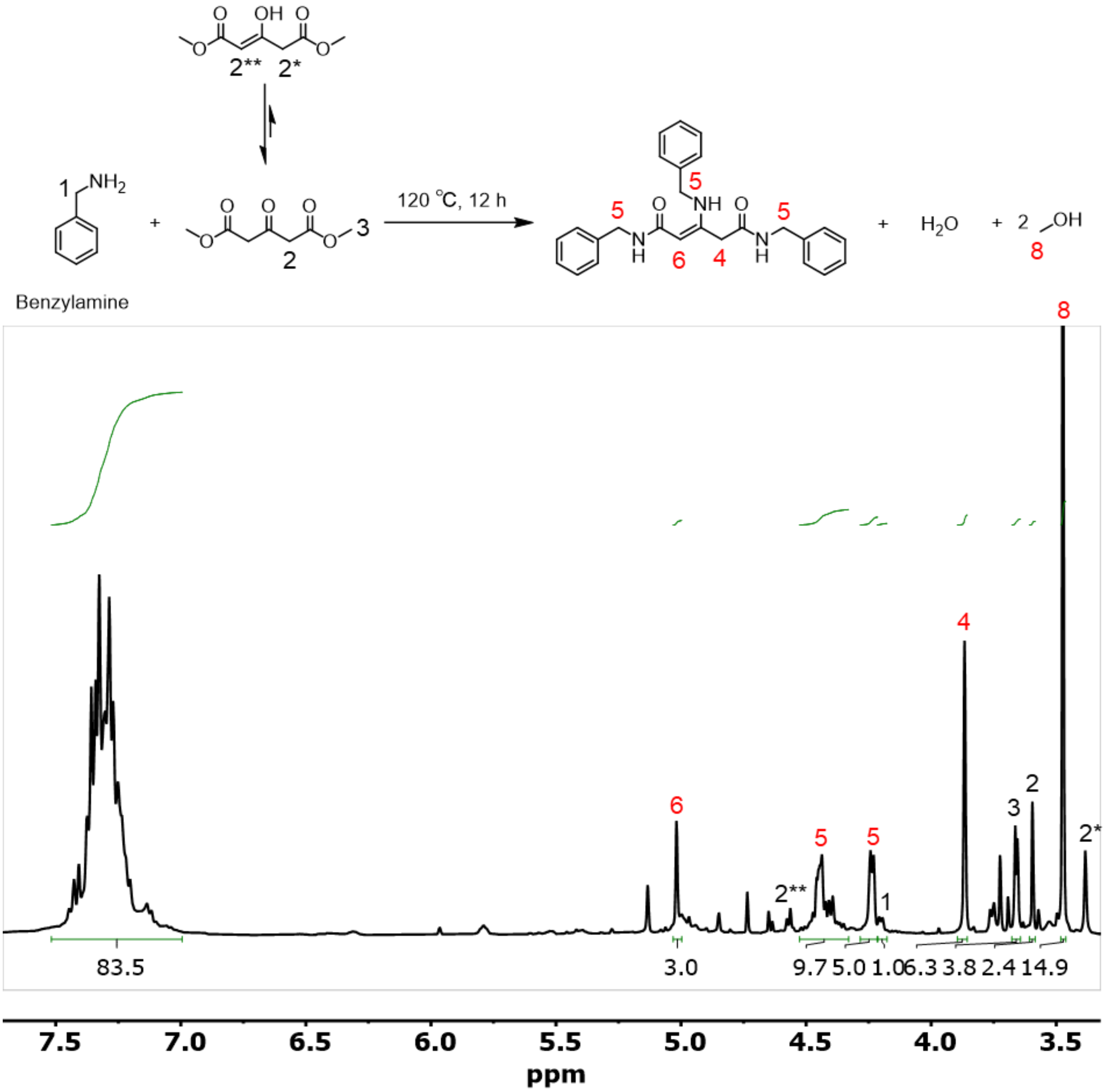

Figure S12. Scheme and ^{1}H NMR spectrum ($CDCl_3$, 400 MHz) of the reaction mixture of **DADC** and 3 eq. of benzylamine upon thermal treatment at 120 °C for 12 h.

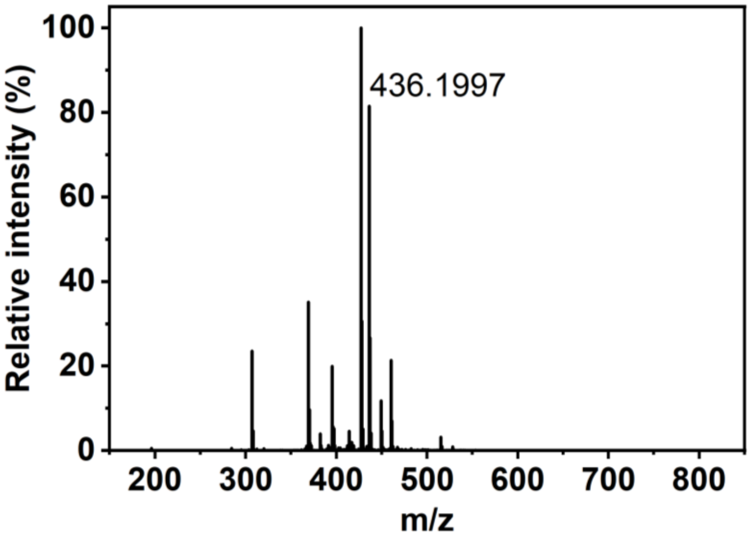

Figure S13. HRMS spectrum of the mixture of **DADC** and benzylamine (3 eq.) upon thermal treatment at 120 °C for 12 h. HRMS (ESI/QTOF) m/z: [M + Na]$^+$ Calcd for $C_{26}H_{27}N_3NaO^{2+}$ 436.1995; Found 436.1997.

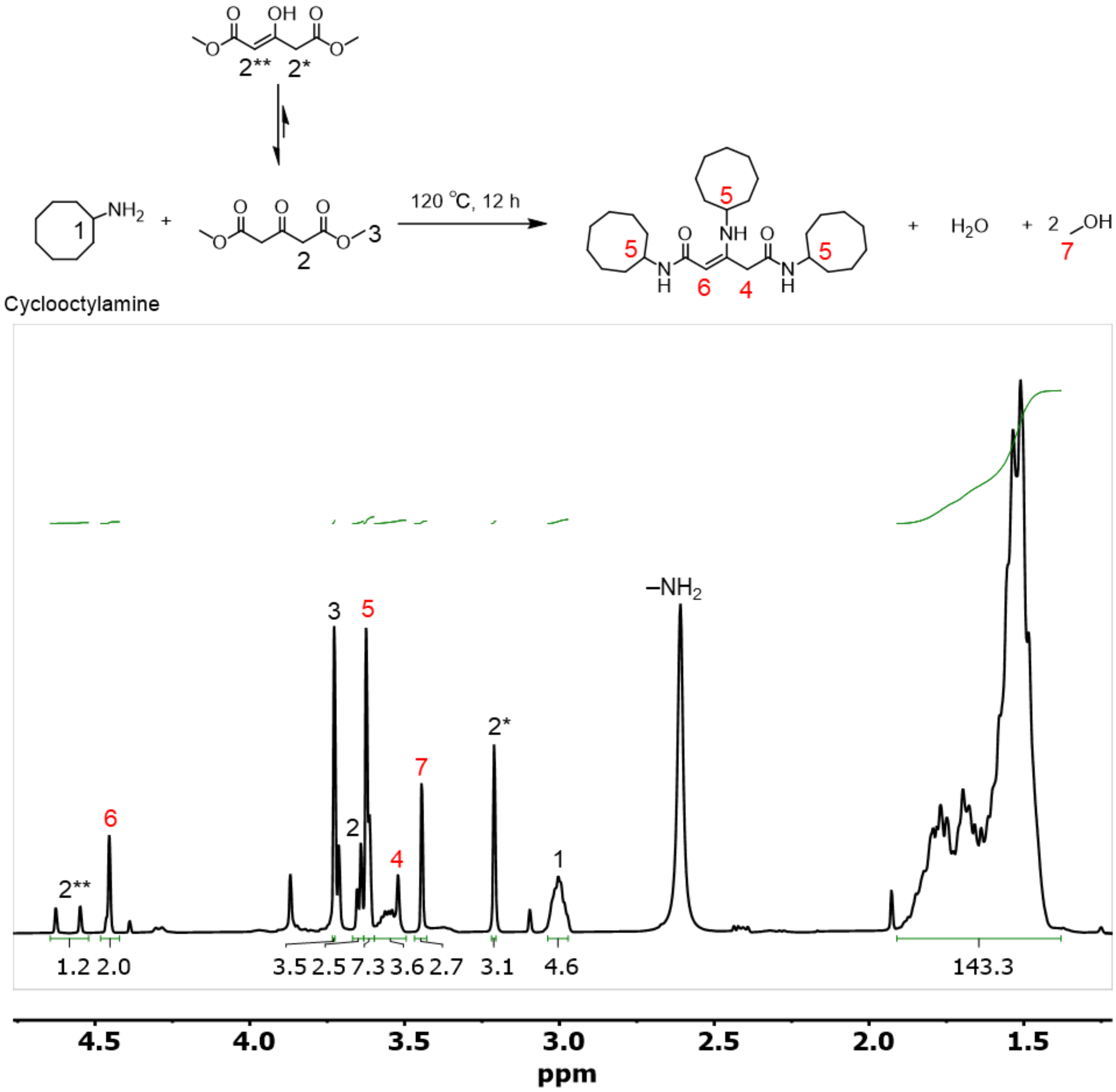

Figure S14. Scheme and ^1H NMR spectrum ($CDCl_3$, 400 MHz) of the reaction mixture of **DADC** and 3 eq. of cyclooctylamine upon thermal treatment at 120 °C for 12 h.

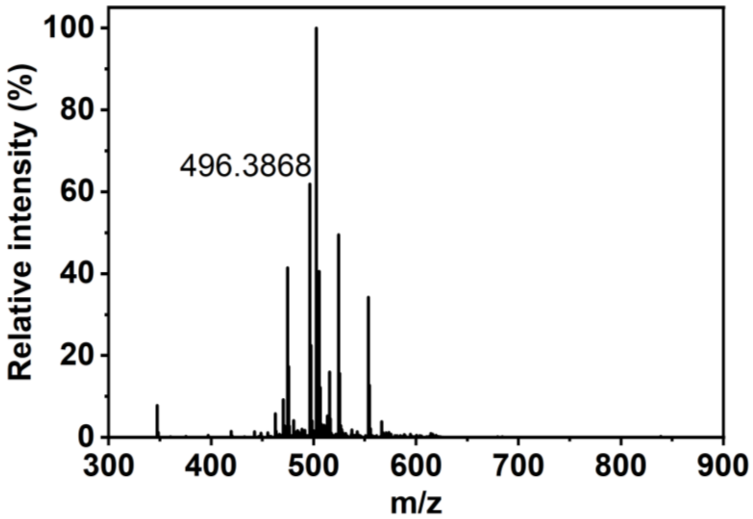

Figure S15. HRMS spectrum of the mixture of **DADC** and cyclooctylamine (3 eq.) upon thermal treatment at 120 °C for 12 h. HRMS (ESI/QTOF) m/z: $[M + Na]^+$ Calcd for $C_{29}H_{51}N_3NaO^{2+}$ 496.3873; Found 496.3868.

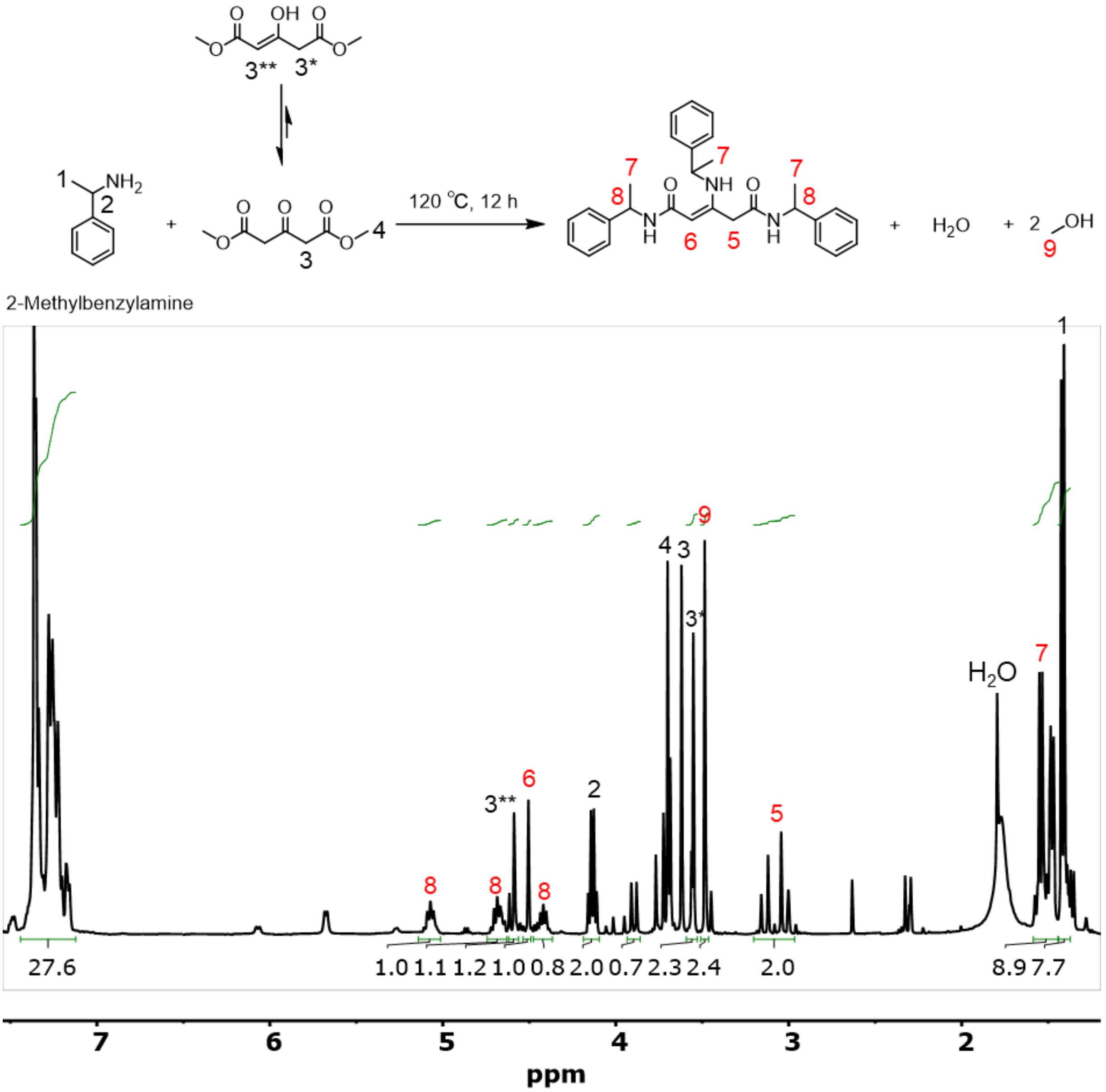

Figure S16. Scheme and ^{1}H NMR spectrum ($CDCl_3$, 400 MHz) of the reaction mixture of **DADC** and 3 eq. of 2-methylbenzylamine upon thermal treatment at 120 °C for 12 h.

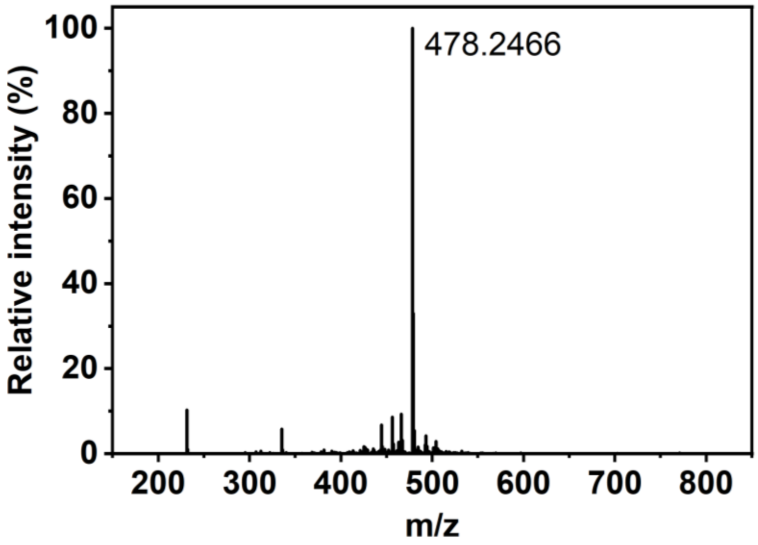

Figure S17. HRMS spectrum of the mixture of **DADC** and 2-methylbenzylamine (3 eq.) upon thermal treatment at 120 °C for 12 h. HRMS (ESI/QTOF) m/z: $[M + Na]^+$ Calcd for $C_{29}H_{33}N_3NaO^{2+}$ 478.2465; Found 478.2466.

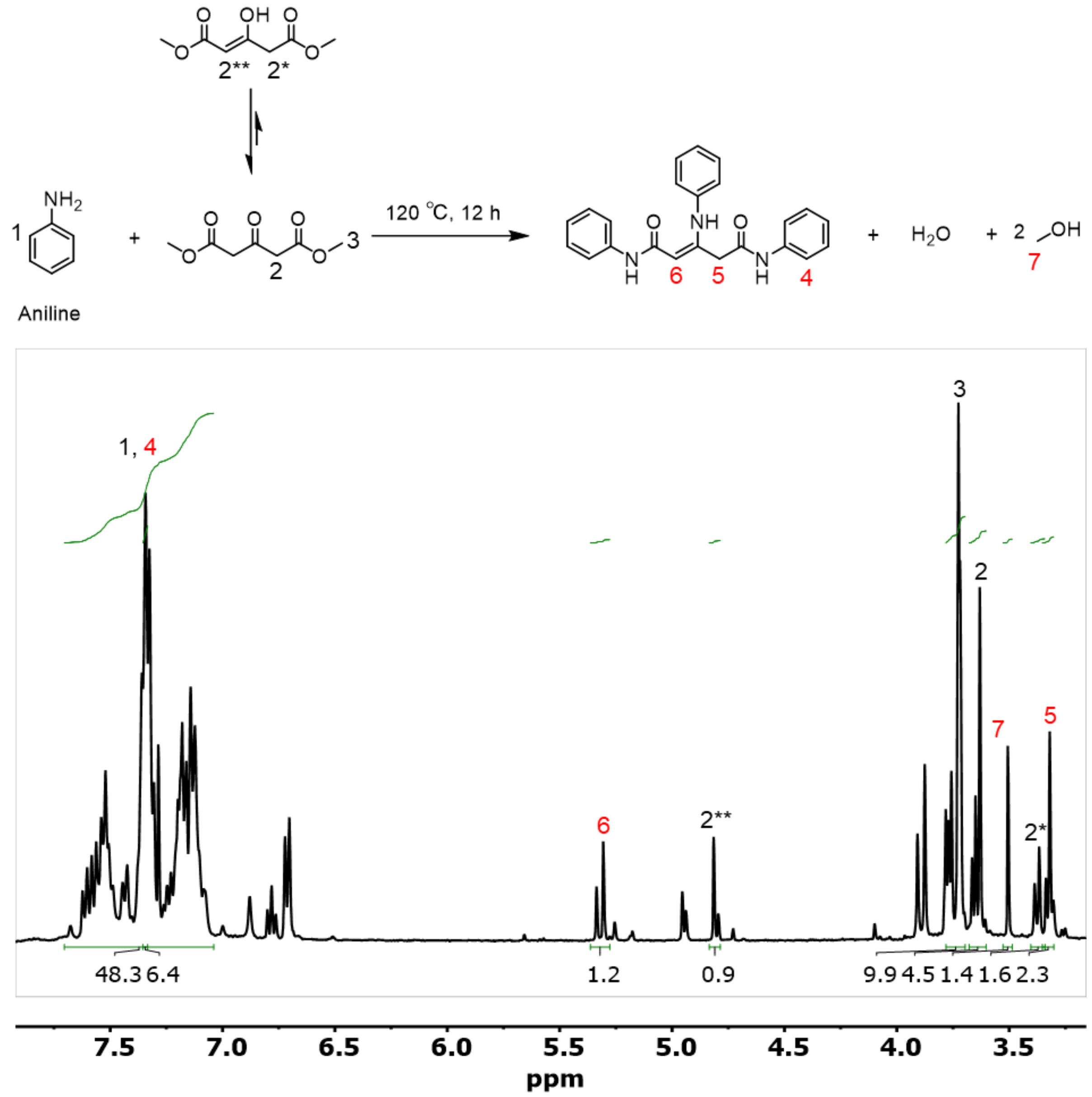

Figure S18. Scheme and ^{1}H NMR spectrum ($CDCl_3$, 400 MHz) of the reaction mixture of **DADC** and 3 eq. of aniline upon thermal treatment at 120 °C for 12 h.

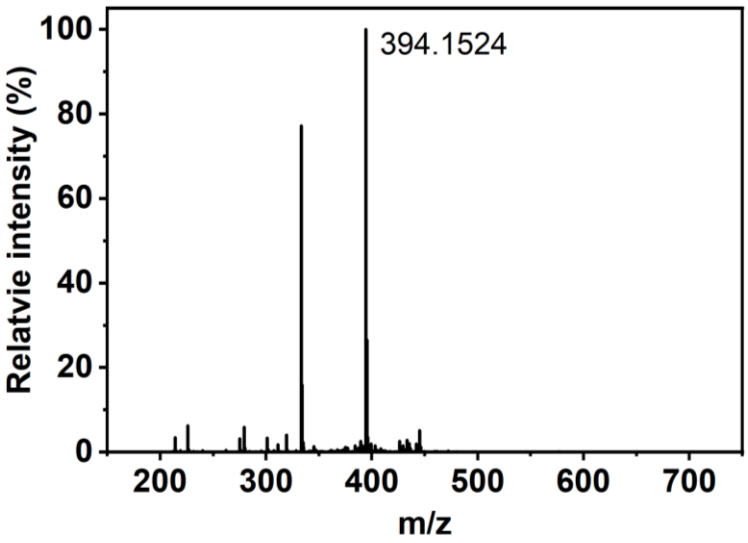

Figure S19. HRMS spectrum of the mixture of **DADC** and aniline (3 eq.) upon thermal treatment at 120 °C for 12 h. HRMS (ESI/QTOF) m/z: $[M + Na]^+$ Calcd for $C_{23}H_{21}N_3NaO^{2+}$ 394.1526; Found 394.1524.

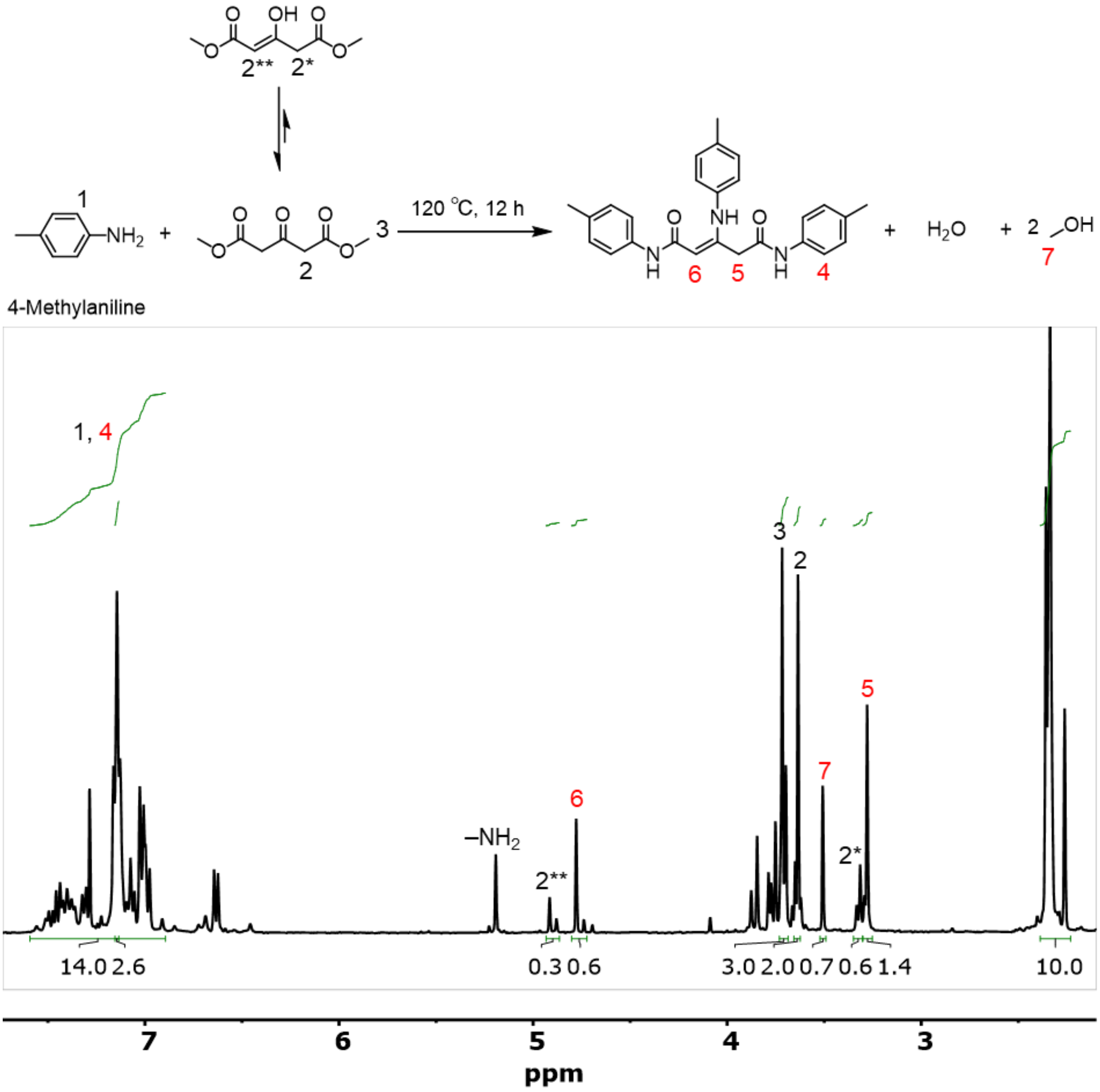

Figure S20. Scheme and ^1H NMR spectrum ($CDCl_3$, 400 MHz) of the reaction mixture of **DADC** and 3 eq. of 4-methylaniline upon thermal treatment at 120 °C for 12 h.

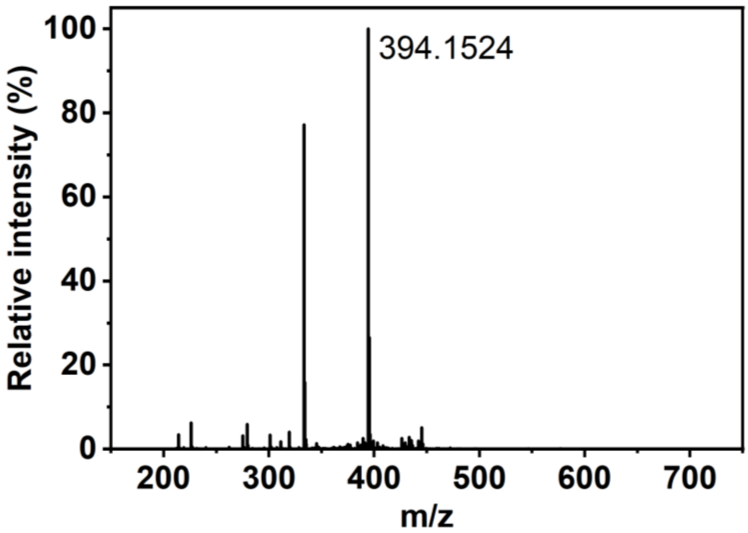

Figure S21. HRMS spectrum of the mixture of **DADC** and 4-methylaniline (3 eq.) upon thermal treatment at 120 ℃ for 12 h. HRMS (ESI/QTOF) m/z: $[M + Na]^+$ Calcd for $C_{23}H_{21}N_3NaO^{2+}$ 394.1526; Found 394.1524.

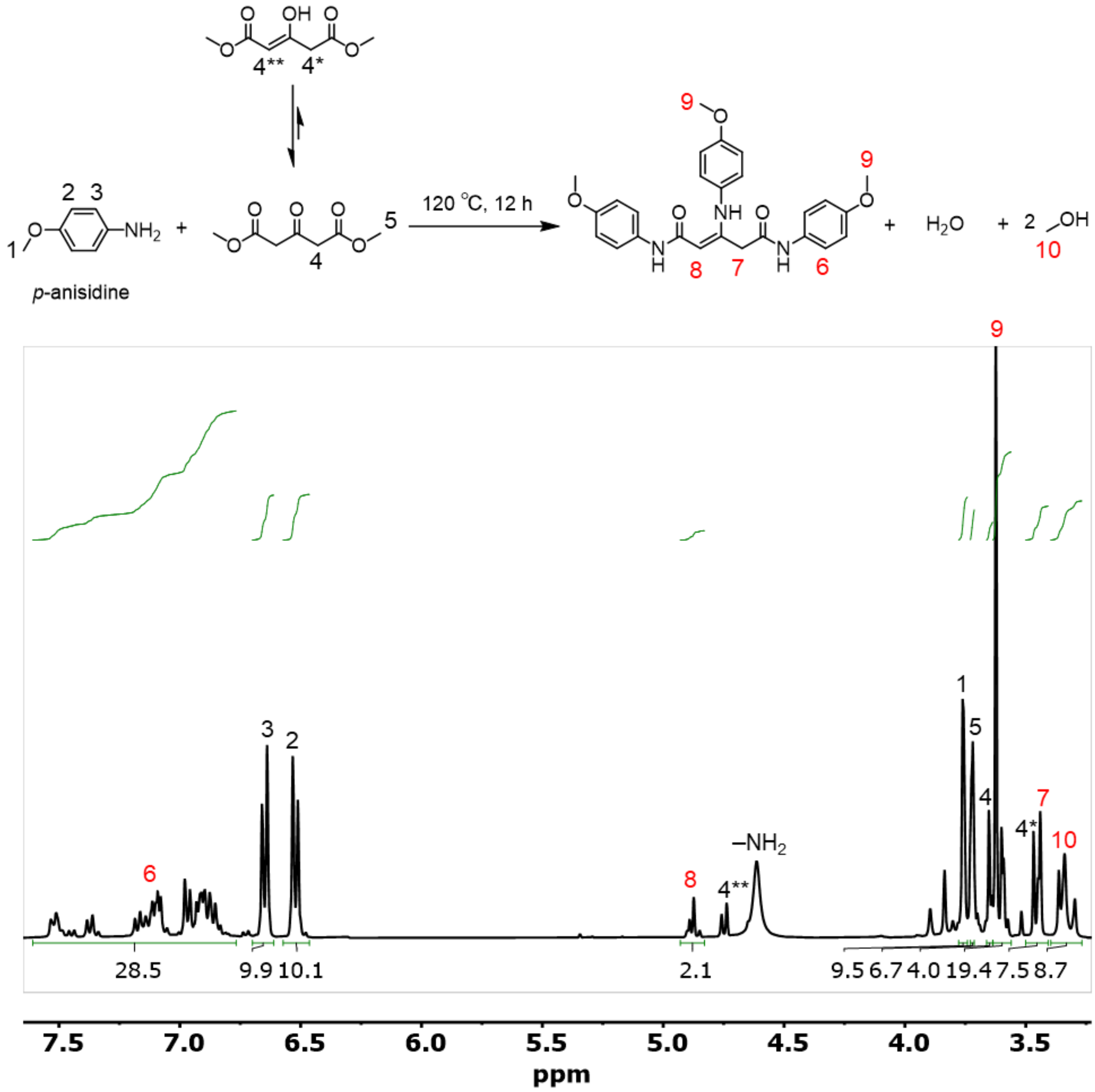

Figure S22. Scheme and ^{1}H NMR spectrum (DMSO-d_6, 400 MHz) of the reaction mixture of **DADC** and 3 eq. of *p*-anisidine upon thermal treatment at 120 °C for 12 h.

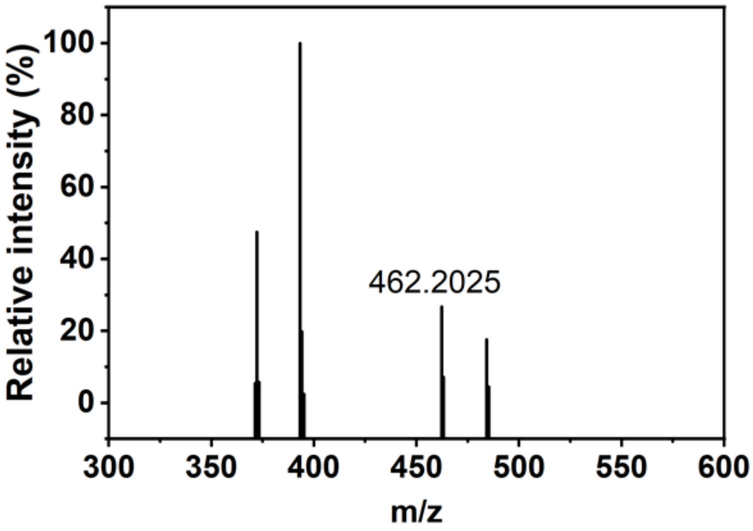

Figure S23. HRMS spectrum of the mixture of **DADC** and *p*-anisidine (3 eq.) upon thermal treatment at 120 °C for 12 h. HRMS (ESI/QTOF) m/z: $[M + H]^+$ Calcd for $C_{26}H_{28}N_3O_5^+$ 462.2023; Found 462.2025.

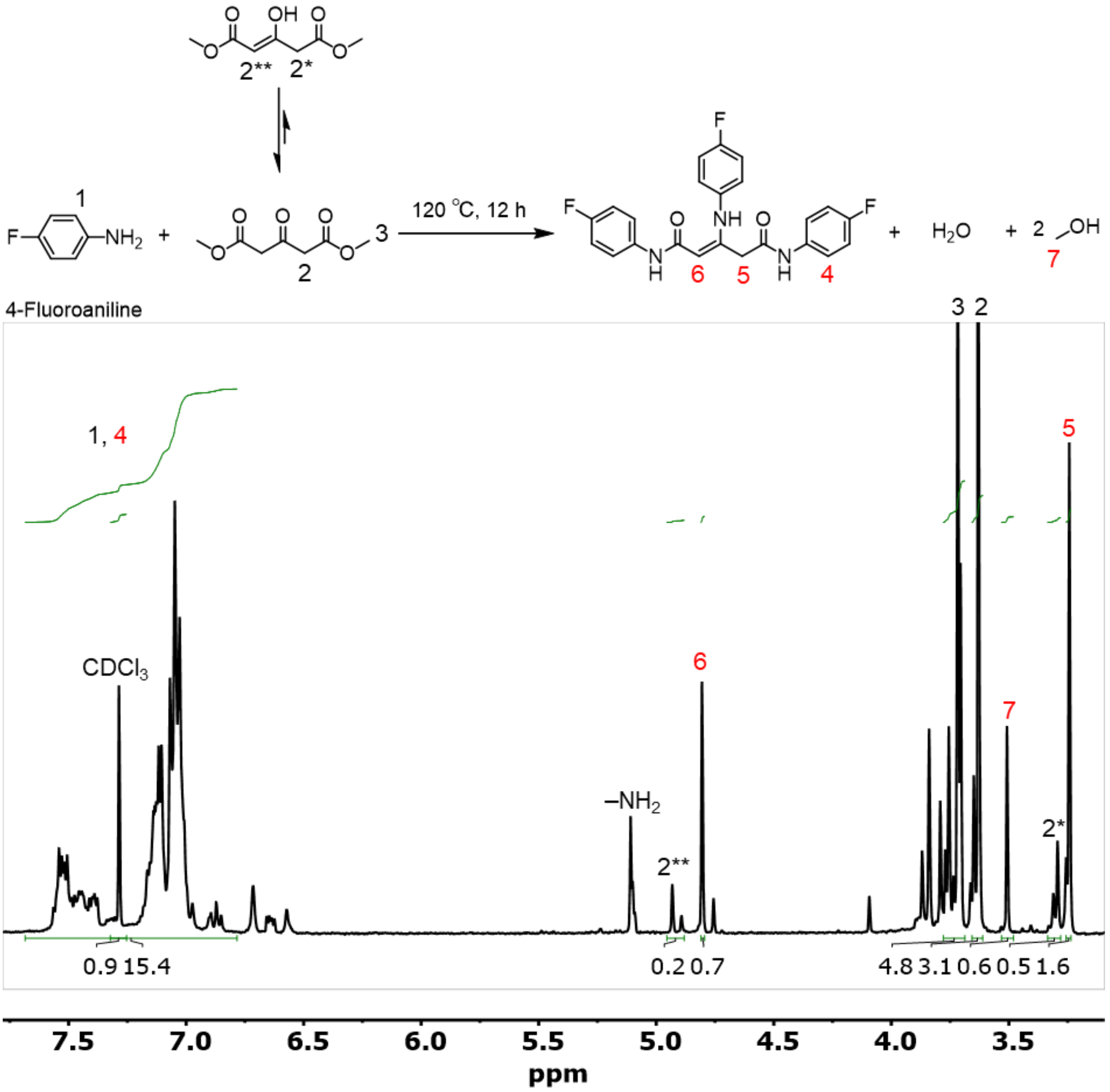

Figure S24. Scheme and ^{1}H NMR spectrum ($CDCl_3$, 400 MHz) of the reaction mixture of **DADC** and 3 eq. of 4-fluoroaniline upon thermal treatment at 120 °C for 12 h.

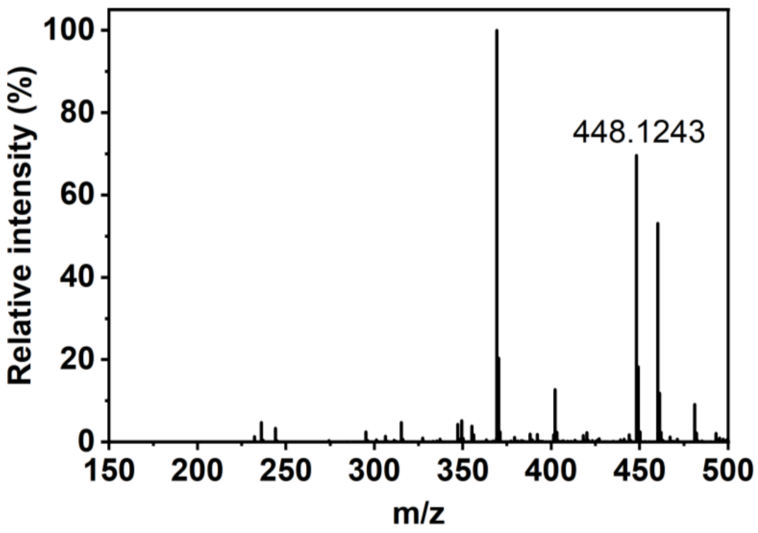

Figure S25. HRMS spectrum of the mixture of **DADC** and 4-fluoroaniline (3 eq.) upon thermal treatment at 120 ℃ for 12 h. HRMS (ESI/QTOF) m/z: $[M + Na]^+$ Calcd for $C_{23}H_{18}F_3N_3NaO^{2+}$ 448.1243; Found 448.1242.

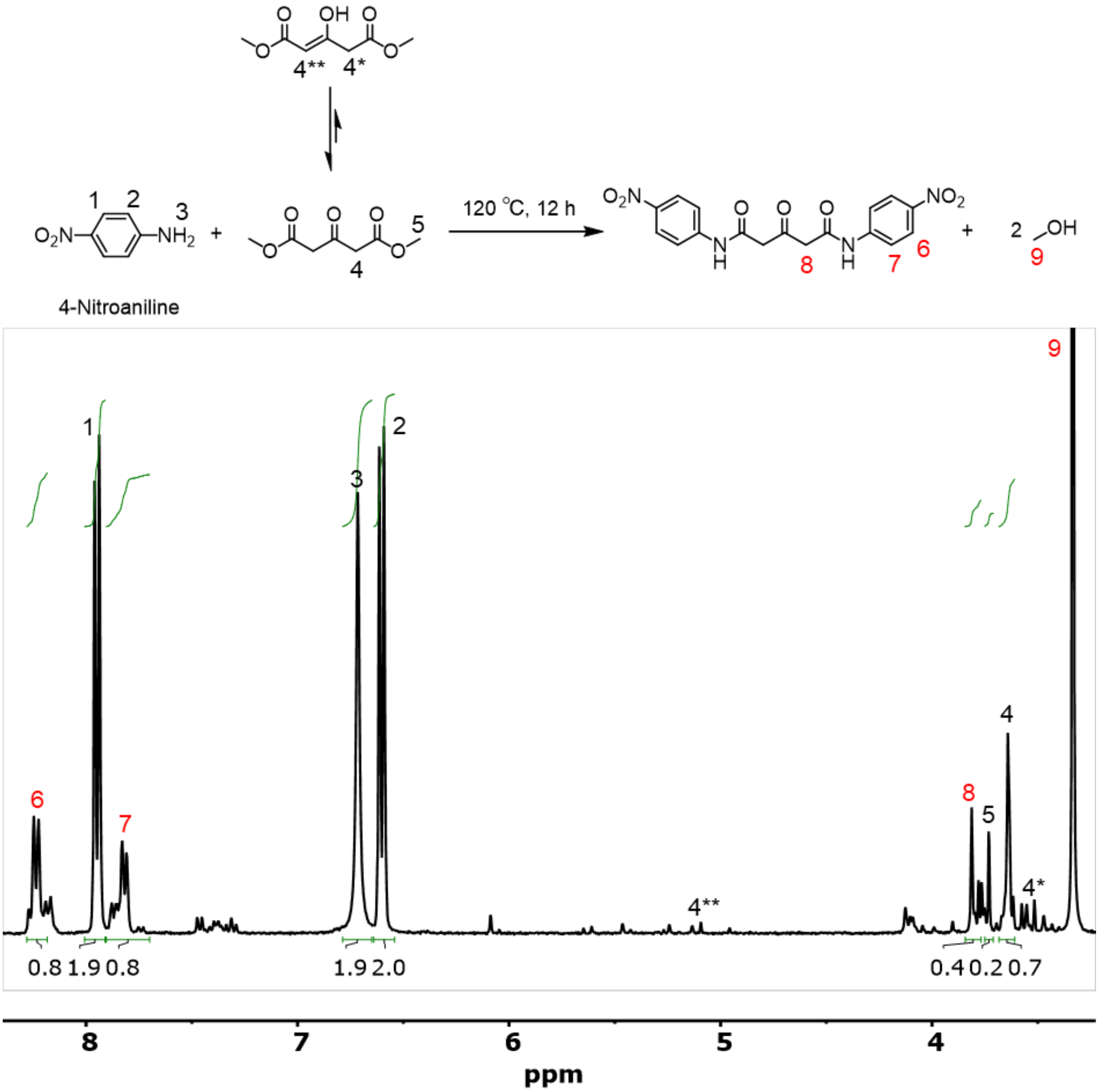

Figure S26. Scheme and ^{1}H NMR spectrum (DMSO-d_6, 400 MHz) of the reaction mixture of **DADC** and 3 eq. of 4-nitroaniline upon thermal treatment at 120 °C for 12 h.

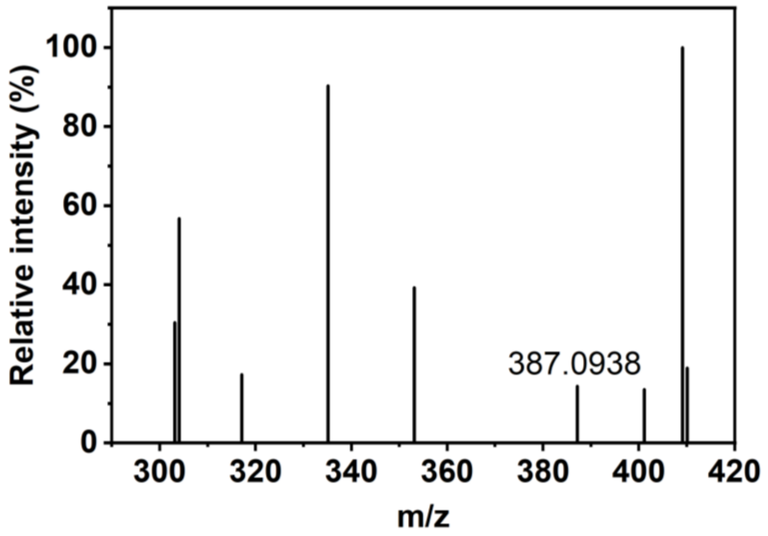

Figure S27. HRMS spectrum of the mixture of **DADC** and 4-nitroaniline (3 eq.) upon thermal treatment at 120 ℃ for 12 h. HRMS (ESI/QTOF) m/z: $[M + H]^+$ Calcd for $C_{17}H_{15}N_4O_7^+$ 387.0862; Found 387.0938.

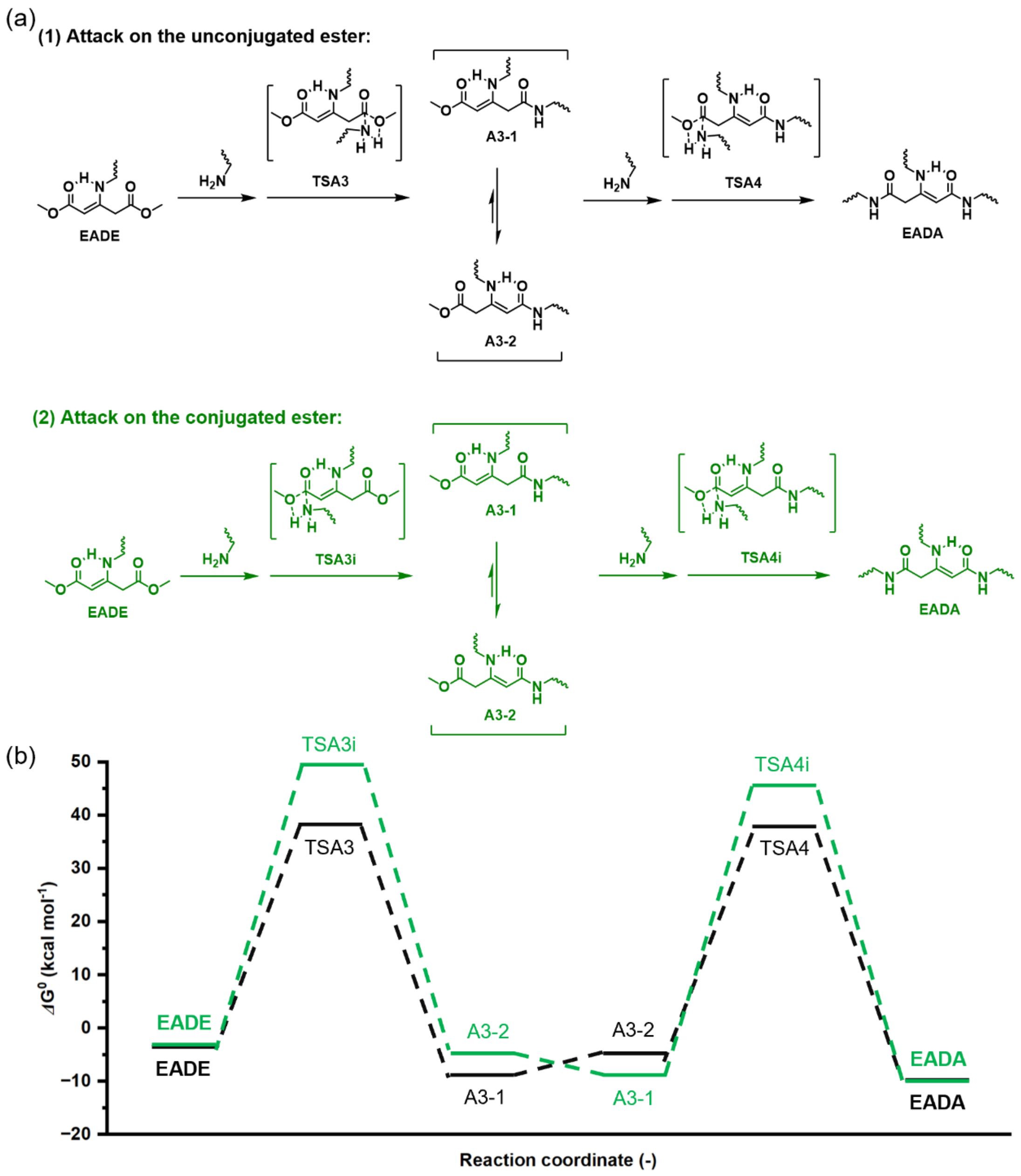

Figure S28. (a) Proposed mechanisms for the reaction of **EADE** and **Hea** including the attack of amines either on the (1) unconjugated or (2) conjugated ester linkages. (b) Gibbs free energies (ΔG^0) of the stationary points along the reaction paths in (a), with the pathways (1) and (2) depicted in black and green colors, respectively.

Synthesis of EADA-cross-linked thermosets

The incorporation of **EADA** linkages in the skeleton of an elastomeric thermoset was implemented by reacting poly(propylene glycol) bis(2-aminopropyl ether) (**PPG**) and **DADC** (reaction scheme shown in Figure S29a) according to the following procedures. In a 20 mL small vial with a magnetic stir bar, 6 g of **PPG** (3 mmol) and 0.8, 0.87, or 0.93 eq. of **DADC** were dissolved in 5 mL of DMF. The resulting solution was heated to 120 ℃ and kept for 4 h, and then poured into a Teflon mold. It is noteworthy that the reaction can be largely accelerated at higher temperatures. The reaction solution was also tried to be heated at 150 ℃, it transitioned into a gel within 3 h. After that, DMF was removed at 80 ℃ overnight in a drying oven (no vacuum), before it was fully cured in a vacuum oven at 120 °C for 24 h. A brownish film was obtained, which was labelled as **PEADA-*n***. Parameter **n** represents the molar feeding ratio of the sum of ketone and carboxylate groups of **DADC** to the amino groups of **PPG**. The specific amounts used for the preparation of **PEADA-*n*** library are reported in Table S2.

Table S2. Recipes for the synthesis of **PEADA-*n***.

Sample name	PPG		DADC	
	Weight (g)	mmol	Weight (g)	Mmol
**PEADA-1.2**	6	3	0.42	2.4
**PEADA-1.3**	6	3	0.45	2.6
**PEADA-1.4**	6	3	0.49	2.8

The difference in the reactivity of **PPG** toward the ketone and carboxylate moieties of **DADC** was investigated in a two-step approach. As shown in Figure S30(a) (left), 6 g of **PPG** (3 mmol) and **DADC** (0.42 g, 2.4 mmol) were added in a 20 mL small vial, and stirred for one week at room temperature. The mixture solution turned from transparent to yellowish, and yet maintained liquid state (Figure S30a, middle). A small amount of the mixture was taken out and sent for ^{1}H NMR analysis. Then, 5 mL of DMF was added to the solution and heated at 120 ℃ for 4 h. The reaction mixture was transferred into a Teflon mold and subjected to a drying oven at 80 ℃ overnight to remove DMF. Finally, the resulting solid was further cured in a vacuum oven at 120 °C for 24 h, and afforded a **PEADA-1.2** film (Figure S30a, right).

The incorporation of **EADA** linkages in the skeleton of a cross-linked plastic was implemented by reacting 1,12-dodecanediamine (**DDA**) and **DADC** (Figure S35a), following the same protocol as in the synthesis of **PEADA-*n***. In a 20 mL small vial with a magnetic stir bar, 2 g of **DDA** (10 mmol) and 0.8 eq. of **DADC** (1.39 g) were dissolved in 3 mL of DMF. The resulting solution was heated to 120 ℃

and kept for 4 h, and then poured into a Teflon mold. After that, DMF was removed at 80 °C overnight in a drying oven (no vacuum), before it was fully cured in a vacuum oven at 120 °C for 24 h. A black film was obtained, which was labelled as **PDDA**.

The cross-linking of polyallylamine (**PALA**) with **DADC** involves initial deprotonation of polyallylamine hydrochloride (**PALA•HCl**) into **PALA**, followed by the reaction of **PALA** and **DADC** upon thermal treatment (Figure S37a). The deprotonation of **PALA•HCl** was carried out as follows. First, 6 g of **PALA•HCl** (repeat unit in 93.5 g mol^{-1}) and 0.95 eq. of NaOH (2.44 g) per protonated amino group were added to 60 mL of methanol in a 150 mL single-necked flask. The mixture was reacted at room temperature for 4 h, before it was filtrated to remove the by-product NaCl. Methanol was removed by rotary evaporation, and the resulting crude was then vacuum dried at 60 °C overnight to afford 3.4 g of **PALA** (off-white solid, 93%).

PALA: ^1H NMR (400 MHz, D_2O) δ 2.51 (s, 2H), 1.44 (s, 1H), 1.13 (s, 2H). The ^1H NMR spectrum of **PALA** and its comparison with that of **PALA•HCl** are shown in Figure S36.

The **PALA** thus produced (1.14 g, 20 mmol repeat unit) and 5 mol% **DADC** to the amino group were dissolved in 5 mL methanol in a 20 mL small vial, and reacted at 60 °C for half an hour with magnetic stirring, prior to its solution casting in a Teflon mold. Upon the evaporation of methanol in the fume hood, the Teflon mold was put in a drying oven at 80 °C for 24 h to afford a brownish film, which was termed as **PALA-5%EADA**.

The cross-linking of gluten protein with **DADC** proceeds in the absence of any solvent and leverages the compression-molding technique, with the procedures shown below. First, 3 g of gluten extracted from wheat (≥ 75% protein content) was mixed with 0.9 g of glycerol and 0.15 g of **DADC** (5 wt% to gluten). The mixture was kneaded in an agate motar by a pestle to prepare a dough (Figure S38a). The dough was then placed between two sheets of Teflon, compressed between two plates of steel at a distance of 0.2 mm, applying a pressure of 40 kN for 20 min at 80 °C. The process afforded a pale yellowish and transparent film, which was subsequently heated at 80 °C for 24 h in a drying oven (Figure S38a). The resulting film was labelled as **Gluten-5%EADA**.

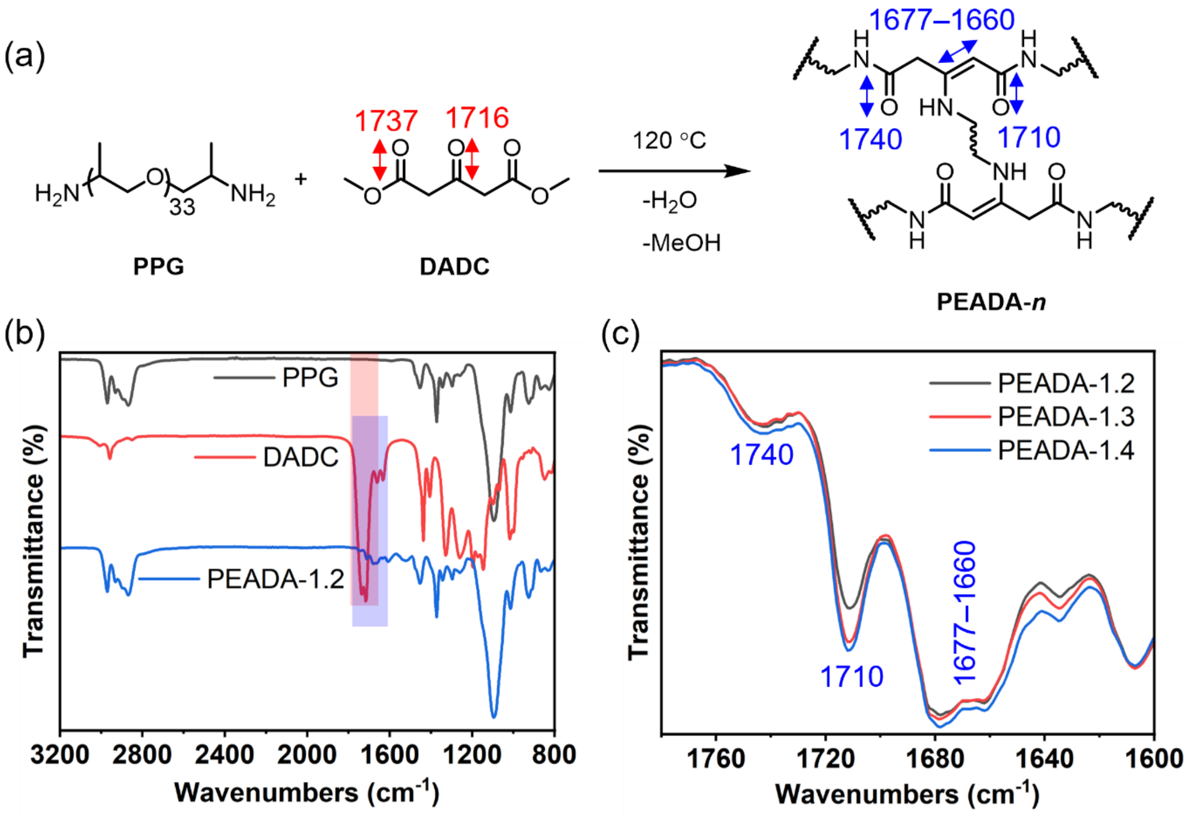

Figure S29. (a) Synthesis of **PEADA-*n*** from the polymerization of **PPG** and **DADC** at 120 °C, and the characteristic FTIR vibrations of **DADC** and the **EADA** linkages. (b) FTIR spectra showing the conversion of the starting materials **PPG** (black line) and **DADC** (red line) to **PEADA-1.2** (blue line), with the red area showing the spectrum region of carbonyl stretching vibrations of **DADC** (1716 cm^{-1}, 1737 cm^{-1}) and the blue area showing the spectrum region of carbonyl and vinyl stretching vibrations of **EADA** motif (1660–1677 cm^{-1}, 1710 cm^{-1}, 1740 cm^{-1}). (c) FTIR spectra in the region of 1780–1600 cm^{-1} of **PEADA-*n***, with **n** = 1.2 (black line), 1.3 (red line), and 1.4 (blue line).

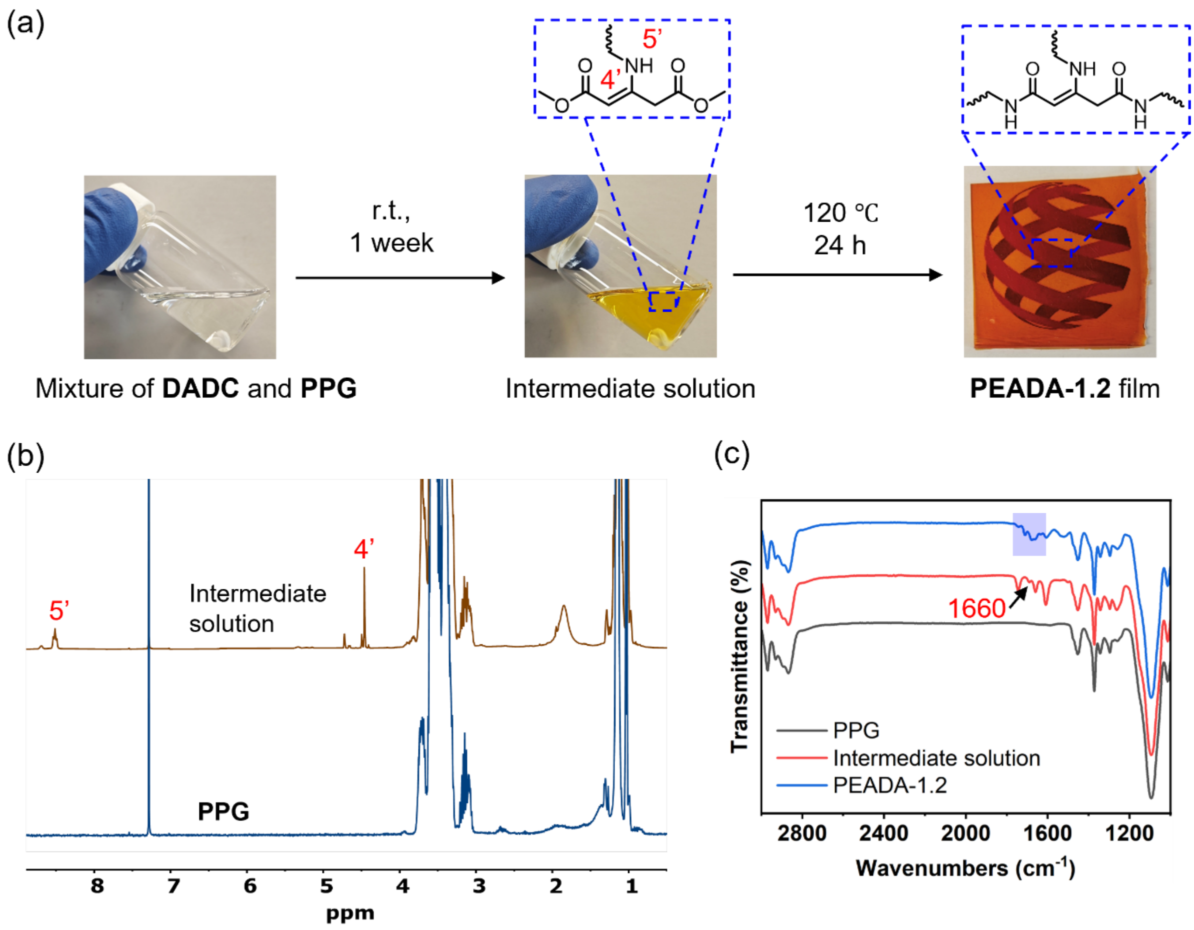

Figure S30. (a) Photographs showing the mixture of **PPG** and 0.8 eq. of **DADC** (left) first upon stirring at room temperature for one week (middle) for the formation of a yellowish intermediate solution, followed by the thermal treatment at 120 °C for 24 h to afford a **PEADA-1.2** film (right). (b) ^{1}H NMR spectra ($CDCl_3$, 400 MHz) of **PPG** and the intermediate solution, with the key protons assignment shown in the middle of (a). (c) FTIR spectra of **PPG**, the intermediate solution, and **PEADA-1.2** film.

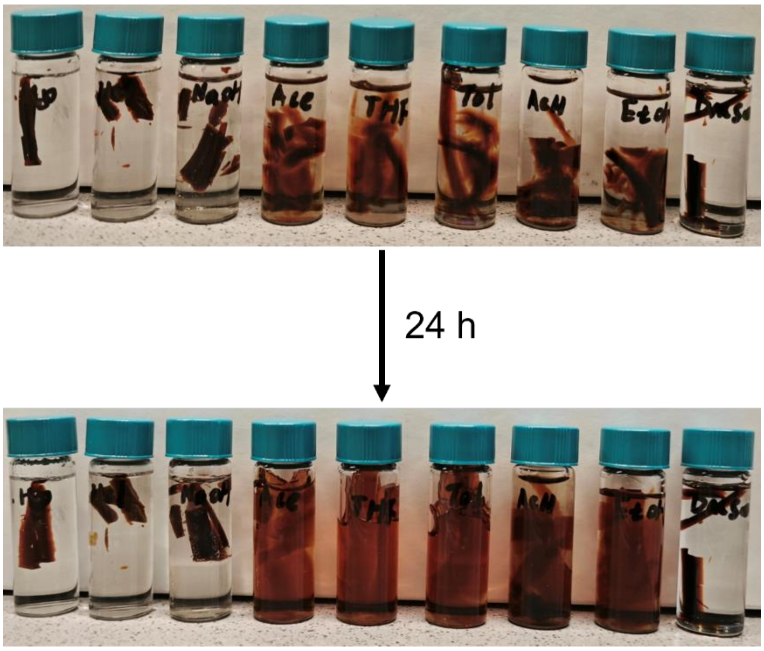

Figure S31. Photographs of **PEADA-1.2** dispersed in various solvents including H_2O, 1 M HCl, 1 M NaOH, acetone, tetrahydrofuran, toluene, acetonitrile, ethanol, and DMSO (from left to right) before (top row) and after (bottom row) a 24h-incubation period.

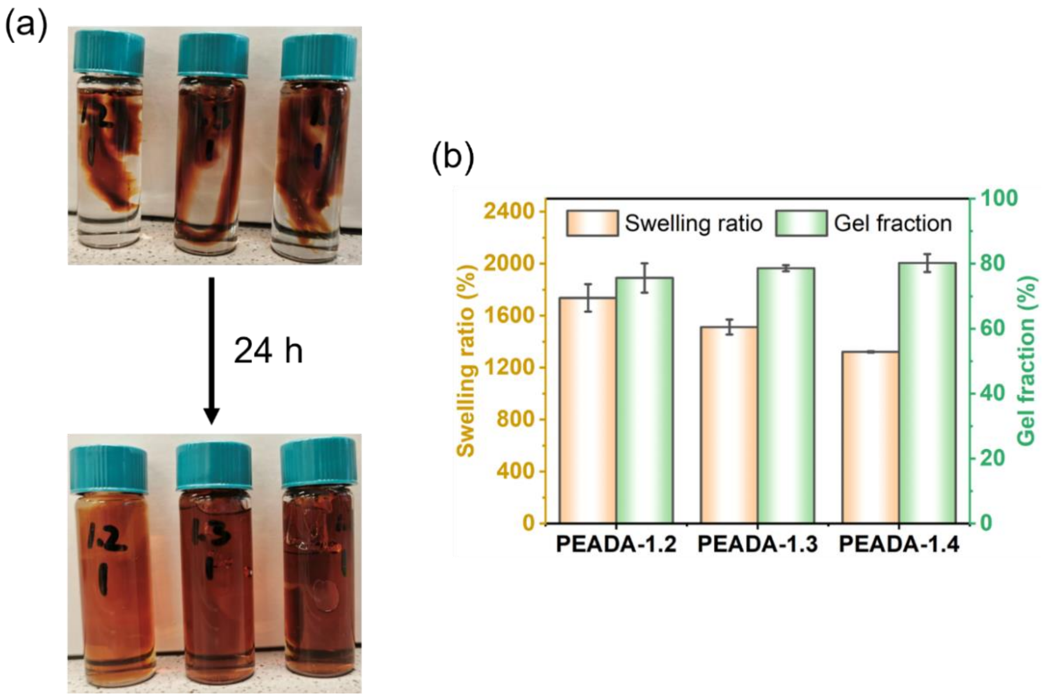

Figure S32. (a) Photographs of **PEADA-*n*** (**n** = 1.2, 1.3, and 1.4, from left to right) dispersed in DCM before (top) and after (bottom) a 24h-incubation period, with the solvent exchange every 6 h. (b) Swelling ratio (orange) and gel fraction (green) of **PEADA-*n***.

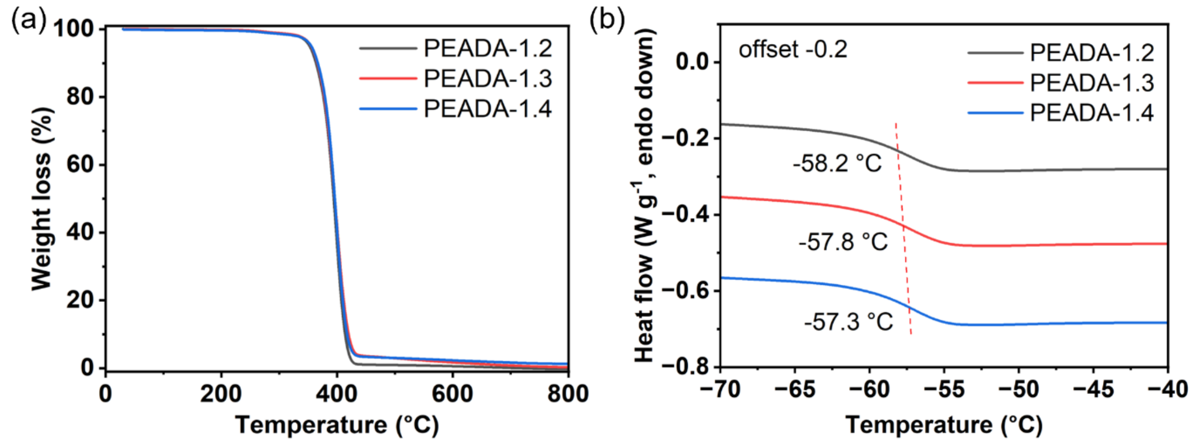

Figure S33. (a) TGA curves and (b) DSC traces of the second heating runs for **PEADA-*n***, with ***n*** = 1.2 (black), 1.3 (red), and 1.4 (blue).

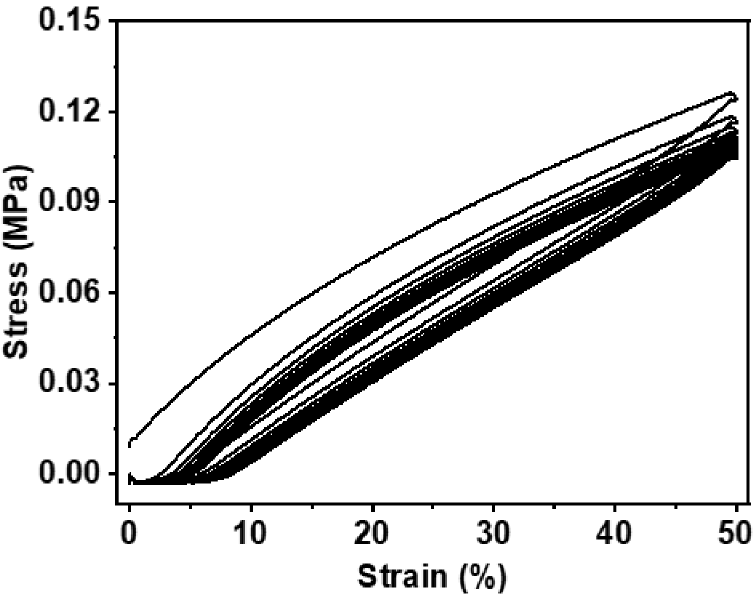

Figure S34. Cyclic tensile curves of **PEADA-1.4** at a maximum loading strain of 50% for 20 consecutive cycles.

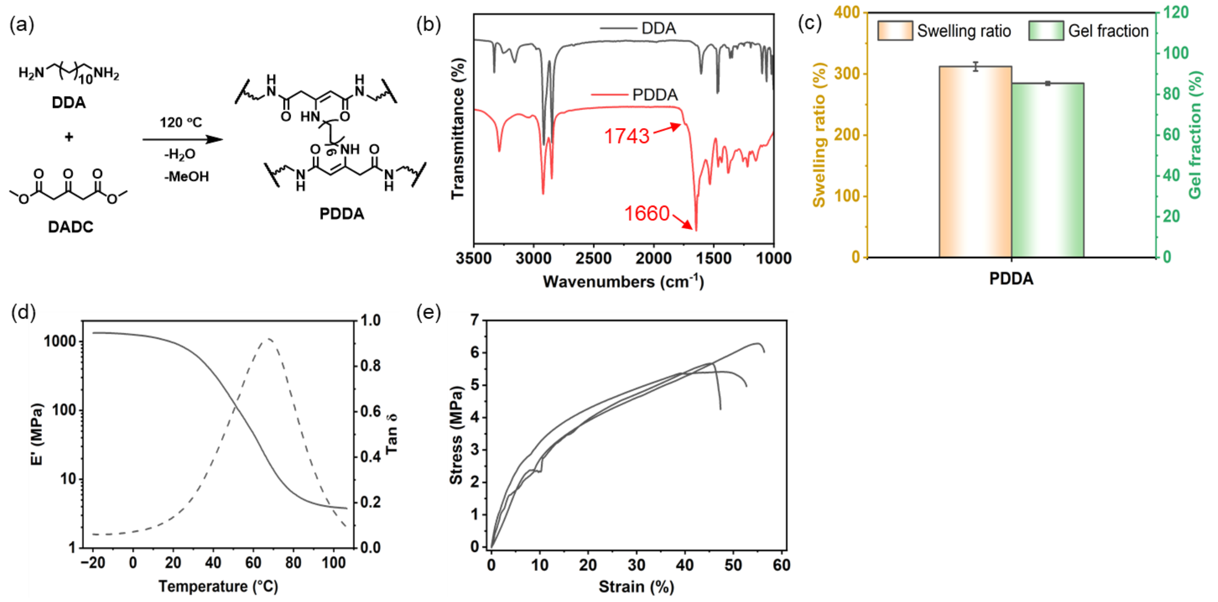

Figure S35. (a) Synthesis of poly(1,12-dodecanediamide)s **(PDDA)** from the polymerization of **DDA** and **DADC** at 120 °C. (b) FTIR spectra of **DDA** and **PDDA**. (c) Swelling ratio (orange) and gel fraction (green), (d) DMA traces showing the storage modulus (solid lines) and tan δ (dashed lines), and (e) stress–strain curves of **PDDA**.

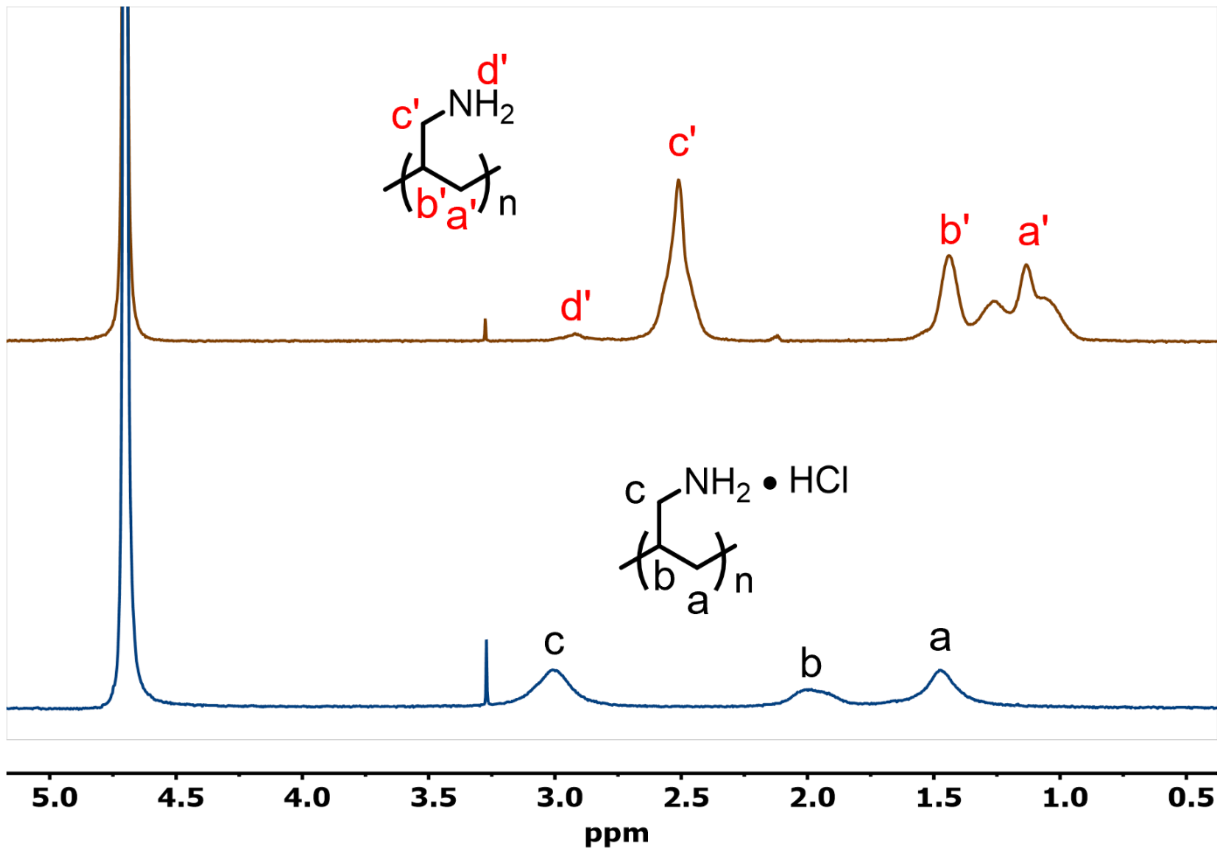

Figure S36. ^{1}H NMR (400 MHz, D_2O) spectra of **PALA•HCl** (bottom) and **PALA** (top).

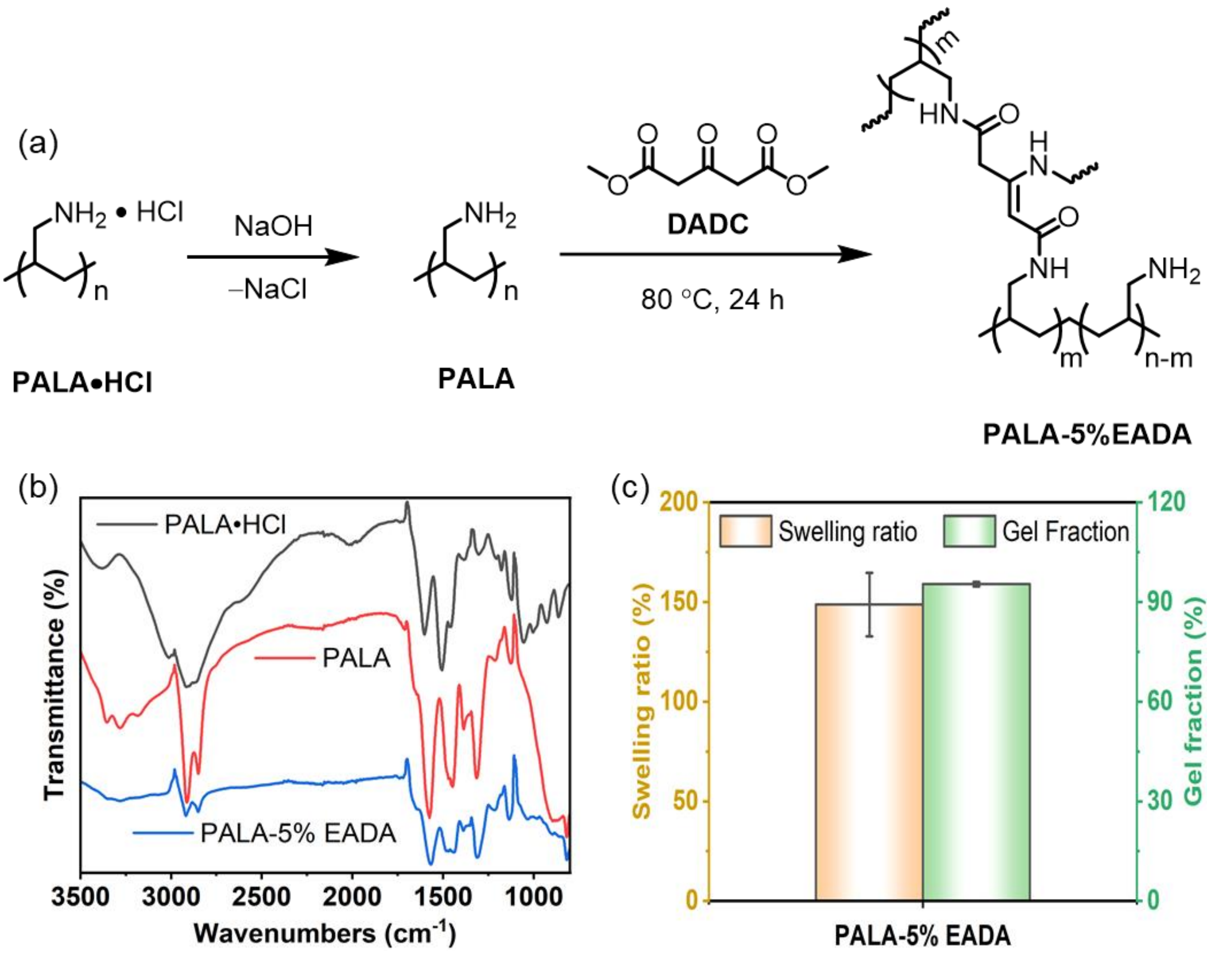

Figure S37. (a) Synthesis of **PALA-5%EADA** through the initial deprotonation of **PALA•HCl** using NaOH into **PALA**, followed by the cross-linking of **PALA** with 5 mol% **DADC** per amino group upon thermal treatment at 80 °C for 24 h. (b) FTIR spectra of **PALA•HCl** (black line), **PALA** (red line), and **PALA-5%EADA** (blue line). (c) Swelling ratio (orange) and gel fraction (green) of **PALA-5%EADA**.

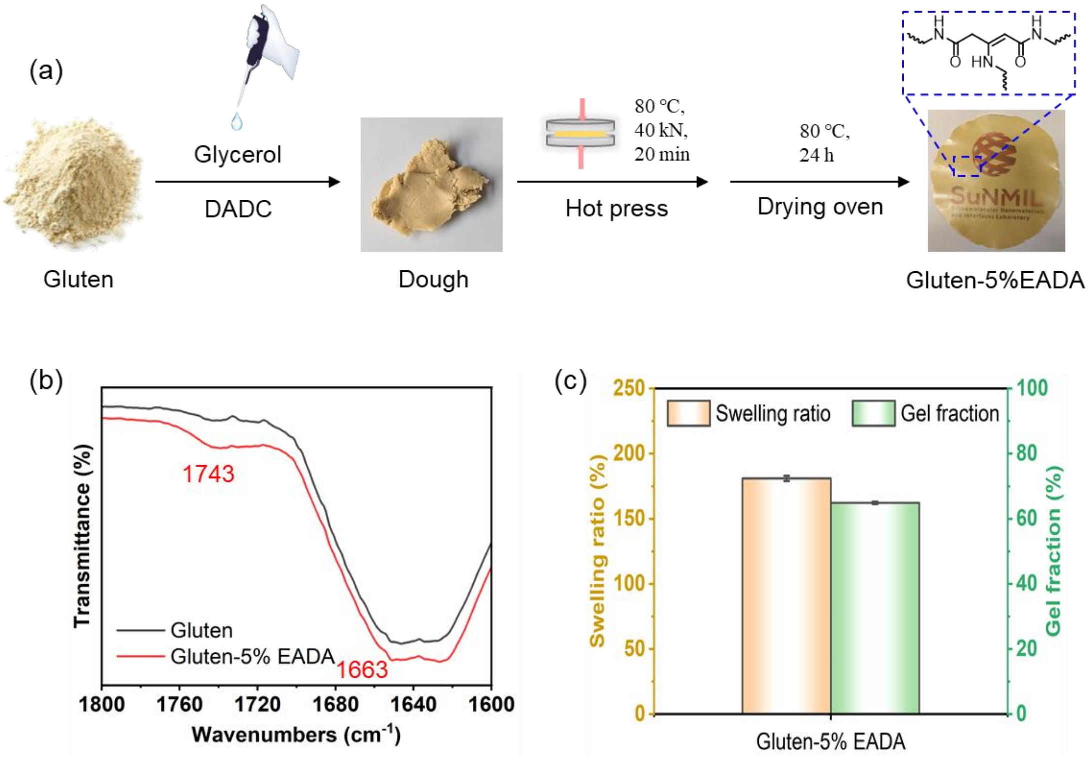

Figure S38. (a) Preparation of a **Gluten-5%EADA** film through first kneading the mixture of gluten, glycerol (30 wt%), and **DADC** (5 wt%) into a dough, followed by compression-molding the dough at 80 °C under a pressure of 40 kN for 20 min, and then curing the resulting film at 80 °C for 24 h. (b) FTIR spectra in the region of 1800–1600 cm^{-1} of gluten and **Gluten-5%EADA**. (c) Swelling ratio (orange) and gel fraction (green) of **Gluten-5%EADA**.

Table S3. Mechanical properties of **PEADA-*n***, PALA, **PALA-5%EADA**, Gluten, and **Gluten-5%EADA** films.

Sample name	*E'* at 25 °C (MPa)	Young's modulus	Stress at break	Strain at break
**PEADA-1.2**	0.076±0.001	0.114±0.005	0.079±0.007	158±3
**PEADA-1.3**	0.163±0.001	0.177±0.008	0.095±0.005	105±2
**PEADA-1.4**	0.243±0.000	0.31±0.013	0.154±0.013	78.0±4
**PDDA**	830±7	38.6±6.4	5.75±0.52	51.0±5
**PALA**	392±8	4.14±0.94	0.400±0.151	464±11
**PALA-5%EADA**	5370±12	320±58	14.8±0.9	4.36±1.44
**Gluten**	1220±8	258±39	12.7±0.4	11.2±1.9
**Gluten-5%EADA**	1290±6	268±61	13.3±0.2	20.0±1.6

Synthesis and hydrolysis of trihexyl-substituted EADA

The synthesis procedures of a **EADA** derivative are shown as follows. In a 50 mL single-necked flask with a magnetic stir bar, 3.48 g of **DADC** (20 mmol) was slowly added to 20.2 g of **Hea** (200 mmol) under nitrogen atmosphere. The mixture was heated to 120 °C and kept for 24 h. After the reaction, unreacted **Hea** was first removed by rotary evaporation, and the resulting crude was further purified by vacuum drying at 80 °C overnight to afford 7.7 g of trihexyl-substituted **EADA** (brownish solid, 97%).

Trihexyl-substituted **EADA**: ^{1}H NMR (400 MHz, DMSO-d_6) δ 9.20 – 6.60 (m, 3H), 4.42 (d, *J* = 9.0 Hz, 1H), 3.48 (d, *J* = 12.1 Hz, 2H), 3.26 – 2.97 (m, 4H), 2.96 – 2.73 (m, 2H), 1.64 – 1.01 (m, 24H), 0.86 (dd, *J* = 6.7, 3.5 Hz, 9H).

^{13}C NMR (101 MHz, DMSO-d_6) δ 169.02, 168.45, 49.85, 43.03, 42.90, 39.05, 31.46, 29.49, 26.69, 26.37, 22.54, 14.38.

The ^{1}H NMR, ^{13}C NMR, and HRMS spectra are shown in Figures S39, S40, and S41.

The hydrolysis of the trihexyl-substituted **EADA** proceeds in a mixture solution comprising 5 M NaOH and methanol-d_4 or ethanol-d_6 according to the following protocol. First, 0.2 g of NaOH was dissolved in 1 mL of D_2O to prepare a 5 M NaOH solution. Meanwhile, 50 mg of the **EADA** derivative was dissolved in 2 mL of methanol-d_4 or ethanol-d_6 solvent in a 4 mL small vial, together with a magnetic stir bar. Notably, the dissolution of **EADA** derivative in methanol-d_4 is slow due to the large polarity difference. After the dissolution, the 5 M NaOH solution was combined with the methanol-d_4 or ethanol-d_6 solution. The resulting mixture was heated to 70 °C, while being stired at a rate of 600 rpm. After different reaction time intervals, 0.5 mL aliquots of the mixture solution were taken out with a syringe and transferred to a NMR tube. ^{1}H NMR spectra of these solutions comprising of methanol-d_4 or ethanol-d_6 were measured and shown in Figure S42 or S43, respectively.

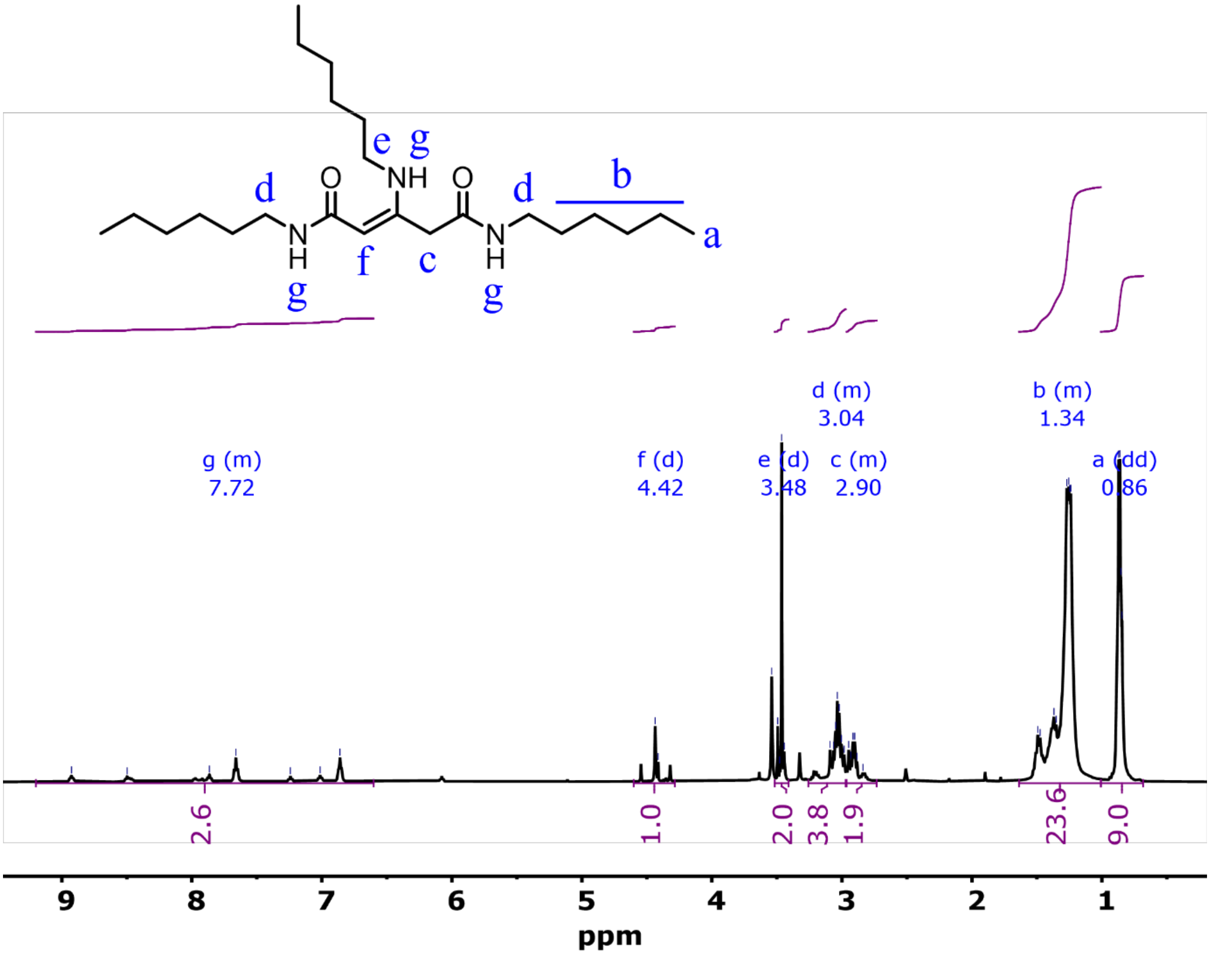

Figure S39. ^{1}H NMR (400 MHz, DMSO-d_6) spectrum of trihexyl-substituted **EADA**.

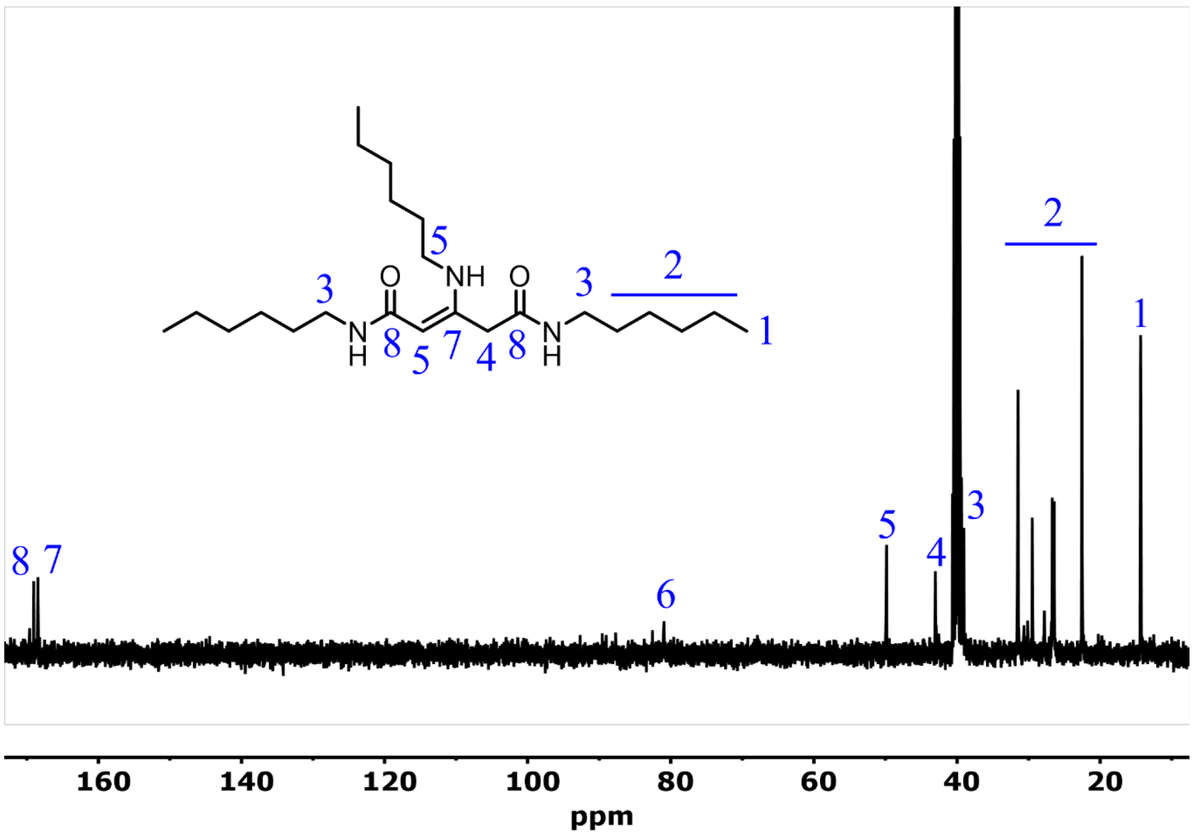

Figure S40. ^{13}C NMR (101 MHz, DMSO-d_6) spectrum of trihexyl-substituted **EADA**.

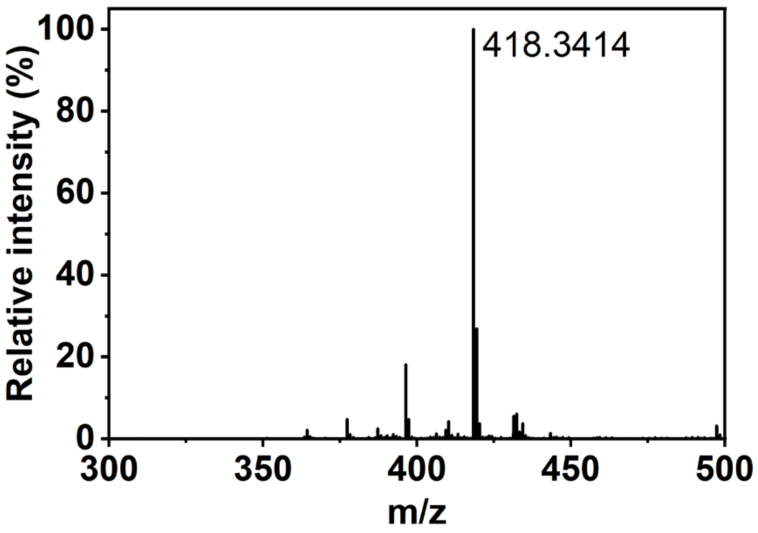

Figure S41. HRMS spectrum of trihexyl-substituted **EADA**. HRMS (ESI/QTOF) m/z: $[M + Na]^+$ Calcd for $C_{23}H_{45}N_3NaO_2^+$ 418.3404; Found 418.3414.

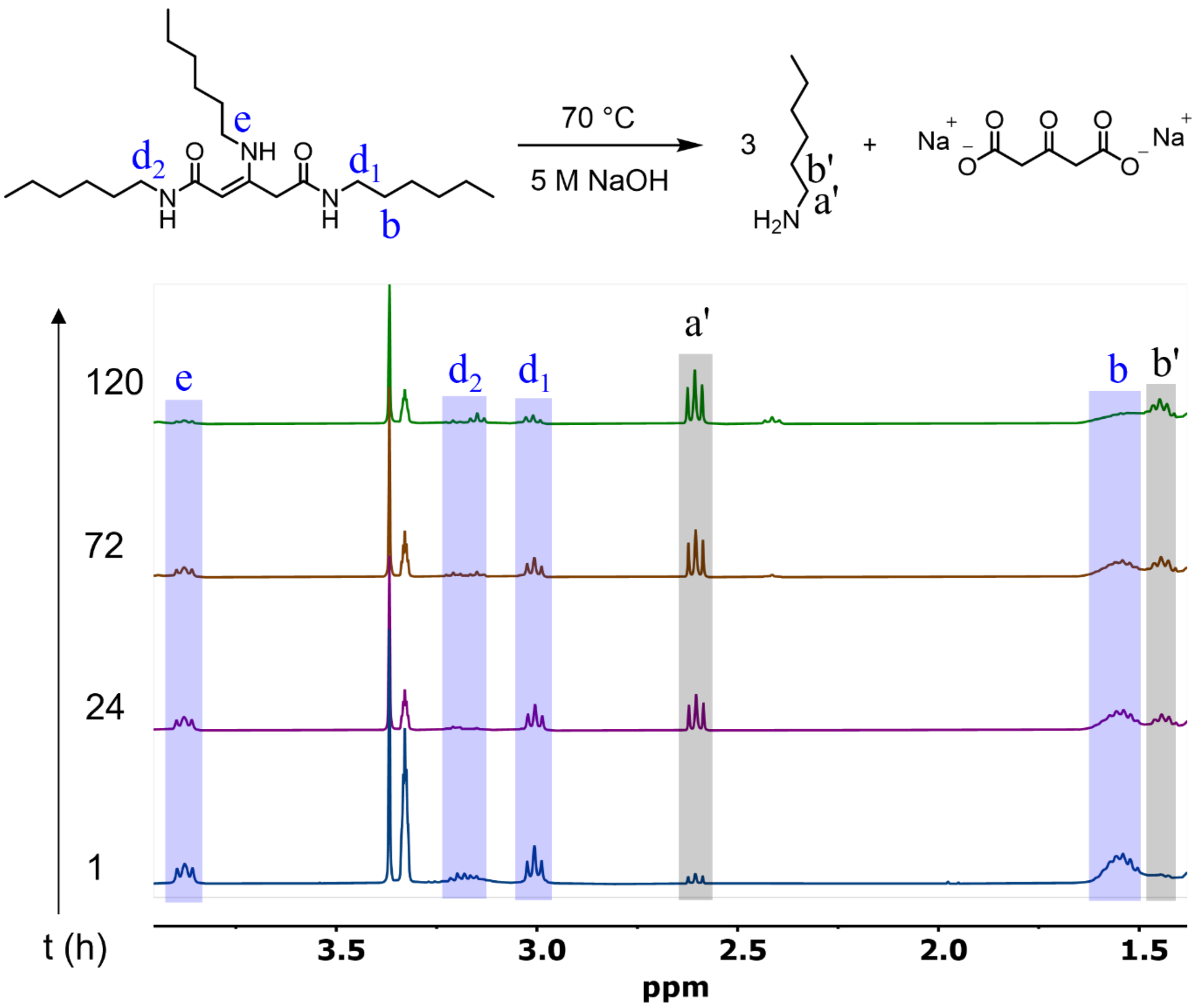

Figure S42. Scheme showing the hydrolysis of trihexyl-substituted **EADA** (50 mg) in 3 mL mixture solution comprising methanol-d_4 and 5 M NaOH (2/1, v/v) upon thermal treatment at 70 °C, and the evolution of the ^{1}H NMR signals (methanol-d_4, 400 MHz) during the hydrolysis reaction at different times (as indicated). The key protons assignment is given, with the blue areas highlighting the characteristic signals of the **EADA** derivative and the black areas for the hydrolyzed **Hea**.

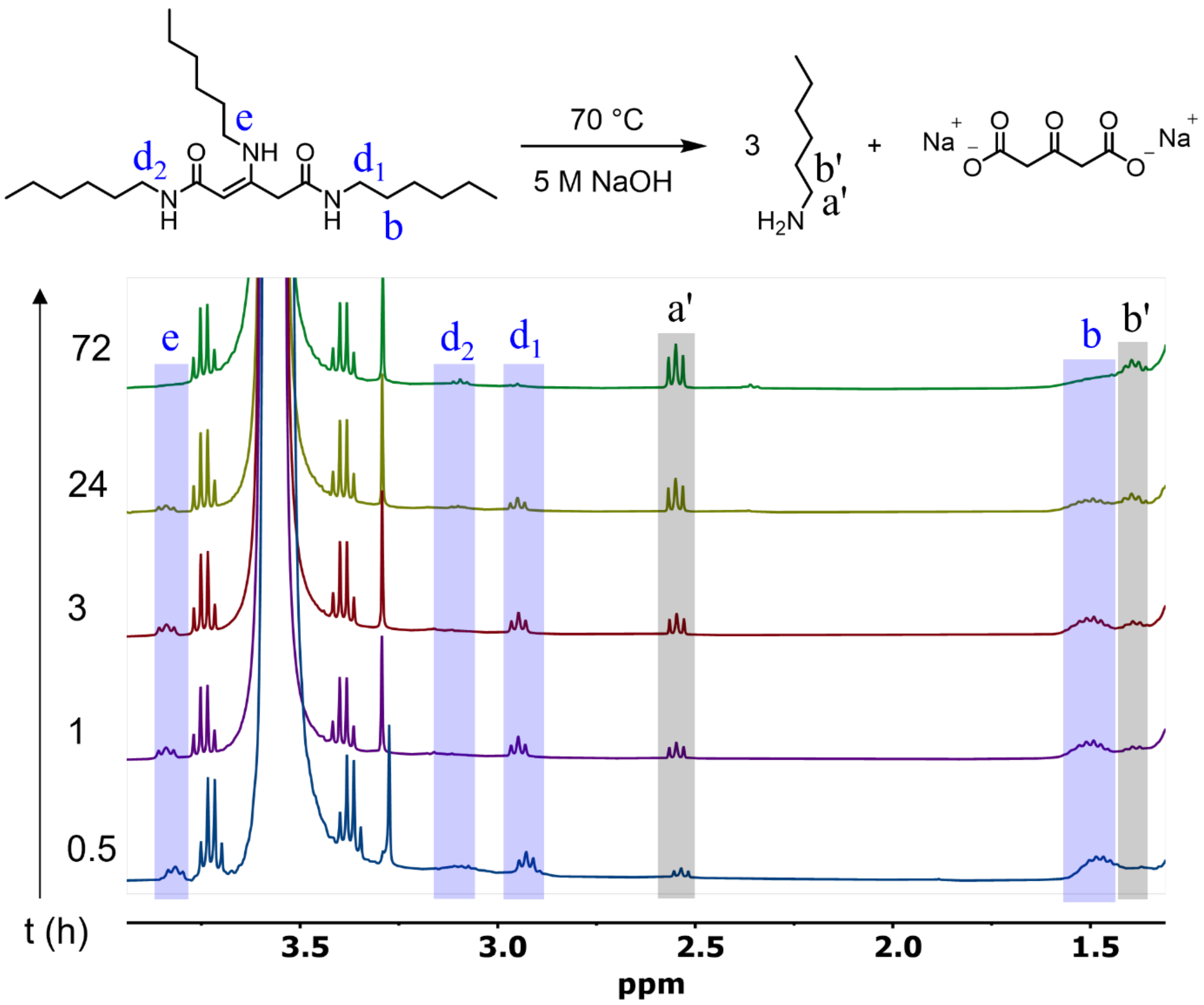

Figure S43. Scheme showing the hydrolysis of trihexyl-substituted **EADA** (50 mg) in 3 mL mixture solution comprising ethanol-d_6 and 5 M NaOH (2/1, v/v) upon thermal treatment at 70 °C, and the evolution of the ^{1}H NMR signals (ethanol-d_6, 400 MHz) during the hydrolysis reaction at different times (as indicated). The key protons assignment is given, with the blue areas highlighting the characteristic signals of the **EADA** derivative and the black areas for the hydrolyzed **Hea**.

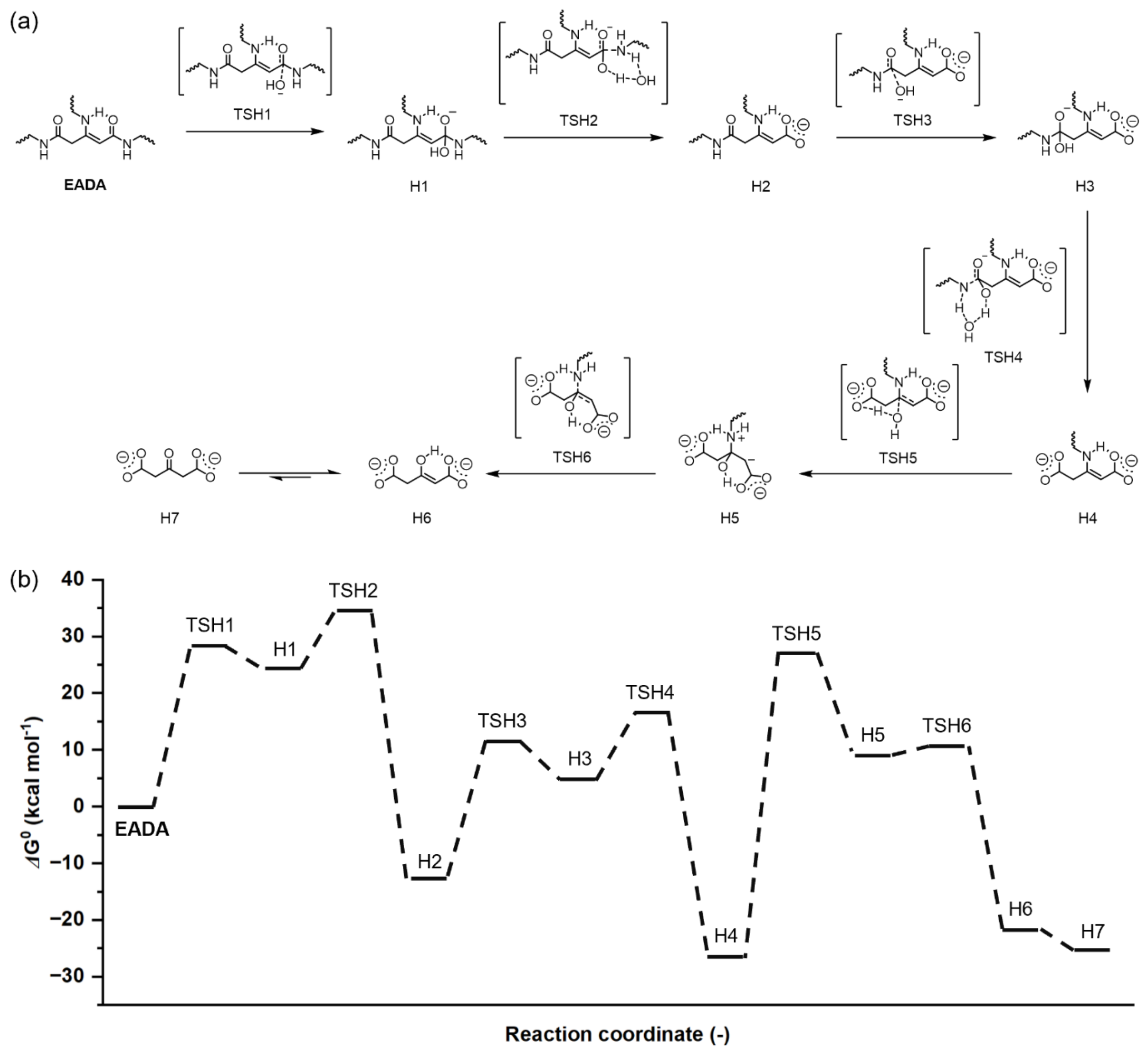

Figure S44. (a) Proposed mechanism for the hydrolysis reaction of trihexyl-substituted **EADA** into **Hea** and 3-oxopentanedioate (H7). (b) Gibbs free energies (ΔG^0) of the stationary points along the reaction path in (a).

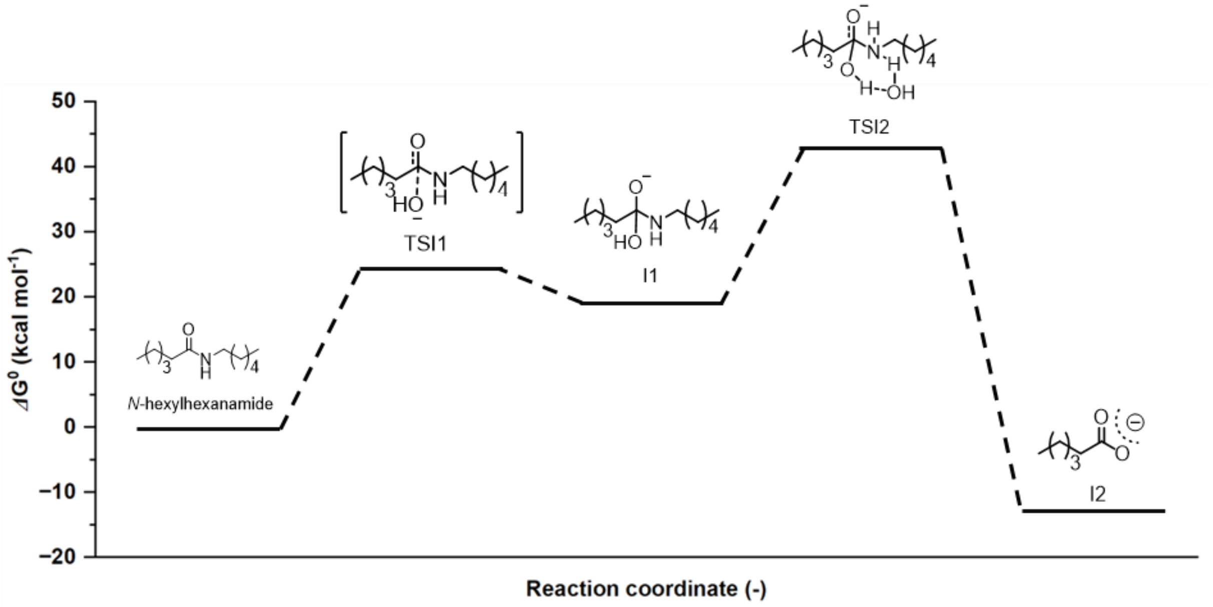

Figure S45. Proposed mechanism for the hydrolysis reaction of *N*-hexylhexanamide into **Hea** and hexyl carboxylate (I2), and the calculated ΔG^0 of the stationary points along the reaction path.

Depolymerization of PEADA-1.2 and its mixture with commercial plastics

The depolymerization of **PEADA-1.2** by alkali was carried out according to the following procedures. 0.5 g of **PEADA-1.2** small pieces collected from the tensile testing measurements was added to 15 mL mixture solution comprising ethanol and 5 M NaOH (2:1, v/v) in a 20 mL small vial. The resulting mixture was heated to 70 °C and kept for 3 days, while stirring at a rate of 600 rpm. It afforded a brownish solution, which was subsequently subjected to rotary evaporation for the removal of ethanol. Upon the evaporation of ethanol, a viscous liquid compound (off-yellowish) gradually appeared and separated away from the water phase. Then, ca. 20 mL DCM or diethyl ether was added to extract the viscous compound from the mixture, and washed with deionized water three times prior to the solvent removal using rotary evaporation again. After vacuum drying at 60 °C for 24 h, 416 mg of **PPG** was obtained in a yield of 89%.

The depolymerization and subsequent recovery of **PPG** from the mixture of **PEADA-1.**2 with several commercial plastics including polyethylene (plastic bag), polypropylene (Eppendorf microtube), polyurethane foam, poly(ethylene terephthalate) and nylon-6,6 granules (from Sigma-Aldrich) followed a similar protocol as in the recycling process of neat **PEADA-1.2** polymer. As shown in Figure S46(a), 0.5 g of **PEADA-1.2** small pieces were mixed with those commercial plastics before 15 mL ethanol–5 M NaOH mixture solution (2:1, v/v) was added. The mixture was heated at 70 °C for one day, and **PEADA-1.2** dissolved in the solution due to the partial depolymerization, while other plastics remained intact. Upon vacuum filtration, the commercial plastics were separated from the depolymerized polymer solution. The polymer solution was heated at 70 °C again for another two days prior to the extraction with DCM, washing with water, and vacuum drying treatments, as in the recycling of neat **PEADA-1.2**, to recover 423 mg of **PPG** (yield 91%).

The depolymerization of **PEADA-1.2** in acidic solution was conducted following the next procedures. 0.5 g of **PEADA-1.2** small pieces was added to 15 mL $CHCl_3$ comprising 0.1 mL trifluoroacetic acid (TFA) in a 20 mL small vial. The resulting mixture was heated to 60 °C and kept for 1 day, while being stirred at a rate of 500 rpm. It afforded a brownish solution. The solution was then washed by ca. 30 mL of 10 w/v% $NaHCO_3$ using a separation funnel, and the washing process was repeated for two times to remove TFA as much as possible. The washed solution was casted in a Teflon mold and dried in the fume hood for the removal of $CHCl_3$ before it was sent to vaccum drying at 120 °C for 24 h. The process regenerated a brownish film.

To determine the chemical structure of the acid-depolymerized solution, a parallel experiment was conducted. 20 mg of **PEADA-1.2** film and 10 μL TFA were added to 1 mL of $CDCl_3$ in a 2 mL small vial. The resulting mixture was heated to 60 °C and kept for 1 day. It afforded a brownish solution, which was sent for ^1H NMR analysis. The ^1H NMR spectrum was shown in Figure S48(c).

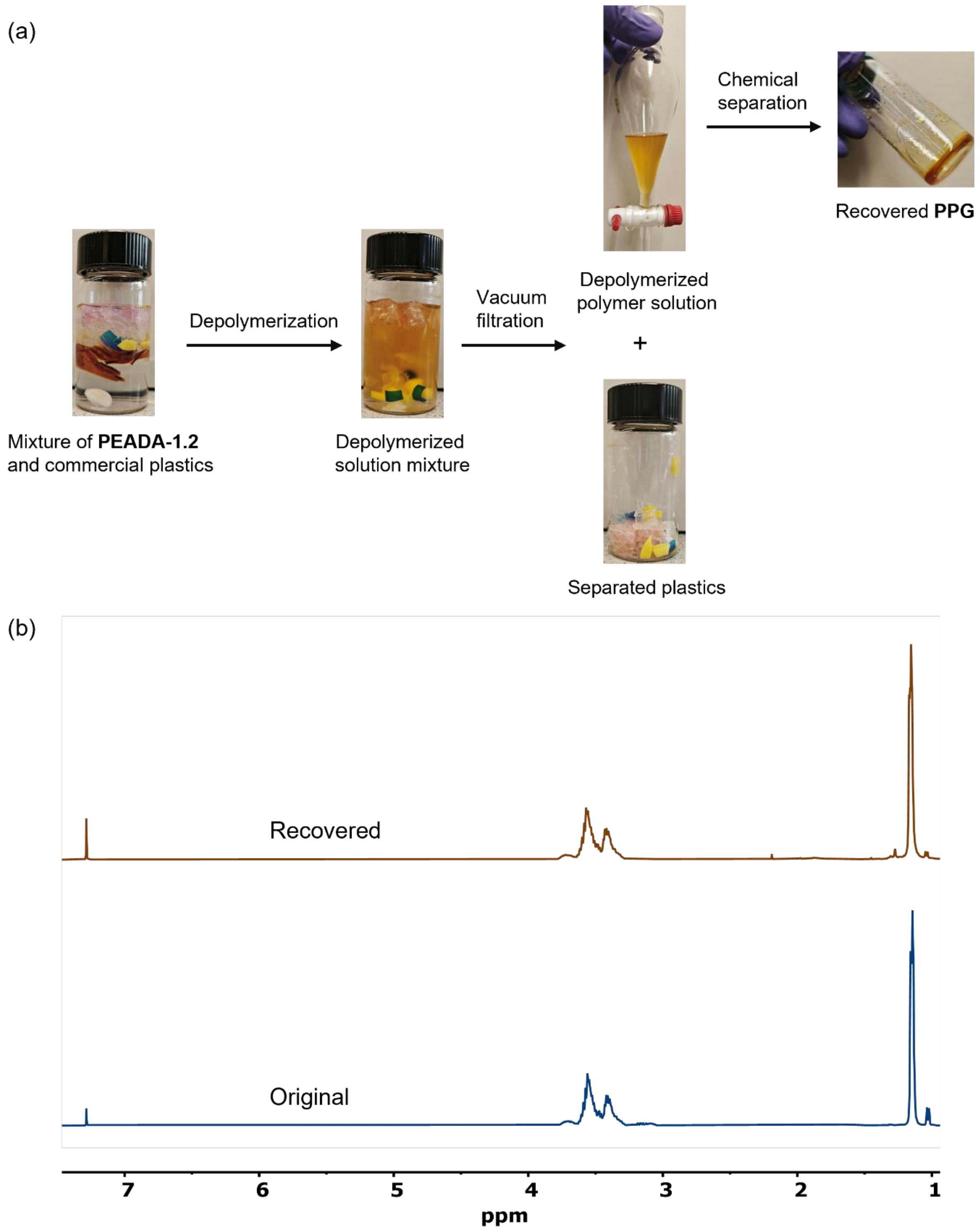

Figure S46. (a) Photographs showing the depolymerization of the mixture of **PEADA-1.2** (0.5 g) with several commercial plastics in the presence of 15 mL ethanol–5 M NaOH mixture solution (2:1, v/v), the separation of the depolymerized solution away from the commercial plastics by vacuum filtration, the subsequent chemical separation treatments on the solution to recover **PPG**. (b) Comparison between the ^{1}H NMR spectra ($CDCl_3$, 400 MHz) of the original and the recovered **PPG** obtained from the depolymerization of the mixture of **PEADA-1.2** and other commercial plastics.

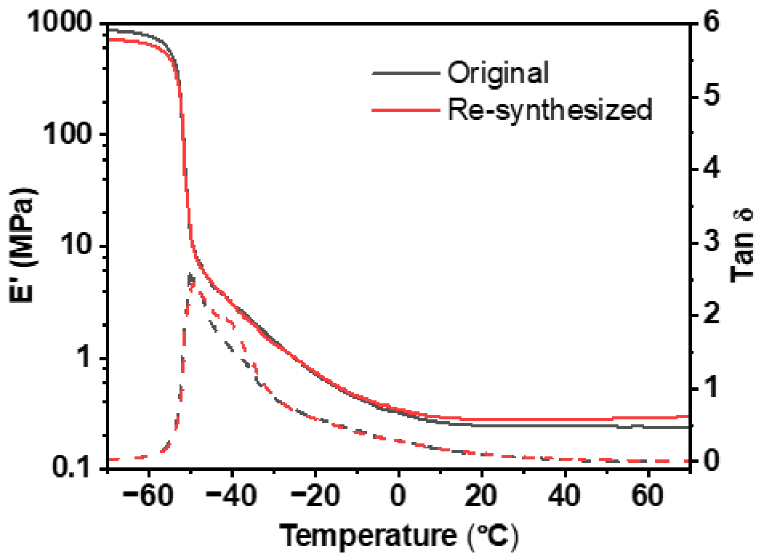

Figure S47. DMA traces showing the storage modulus (solid line) and tan δ (dashed line) as a function of temperature of the original (black) and re-synthesized (red) **PEADA-1.4**.

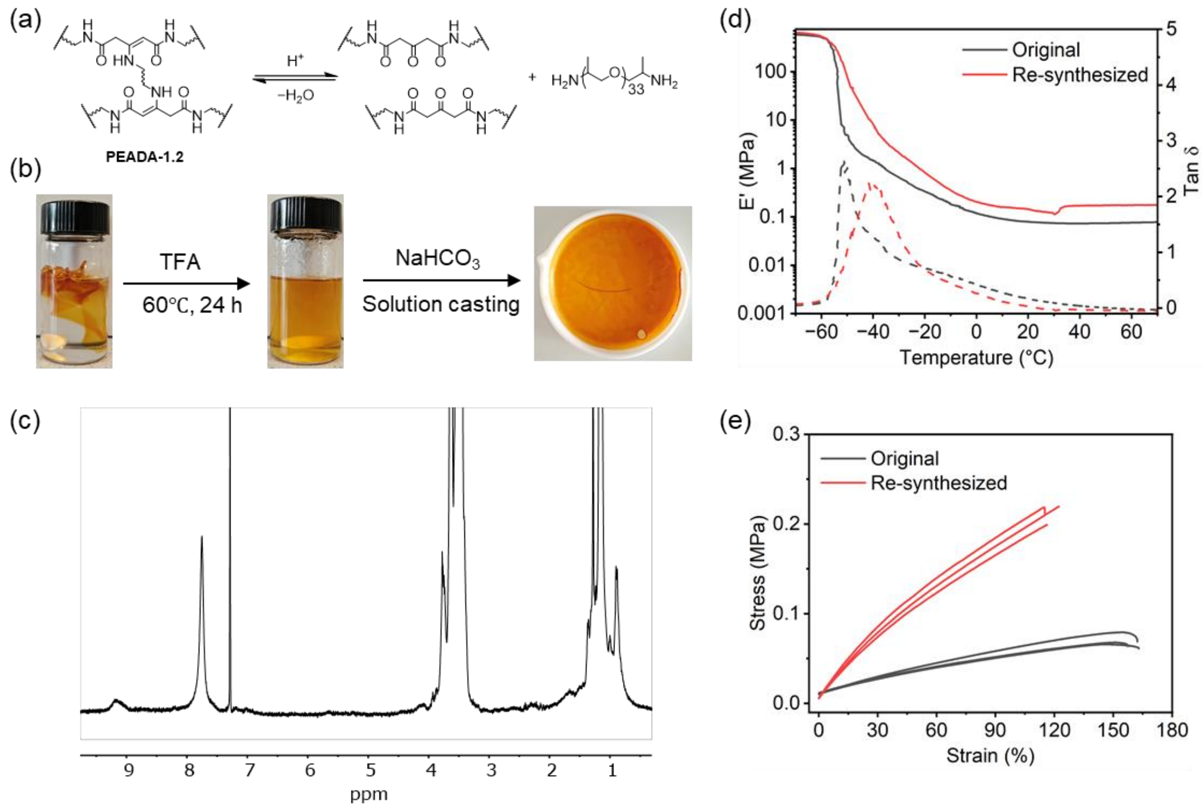

Figure S48. (a) Depolymerization of **PEADA-1.2** through the acid-catalyzed hydrolysis of imine linkages, followed by the repolymerization into **PEADA-1.2** upon sequential basic and thermal treatments. (b) Photographs showing the depolymerization of **PEADA-1.2** debris (left) by $CHCl_3$ comprising trifluoroacetic acid (TFA) at 60 °C into a brownish solution (middle), followed by the washing of the depolymerized solution with 10 w/v% $NaHCO_3$ and solution casting to reproduce a **PEADA-1.2** film. (c) ^1H NMR spectrum ($CDCl_3$, 400 MHz) of the depolymerized **PEADA-1.2** solution. (d) DMA traces showing the storage modulus (solid line) and tan δ (dashed line) and (e) stress–strain curves of the original (black) and re-synthesized (red) **PEADA-1.2**.

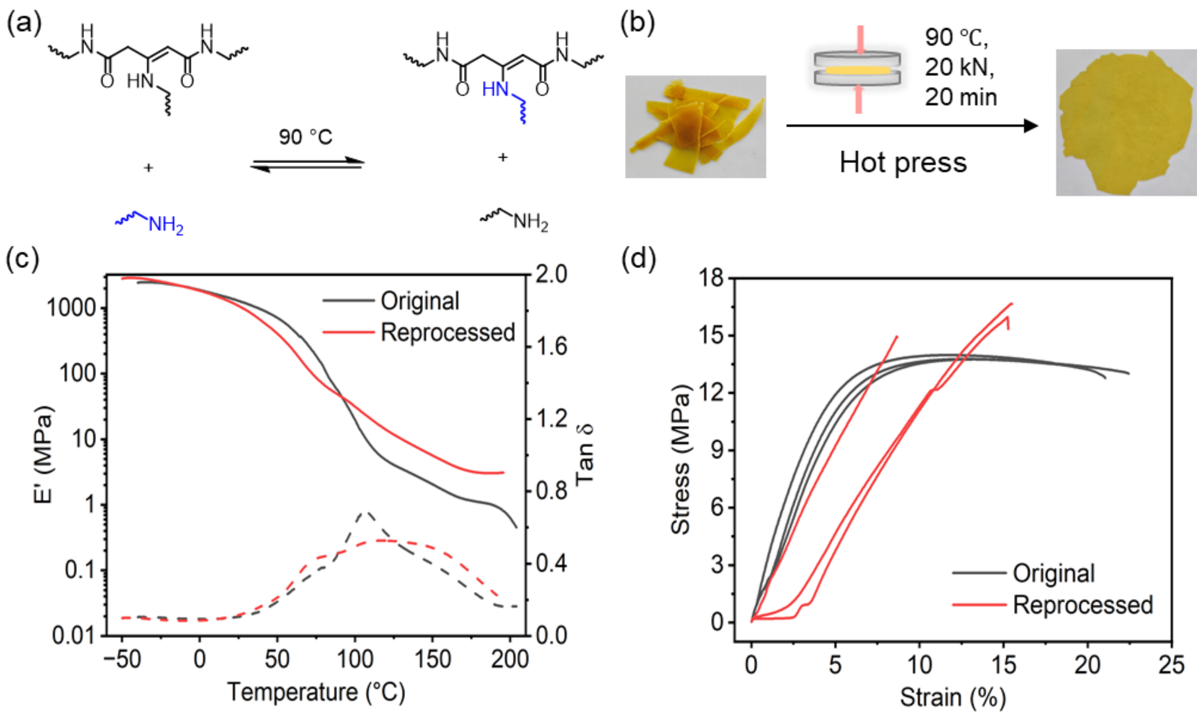

Figure S49. (a) The transamination reaction between an amine and an **EADA** linkage during thermal reprocessing. (b) Thermal reprocessing of **Gluten-5%EADA** from small pieces of material (left) into a new film (right) by hot press at 90 °C and a pressure of 20 kN for 20 min. (c) DMA traces showing the storage modulus (solid line) and tan δ (dashed line) and (d) stress–strain curves of the original (black) and reprocessed (red) **PEADA-1.2**.

Appendix

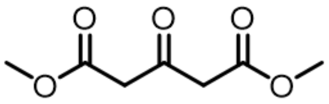

DADC

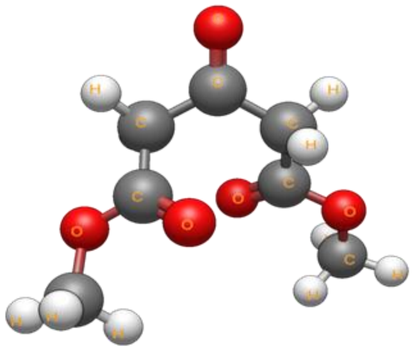

O	2.57570	-0.67865	0.43968
C	1.52789	-0.09616	-0.14756
C	1.17497	1.19112	0.55446
C	-0.00449	1.95294	-0.02591
C	-1.22256	1.21084	-0.55040
O	0.97032	-0.55373	-1.11112
C	-1.57501	-0.05745	0.18416
O	-2.47024	-0.77066	-0.50115
O	-1.14425	-0.38888	1.25835
C	3.00155	-1.91362	-0.14349
C	-2.88482	-1.99302	0.11461
O	0.02684	3.15549	-0.07187
H	2.04103	1.85448	0.56022
H	0.93888	0.94188	1.59293
H	-1.04357	0.95788	-1.59801
H	-2.07275	1.89538	-0.51528
H	3.29048	-1.75995	-1.18309
H	2.19611	-2.64654	-0.09879
H	3.85170	-2.23970	0.44883
H	-3.60833	-2.43356	-0.56543
H	-3.33788	-1.79273	1.08550
H	-2.02790	-2.65351	0.24682

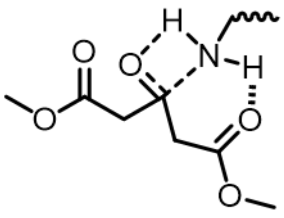

TSA1

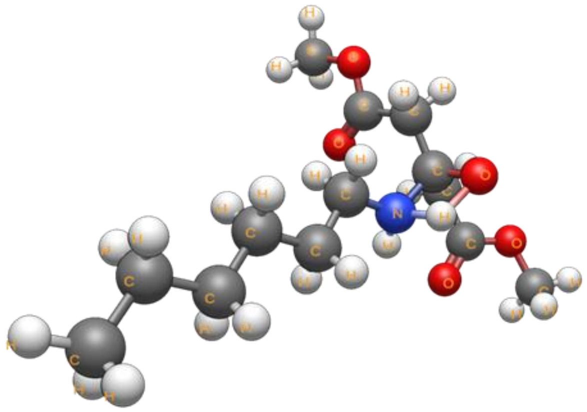

O	1.44777	3.74739	0.29876
C	1.29448	2.46923	-0.06846
C	1.53203	1.53611	1.09134
C	1.62499	0.04558	0.76016
C	2.75295	-0.20666	-0.26798
O	0.98891	2.14358	-1.18813
C	2.89462	-1.68089	-0.53373
O	4.06003	-2.17428	-0.12358
O	2.04465	-2.35307	-1.07041
C	1.21954	4.71770	-0.72645
C	4.22504	-3.58402	-0.29233
O	1.72143	-0.73486	1.86672
H	0.75157	1.70002	1.83813
H	2.46589	1.81585	1.58443
H	3.67803	0.17345	0.16529
H	2.54841	0.29826	-1.21409
H	0.19736	4.64253	-1.09788
H	1.91200	4.56311	-1.55390
H	1.38824	5.68399	-0.25948
H	5.21490	-3.81187	0.09294
H	4.14807	-3.85090	-1.34650
H	3.45987	-4.11750	0.27128
N	0.29575	-0.56384	0.23877
C	-0.97956	0.15516	0.37234
H	-0.98473	1.03074	-0.28053
H	-2.04036	-1.12133	-0.99651
H	-2.10128	-1.65098	0.68045
C	-2.14437	-0.76711	0.03557
C	-3.49137	-0.06775	0.20184
C	-4.67295	-0.97279	-0.13806
H	-3.59230	0.28678	1.23446
H	-3.52146	0.82269	-0.43718
C	-6.02369	-0.28009	0.02764
H	-4.57278	-1.32853	-1.17052
H	-4.64263	-1.86449	0.49938
C	-7.19612	-1.19561	-0.31531
H	-6.12152	0.07476	1.05925
H	-6.05152	0.61046	-0.60947
H	-7.20094	-2.07889	0.32852
H	-8.15327	-0.68560	-0.19175

H	-7.13051	-1.54013	-1.35057
H	-1.06204	0.48694	1.41054
H	0.62502	-1.21329	1.19351
H	0.39988	-1.02244	-0.66744

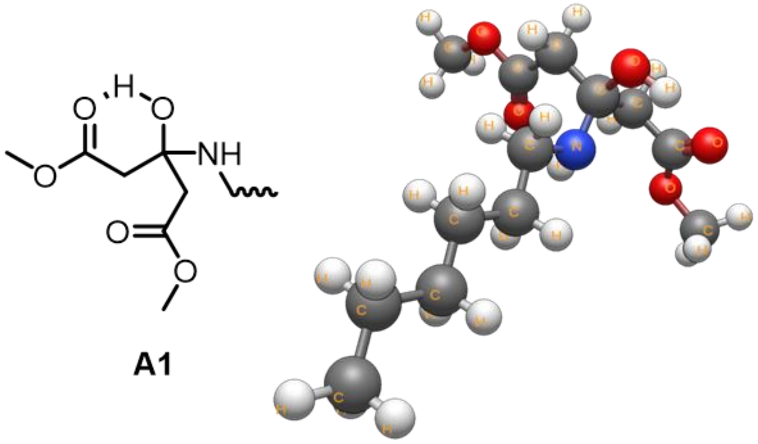

A1

O	1.88013	3.61824	0.05689
C	1.79165	2.30895	-0.20919
C	1.85548	1.49805	1.05896
C	1.64006	-0.01143	0.87604
C	2.83197	-0.65291	0.13785
O	1.67835	1.87786	-1.32895
C	2.57821	-2.13190	-0.01124
O	2.54648	-2.52390	-1.28570
O	2.40890	-2.88698	0.91541
C	1.83617	4.48409	-1.08005
C	2.24131	-3.90569	-1.49402
O	1.55495	-0.49320	2.19545
H	1.10243	1.88274	1.75086
H	2.82119	1.67903	1.53836
H	3.72770	-0.51849	0.74775
H	2.97266	-0.19279	-0.83919
H	0.89428	4.35867	-1.61427
H	2.66205	4.26295	-1.75625
H	1.92240	5.49242	-0.68498
H	2.25513	-4.05070	-2.57061
H	1.25739	-4.13439	-1.08452
H	2.98746	-4.53479	-1.00879
N	0.43713	-0.38055	0.14152
C	-0.80125	0.23453	0.61117
H	-0.83079	1.31987	0.41637
H	-1.91003	-0.33234	-1.14115
H	-1.99523	-1.48810	0.18020
C	-2.00666	-0.41868	-0.05189
C	-3.32776	0.20335	0.39213
C	-4.54339	-0.45355	-0.25707
H	-3.41584	0.12868	1.48273
H	-3.32706	1.27426	0.15530
C	-5.86806	0.16610	0.18238
H	-4.45514	-0.38251	-1.34808

H	-4.54792	-1.52392	-0.01830
C	-7.07418	-0.50191	-0.47315
H	-5.95368	0.09534	1.27207
H	-5.86203	1.23489	-0.05766
H	-7.11284	-1.56535	-0.22312
H	-8.01210	-0.04709	-0.14859
H	-7.02026	-0.41934	-1.56185
H	-0.85190	0.09627	1.69438
H	1.58565	-1.45999	2.15394
H	0.57484	-0.16145	-0.84153

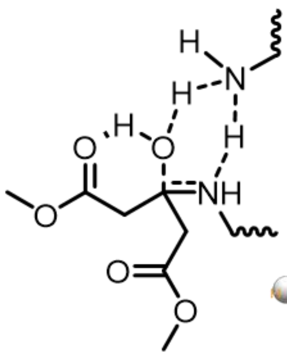

TSA2

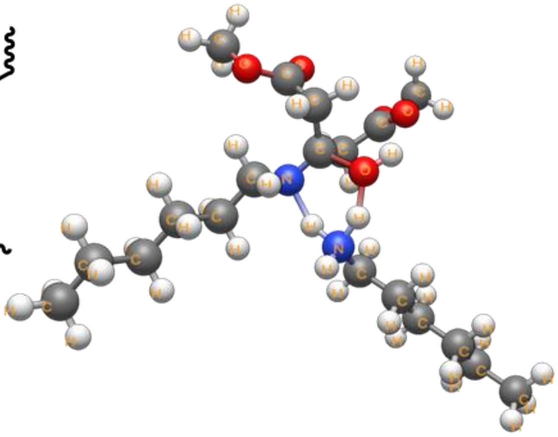

O	3.93457	-2.31361	0.40076
C	2.69032	-2.80204	0.29366
C	1.69484	-2.07693	1.16264
C	0.74432	-1.25944	0.27906
C	-0.10515	-2.08086	-0.68760
O	2.40944	-3.72232	-0.43210
C	-0.74924	-3.33878	-0.16151
O	-1.01523	-4.18764	-1.15895
O	-1.03778	-3.58077	0.98638
C	4.91187	-2.94394	-0.42878
C	-1.61856	-5.42328	-0.77343
O	-0.46820	-0.87555	1.57681
H	2.19172	-1.42573	1.87625
H	1.12554	-2.83175	1.70248
H	0.51348	-2.35849	-1.54110
H	-0.90013	-1.43726	-1.07462
H	4.63985	-2.83697	-1.47906
H	4.98727	-4.00430	-0.18674
H	5.84907	-2.43476	-0.22048
H	-1.75893	-5.98181	-1.69483
H	-2.57456	-5.24391	-0.28073
H	-0.96063	-5.96380	-0.09300
N	1.13565	-0.11328	-0.26863
C	2.16012	0.65891	0.42459
H	3.09726	0.09682	0.52819
H	2.75634	1.66588	-1.36678
H	1.53515	2.52771	-0.45178
C	2.45643	1.93919	-0.34962

C	3.53655	2.79476	0.30554
C	3.83471	4.07982	-0.46307
H	3.23435	3.04798	1.32941
H	4.45776	2.20661	0.39600
C	4.92480	4.93092	0.18447
H	4.13325	3.82814	-1.48802
H	2.91644	4.67417	-0.54915
C	5.21014	6.21295	-0.59392
H	4.62625	5.17934	1.20879
H	5.84161	4.33724	0.26688
H	4.31270	6.83340	-0.66379
H	5.99243	6.80779	-0.11824
H	5.53471	5.98478	-1.61256
H	1.84164	0.92435	1.44867
H	-0.93672	-1.68467	1.81467
H	-0.17050	0.81064	-0.21007
C	-8.53848	2.44058	0.28000
C	-7.30761	2.08456	-0.54979
C	-6.03434	2.02158	0.29082
C	-4.79470	1.66574	-0.52626
C	-3.52589	1.60087	0.32028
C	-2.29673	1.25051	-0.50731
N	-1.08442	1.15547	0.32020
H	-9.43903	2.48189	-0.33539
H	-8.41540	3.41469	0.76036
H	-8.70377	1.70082	1.06747
H	-7.17495	2.82151	-1.34918
H	-7.46109	1.11855	-1.04258
H	-6.16501	1.28406	1.09165
H	-5.87884	2.98831	0.78482
H	-4.66345	2.40412	-1.32623
H	-4.95160	0.69930	-1.01970
H	-3.64414	0.85595	1.11454
H	-3.36608	2.56665	0.81496
H	-2.14377	1.98738	-1.30073
H	-2.43640	0.27956	-0.98962
H	-1.05201	0.26484	1.03364
H	-0.89120	2.02395	0.81268

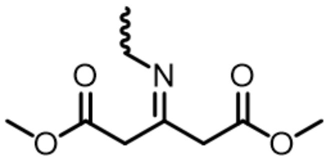

A2-1

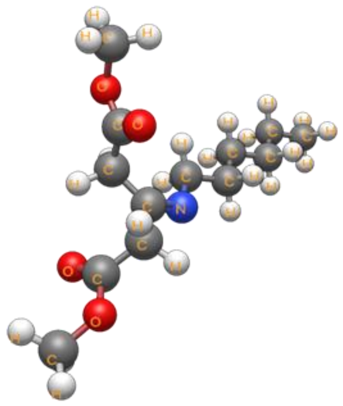

O	-4.40737	-2.06822	0.61694

C	-3.46272	-1.41413	-0.06999
C	-2.43157	-0.82180	0.86068
C	-1.23381	-0.26538	0.13423
C	-1.47116	0.95111	-0.74328
O	-3.46673	-1.32221	-1.26989
C	-1.42828	2.22181	0.07599
O	-0.95768	3.25303	-0.63665
O	-1.78975	2.31846	1.21862
C	-5.44118	-2.66266	-0.17136
C	-0.93169	4.50918	0.04755
N	-0.11085	-0.81200	0.33085
C	1.08354	-0.26348	-0.30279
H	-2.91519	-0.02429	1.43633
H	-2.46642	0.88661	-1.19089
H	-0.75419	1.01576	-1.55946
H	-5.01676	-3.38908	-0.86440
H	-5.97274	-1.89753	-0.73747
H	-6.10712	-3.14926	0.53581
H	-1.93878	4.79344	0.35273
H	-0.52928	5.22364	-0.66509
H	-0.29623	4.44396	0.93068
C	2.33430	-0.89597	0.29108
H	-2.10602	-1.58871	1.56336
H	7.47109	-0.91024	1.29531
C	3.61585	-0.36009	-0.33960
H	8.32466	-0.70244	-0.23702
C	4.87644	-0.99020	0.24756
H	3.59420	-0.53819	-1.42195
H	3.66244	0.72814	-0.20819
C	6.16331	-0.45515	-0.37646
H	4.89886	-0.81506	1.32995
H	4.83406	-2.07780	0.11355
C	7.41433	-1.09580	0.21955
H	6.13824	-0.62980	-1.45757
H	6.20413	0.63105	-0.24048
H	7.40497	-2.17866	0.07045
H	1.04900	-0.46485	-1.38174
H	1.13150	0.82758	-0.18127
H	2.34096	-0.71256	1.37042
H	2.27250	-1.98079	0.15986

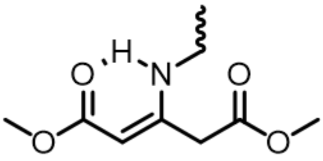

A2-2

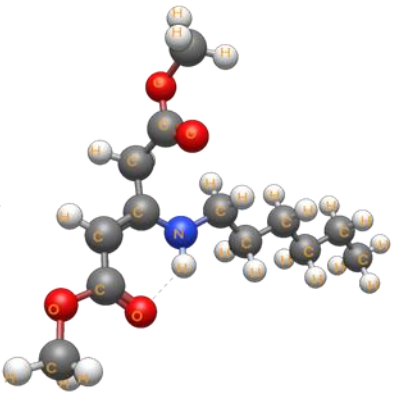

O	-4.33318	-2.22185	-0.03503
C	-3.04528	-1.81802	-0.02162
C	-2.89070	-0.46158	-0.49095
C	-1.67327	0.16074	-0.53541
C	-1.60991	1.58265	-1.02744
O	-2.15090	-2.55891	0.35593
C	-1.25624	2.57769	0.05809
O	-1.13053	3.80799	-0.46019
O	-1.11347	2.33052	1.22268
C	-4.56059	-3.55086	0.42328
C	-0.83002	4.84139	0.48197
N	-0.51300	-0.44111	-0.20478
C	0.77354	0.20380	0.00073
H	1.85531	-1.22443	-1.18480
H	-2.57912	1.87415	-1.43423
H	-0.88702	1.69136	-1.84037
H	-4.23129	-3.66167	1.45709
H	-4.02175	-4.26826	-0.19683
H	-5.63332	-3.70985	0.34594
H	-1.61760	4.90793	1.23254
H	-0.77103	5.75912	-0.09643
H	0.11893	4.63593	0.97749
C	1.90327	-0.80329	-0.17543
H	-3.77767	0.06792	-0.80237
H	6.81294	-2.39346	0.69097
C	3.27378	-0.17782	0.06907
H	7.90086	-1.10634	0.16300
C	4.41790	-1.17553	-0.09342
H	3.42283	0.65859	-0.62472
H	3.30551	0.24817	1.07878
C	5.79227	-0.55593	0.14943
H	4.27045	-2.01210	0.60004
H	4.38681	-1.60354	-1.10259
C	6.92610	-1.56513	-0.01315
H	5.93822	0.27877	-0.54476
H	5.82033	-0.12787	1.15726
H	6.93050	-1.98591	-1.02199
H	0.89858	1.01419	-0.72334
H	0.81988	0.65225	0.99977
H	1.75217	-1.63539	0.52188
H	-0.62389	-1.38244	0.16322

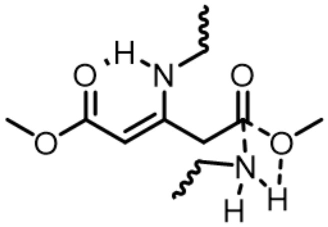

TSA3

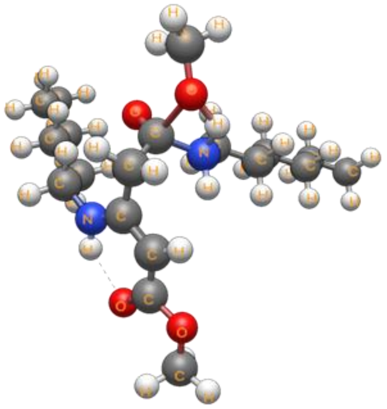

O	0.55873	-4.74775	-1.34150
C	-0.38207	-3.78629	-1.21620
C	-0.45836	-3.26399	0.12539
C	-1.34884	-2.27510	0.48666
C	-1.28137	-1.76155	1.90744
O	-1.06046	-3.43410	-2.16956
C	-0.57922	-0.40575	1.95730
O	0.14683	-0.54205	3.67629
O	-1.12397	0.66311	1.77706
C	0.68043	-5.31710	-2.64167
C	-0.10952	0.57053	4.48729
N	-2.22117	-1.72667	-0.37196
C	-3.12314	-0.60695	-0.14289
H	-2.75826	0.40057	-2.01546
H	-0.72720	-2.47286	2.51569
H	-2.26747	-1.62926	2.35171
H	0.95270	-4.55394	-3.37176
H	-0.25935	-5.77772	-2.94816
H	1.46414	-6.06650	-2.56302
H	-0.77971	1.26801	3.96326
H	0.81254	1.10919	4.73574
H	-0.58730	0.25944	5.42143
C	-2.71979	0.62879	-0.94395
H	0.16643	-3.72120	0.87861
H	-2.71232	5.80600	-1.67775
C	-3.61738	1.82025	-0.62516
H	-4.38810	6.36315	-1.65336
C	-3.24482	3.07005	-1.41843
H	-4.66421	1.56119	-0.82759
H	-3.54502	2.03577	0.44705
C	-4.13276	4.27045	-1.09861
H	-2.19936	3.32844	-1.21122
H	-3.30321	2.85278	-2.49208
C	-3.74383	5.51491	-1.89283
H	-5.17659	4.01117	-1.30640
H	-4.07486	4.48325	-0.02589
H	-3.81876	5.32960	-2.96761
H	-4.13567	-0.91847	-0.42027
H	-3.12875	-0.35329	0.91506
H	-1.68480	0.87725	-0.69389
H	-2.17589	-2.10757	-1.31407

C	7.09646	2.54851	-1.46307
C	5.60437	2.71209	-1.18533
C	5.00986	1.50806	-0.45817
C	3.51809	1.66004	-0.17189
C	2.92983	0.45267	0.55509
C	1.44462	0.62934	0.83237
N	0.89898	-0.54060	1.54072
H	7.50546	3.41709	-1.98238
H	7.28248	1.66826	-2.08382
H	7.65385	2.42152	-0.53143
H	5.06737	2.86397	-2.12773
H	5.43663	3.61299	-0.58548
H	5.54712	1.35378	0.48532
H	5.17503	0.60515	-1.05828
H	2.98102	1.81438	-1.11500
H	3.35353	2.56148	0.42978
H	3.45918	0.29626	1.50133
H	3.08711	-0.44855	-0.04937
H	0.88255	0.77347	-0.09453
H	1.25852	1.50102	1.46147
H	1.06977	-0.57356	2.70207
H	1.06222	-1.40880	1.02840

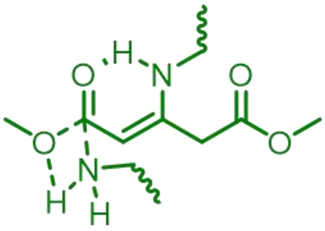

TSA3i

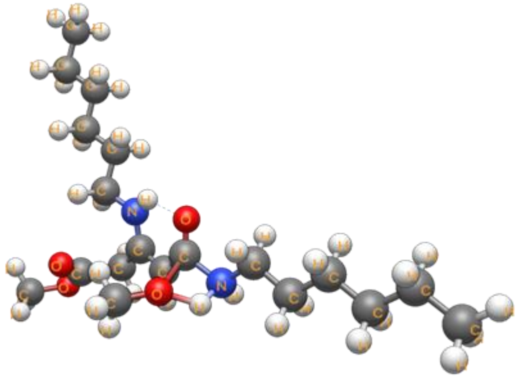

O	1.80141	-1.50542	1.51140
C	1.19390	-0.40415	-0.02717
C	0.29756	-1.36894	-0.65695
C	-1.04438	-1.15896	-0.74805
C	-1.90748	-2.24733	-1.32989
O	0.91032	0.72748	0.31690
C	-2.77570	-2.93566	-0.29694
O	-3.56197	-3.85157	-0.88407
O	-2.77596	-2.72152	0.88247
C	0.85952	-2.29552	2.15497
C	-4.40831	-4.58802	0.00265
N	-1.65191	-0.00003	-0.38849
C	-3.07484	0.17951	-0.15143
H	-3.16487	2.04713	-1.20825
H	-1.27862	-3.01264	-1.78718

H	-2.55679	-1.86527	-2.12223
H	-0.13451	-1.82211	2.12463
H	1.12377	-2.44136	3.21075
H	0.74959	-3.29252	1.69817
H	-3.80859	-5.12605	0.73683
H	-4.96126	-5.28121	-0.62521
H	-5.08789	-3.91319	0.52338
C	-3.43740	1.65769	-0.22202
H	0.69625	-2.33355	-0.94131
H	-6.57707	5.67936	0.94506
C	-4.91946	1.90128	0.04883
H	-8.20542	5.27310	0.39500
C	-5.29755	3.37933	-0.00778
H	-5.52045	1.34679	-0.68238
H	-5.18154	1.49814	1.03409
C	-6.77999	3.62994	0.25939
H	-4.69903	3.93464	0.72428
H	-5.03346	3.78491	-0.99192
C	-7.14312	5.11164	0.20206
H	-7.37622	3.07490	-0.47304
H	-7.04158	3.22329	1.24220
H	-6.91303	5.53065	-0.78101
H	-3.64537	-0.36493	-0.90993
H	-3.36035	-0.23145	0.82430
H	-2.83697	2.20769	0.51181
H	-1.02974	0.65921	0.07155
C	9.85905	1.64273	-0.20868
C	8.40050	2.00350	0.06120
C	7.44320	0.86395	-0.28057
C	5.98057	1.21291	-0.01748
C	5.02871	0.06774	-0.35658
C	3.57960	0.44223	-0.08678
N	2.67692	-0.68221	-0.39439
H	10.52931	2.46719	0.04140
H	10.01015	1.39544	-1.26270
H	10.16057	0.77421	0.38253
H	8.12559	2.89058	-0.51947
H	8.27544	2.27350	1.11515
H	7.71515	-0.02417	0.30238
H	7.56847	0.59072	-1.33550
H	5.70747	2.09948	-0.60165
H	5.85509	1.48534	1.03651
H	5.29000	-0.81539	0.23651
H	5.15369	-0.20699	-1.41149
H	3.26719	1.30963	-0.67191
H	3.41490	0.67644	0.96517
H	2.71336	-1.46363	0.39203
H	2.76497	-1.00715	-1.35452

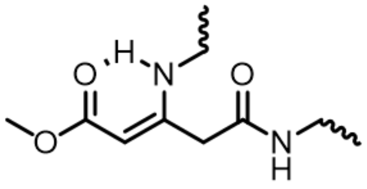

A3-1

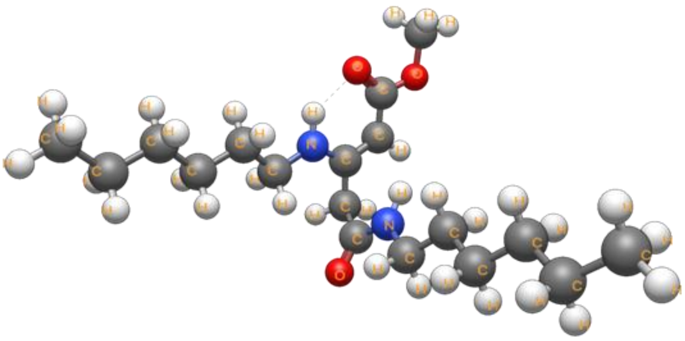

O	-0.49483	4.71157	-1.16744
C	0.28221	3.64901	-0.87103
C	0.10954	3.20242	0.49085
C	0.81127	2.14046	1.00463
C	0.54823	1.69411	2.41850
O	1.02998	3.15548	-1.70134
C	-0.29480	0.41649	2.51195
H	-1.34812	0.99142	0.85672
O	-0.11519	-0.39801	3.39691
C	-0.36989	5.20827	-2.49691
H	-2.67912	-0.90881	2.52506
N	1.69419	1.41385	0.29623
C	2.46619	0.28883	0.80479
H	3.78923	0.27466	-0.88861
H	0.01401	2.48093	2.95334
H	1.47424	1.49135	2.95695
H	-0.64094	4.43921	-3.22117
H	0.65383	5.53003	-2.69123
H	-1.05326	6.05123	-2.56181
H	-1.55945	-1.78179	1.49097
H	-2.58719	-0.70397	-0.53157
H	-3.71513	0.16187	0.50840
C	3.15196	-0.43357	-0.34669
H	-0.57187	3.75035	1.12410
H	5.47440	-4.65121	-2.39527
C	3.99142	-1.61370	0.13474
H	6.80850	-5.10462	-1.33058
C	4.68259	-2.35219	-1.00876
H	4.74708	-1.25852	0.84519
H	3.35324	-2.31391	0.68622
C	5.52465	-3.53442	-0.53434
H	3.92800	-2.70782	-1.72064
H	5.32065	-1.65178	-1.56105
C	6.21090	-4.26343	-1.68672
H	6.27689	-3.17739	0.17721
H	4.88567	-4.23266	0.01677
H	6.87412	-3.58849	-2.23392
H	3.22133	0.63439	1.52199
H	1.81089	-0.40944	1.33495
H	2.39050	-0.78154	-1.05291
H	1.85400	1.72674	-0.65696
C	-7.04875	-2.95305	-1.98524
C	-6.05224	-3.05617	-0.83342

C	-5.09532	-1.86733	-0.77970
C	-4.09388	-1.95575	0.36910
C	-3.13885	-0.76582	0.41377
C	-2.15185	-0.86449	1.56716
N	-1.25517	0.28132	1.57063
H	-7.72492	-3.80967	-2.00927
H	-6.52946	-2.90842	-2.94606
H	-7.65605	-2.04877	-1.89416
H	-5.47227	-3.98047	-0.92802
H	-6.59198	-3.12646	0.11704
H	-5.67477	-0.94090	-0.68616
H	-4.55292	-1.79635	-1.73027
H	-3.51588	-2.88259	0.27647
H	-4.63639	-2.02278	1.31926

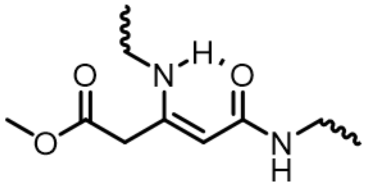

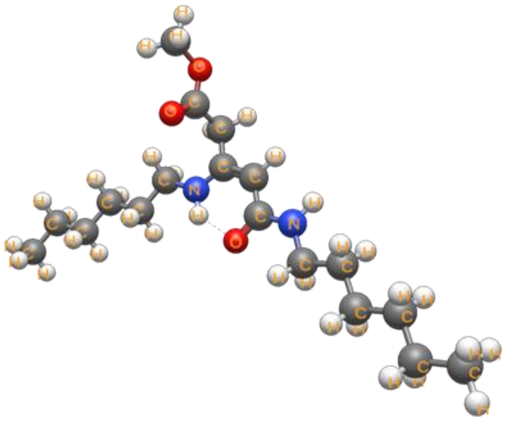

A3-2

O	-2.79565	4.39426	0.01860
C	-2.33954	3.15303	0.24828
C	-1.73025	2.57723	-1.01425
C	-0.92805	1.32485	-0.76917
C	0.43550	1.41205	-0.74224
O	-2.42856	2.60262	1.30962
C	1.28706	0.25518	-0.50079
H	2.97482	1.41182	-0.68974
O	0.85836	-0.88985	-0.32190
C	-3.41134	5.03596	1.13813
H	3.54532	-1.27503	-1.14587
N	-1.60962	0.17715	-0.59276
C	-3.05337	0.02562	-0.60742
H	-3.02933	-1.57578	0.81651
H	5.99751	-1.82679	-0.77953
H	0.89308	2.38049	-0.89358
H	-2.70080	5.12366	1.96003
H	-4.27581	4.46306	1.47422
H	-3.71472	6.01769	0.78510
H	3.28722	-1.16890	0.58807
H	5.01091	0.63796	0.74354
H	5.26307	0.55528	-0.99887
C	-3.42743	-1.38948	-0.18641
H	-2.55076	2.39420	-1.71493

H	-6.76395	-5.41710	-0.04167
C	-4.93656	-1.61453	-0.19495
H	-8.28970	-4.83335	0.62999
C	-5.32539	-3.03056	0.22284
H	-5.41594	-0.89429	0.47858
H	-5.33140	-1.41297	-1.19808
C	-6.83453	-3.26338	0.21989
H	-4.84642	-3.75194	-0.45007
H	-4.92939	-3.23474	1.22475
C	-7.20831	-4.68379	0.63628
H	-7.31059	-2.54344	0.89435
H	-7.22854	-3.05620	-0.78102
H	-6.84509	-4.90148	1.64398
H	-3.51484	0.73853	0.08303
H	-3.45314	0.21739	-1.61226
H	-2.94310	-2.10466	-0.86069
H	-1.02395	-0.63594	-0.41434
C	9.89477	-1.16208	0.62771
C	8.48111	-1.71229	0.45608
C	7.44406	-0.60990	0.25336
C	6.02528	-1.14584	0.07912
C	4.99219	-0.03960	-0.11765
C	3.58395	-0.59014	-0.29075
N	2.63203	0.48977	-0.47399
H	10.62313	-1.96245	0.77129
H	9.95090	-0.49795	1.49415
H	10.19740	-0.58678	-0.25117
H	8.20662	-2.30636	1.33432
H	8.45169	-2.39524	-0.39972
H	7.71810	-0.01335	-0.62540
H	7.47158	0.07402	1.11037
H	5.75274	-1.74341	0.95671
H	-1.10154	3.34534	-1.46363

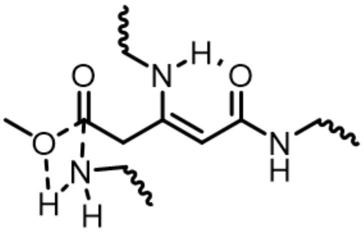

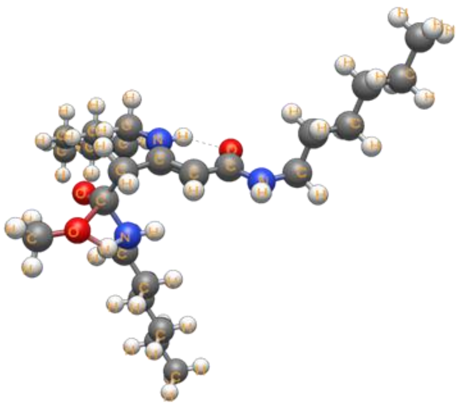

TSA4

O	2.58909	0.19498	3.76084
C	1.94133	-0.57768	2.16892
C	0.57303	-1.02143	2.68285
C	-0.46148	-0.93538	1.58224
C	-1.36424	0.10257	1.63341
O	2.76186	-1.30616	1.65405

C	-2.37027	0.32946	0.60605
H	-3.22272	1.81165	1.72828
O	-2.47072	-0.35373	-0.41904
C	3.87499	-0.23377	4.10819
H	-3.99696	1.48643	-1.09069
N	-0.42070	-1.82005	0.57203
C	0.49725	-2.93356	0.38958
H	1.93694	-1.80246	-0.71609
H	-6.32950	0.79710	-1.79740
H	-1.36123	0.73813	2.50965
H	4.20445	-1.02570	3.41871
H	3.88375	-0.63252	5.12772
H	4.60337	0.58462	4.05461
H	-4.65766	2.66148	0.04925
H	-5.86941	0.80958	1.22925
H	-5.17111	-0.34928	0.09772
C	1.39691	-2.74638	-0.82968
H	0.68942	-2.03274	3.07190
H	4.64455	-4.66306	-4.46094
C	2.39793	-3.88893	-0.96840
H	5.94195	-5.52268	-3.62560
C	3.30980	-3.73415	-2.18282
H	3.00892	-3.93233	-0.05933
H	1.86283	-4.84441	-1.03679
C	4.31787	-4.87224	-2.32478
H	2.70008	-3.67533	-3.09283
H	3.84812	-2.78153	-2.10891
C	5.22895	-4.70055	-3.53777
H	4.92249	-4.93119	-1.41354
H	3.77857	-5.82279	-2.39974
H	5.79753	-3.76953	-3.46747
H	1.12299	-3.04230	1.27324
H	-0.08956	-3.85230	0.28033
H	0.78017	-2.67022	-1.73263
H	-1.12482	-1.65444	-0.14560
C	-10.21264	-0.68653	-1.22728
C	-9.03158	0.24554	-1.48625
C	-7.87262	0.00478	-0.52150
C	-6.68295	0.92936	-0.76792
C	-5.52634	0.68090	0.19604
C	-4.34911	1.61894	-0.06580
N	-3.21810	1.38539	0.81556
H	-11.03167	-0.50235	-1.92525
H	-10.59897	-0.55203	-0.21353
H	-9.91204	-1.73235	-1.33105
H	-9.35845	1.28780	-1.40306
H	-8.67620	0.11477	-2.51401
H	-7.54291	-1.03771	-0.60425
H	-8.22671	0.13449	0.50852
H	-7.01339	1.97255	-0.68573
H	0.27496	-0.37354	3.50379
C	4.56113	6.40550	-2.70372
C	4.61284	4.91922	-2.35936
C	3.66414	4.54960	-1.22131
C	3.70314	3.06469	-0.86923

C	2.75681	2.70366	0.27371
C	2.80369	1.21830	0.59849
N	1.89955	0.89603	1.71514
H	5.24524	6.65278	-3.51751
H	4.83461	7.01509	-1.83861
H	3.55406	6.69865	-3.01136
H	5.63450	4.63969	-2.08074
H	4.36071	4.32571	-3.24462
H	2.64078	4.83066	-1.49775
H	3.91711	5.14085	-0.33295
H	4.72649	2.78244	-0.59585
H	3.44491	2.47433	-1.75603
H	1.73315	2.98793	0.00217
H	3.02012	3.27947	1.16775
H	3.80605	0.90766	0.89737
H	2.52221	0.61410	-0.26797
H	0.94052	1.19927	1.53549
H	2.32327	1.09366	2.78424

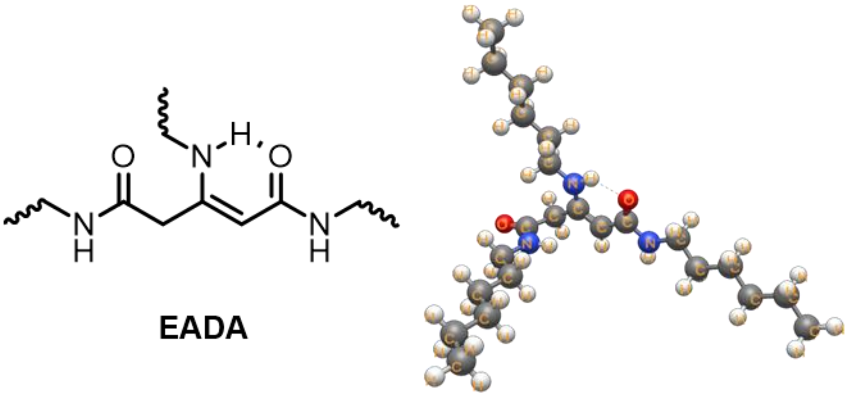

N	-3.00609	1.10239	1.01307
C	-1.67854	1.38999	0.86120
C	-0.80277	0.97383	1.95021
C	0.54615	1.21214	1.94380
C	1.39746	0.71348	3.08203
O	-1.27803	1.95714	-0.16037
C	2.24138	-0.51823	2.73586
H	0.82570	-1.11655	1.38717
O	3.30697	-0.72875	3.28338
C	-3.99393	1.55337	0.04924
H	2.58506	-3.18263	2.31125
N	1.19218	1.82038	0.92889
C	2.61454	2.11975	0.89153
H	2.40640	3.48467	-0.75399
H	0.75939	0.44326	3.92501
H	2.09675	1.47590	3.42729
H	2.46416	-5.26124	0.83125
H	-3.32696	0.77033	1.90892
C	-5.31576	0.82548	0.24960
H	3.37577	-2.31523	1.00498
H	1.37855	-2.71178	-0.46686

H	0.59498	-3.59179	0.84237
C	3.00572	2.60348	-0.49824
H	-1.23072	0.47739	2.81118
H	6.56130	3.49268	-4.23102
C	4.48966	2.94657	-0.59298
H	7.83551	4.49999	-3.53750
C	4.89618	3.43066	-1.98264
H	4.73383	3.71963	0.14513
H	5.08551	2.06568	-0.32677
C	6.38015	3.77532	-2.08630
H	4.65136	2.65893	-2.72236
H	4.30033	4.31193	-2.24917
C	6.77244	4.25860	-3.48024
H	6.62294	4.54548	-1.34631
H	6.97338	2.89394	-1.82007
H	6.20962	5.15486	-3.75377
H	2.86858	2.89027	1.63089
H	3.19723	1.22665	1.14201
H	2.75543	1.82649	-1.22853
H	0.59384	2.13511	0.16833
C	1.29799	-7.49074	-2.52633
C	2.13062	-6.70837	-1.51388
C	1.44223	-5.42372	-1.05818
C	2.26294	-4.63181	-0.04340
C	1.57017	-3.34780	0.40526
C	2.39683	-2.57424	1.42142
N	1.70875	-1.35572	1.81794
H	1.80408	-8.40505	-2.84181
H	1.10458	-6.88935	-3.41839
H	0.33150	-7.77223	-2.10025
H	3.10411	-6.45937	-1.94970
H	2.33536	-7.33600	-0.63992
H	0.46662	-5.67135	-0.62259
H	1.23784	-4.79330	-1.93201
H	3.23852	-4.38563	-0.47852
H	-4.14189	2.63759	0.12900
H	-3.59618	1.36264	-0.94966
C	-6.38214	1.28315	-0.74210
H	-5.15076	-0.25244	0.14562
H	-5.67780	0.99530	1.27159
C	-7.71309	0.55769	-0.56118
H	-6.53993	2.36273	-0.63524
H	-6.01802	1.12375	-1.76373
C	-8.78217	1.00997	-1.55336
H	-7.55559	-0.52251	-0.66728
H	-8.07831	0.71650	0.46085
C	-10.10686	0.27578	-1.36200
H	-8.93858	2.08873	-1.44622
H	-8.41563	0.85128	-2.57320
H	-10.50333	0.44411	-0.35731
H	-10.85892	0.60997	-2.07920
H	-9.97623	-0.80186	-1.49069

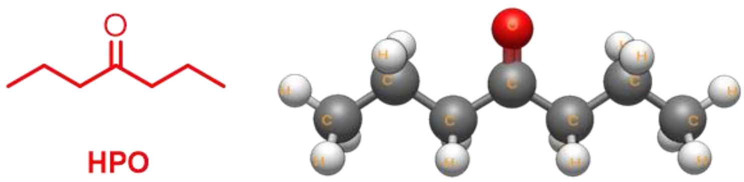

HPO

C	-3.81664	-0.50956	-0.00001
C	-2.54620	0.33633	-0.00000
C	-1.28996	-0.52391	0.00001
C	-0.00000	0.27661	0.00001
O	-0.00000	1.48303	-0.00000
C	1.28996	-0.52391	0.00001
C	2.54620	0.33633	-0.00000
C	3.81664	-0.50956	-0.00001
H	-4.70969	0.11778	-0.00001
H	-3.86083	-1.15327	0.88261
H	-3.86082	-1.15327	-0.88262
H	-2.52685	0.99427	0.87217
H	-2.52684	0.99427	-0.87217
H	-1.27224	-1.18944	-0.87228
H	-1.27225	-1.18944	0.87230
H	1.27224	-1.18945	-0.87228
H	1.27225	-1.18944	0.87230
H	2.52685	0.99427	0.87217
H	2.52684	0.99427	-0.87218
H	3.86083	-1.15327	0.88261
H	4.70969	0.11778	-0.00001
H	3.86082	-1.15327	-0.88262

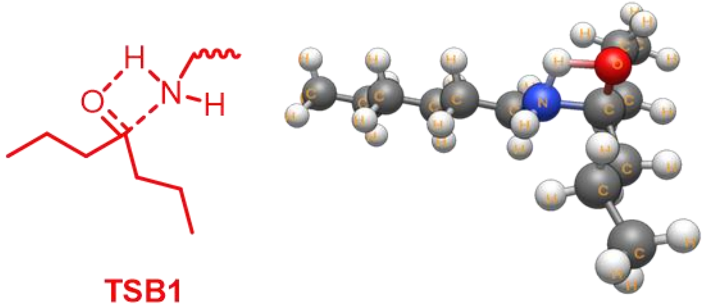

TSB1

C	1.76609	3.94489	-0.50048
C	1.87292	2.66342	0.32212
C	2.27818	1.46776	-0.53669
C	2.30523	0.13600	0.23041
O	2.67665	0.23470	1.52486
C	3.02023	-0.94922	-0.58447
C	3.02429	-2.33394	0.05886
C	4.05415	-3.26099	-0.58390
N	0.81374	-0.29153	0.55159
C	-0.37219	0.12335	-0.21260
C	-1.65064	-0.42131	0.41134
C	-2.89715	0.01703	-0.35400
C	-4.18688	-0.52844	0.25424

C	-5.43809	-0.08686	-0.50165
C	-6.71944	-0.64163	0.11530
H	1.51266	4.80285	0.12540
H	0.99856	3.85638	-1.27526
H	2.71330	4.16474	-1.00037
H	0.92135	2.47075	0.83011
H	2.60952	2.77047	1.11964
H	3.29637	1.63490	-0.90470
H	1.64362	1.38734	-1.42911
H	4.05567	-0.60492	-0.67699
H	2.60971	-0.99343	-1.60182
H	2.03665	-2.80482	-0.03033
H	3.23449	-2.21114	1.12466
H	3.87170	-3.36968	-1.65657
H	4.03112	-4.25644	-0.13630
H	5.06207	-2.85896	-0.45690
H	-0.26763	-0.23296	-1.24397
H	-0.40042	1.21304	-0.24211
H	-1.71279	-0.08042	1.45018
H	-1.60599	-1.51670	0.43986
H	-2.82049	-0.31241	-1.39701
H	-2.94211	1.11214	-0.37726
H	-4.26154	-0.20367	1.29893
H	-4.14438	-1.62412	0.27454
H	-5.36048	-0.40965	-1.54545
H	-5.47991	1.00744	-0.51898
H	-6.70877	-1.73462	0.11742
H	-7.60359	-0.31636	-0.43591
H	-6.82836	-0.30880	1.15069
H	0.81947	-1.30548	0.65930
H	1.27063	0.10716	1.56743

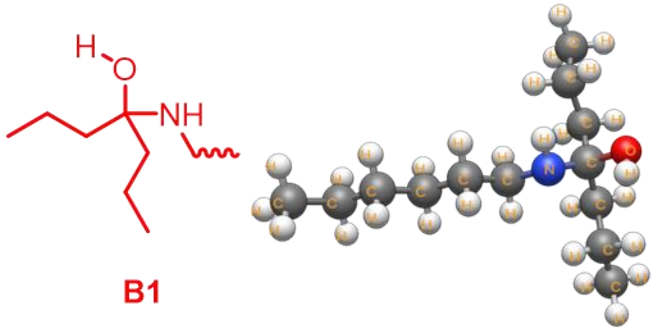

B1

C	2.51904	3.82044	-0.70263
C	2.05239	2.59595	0.08160
C	2.49870	1.29379	-0.57739
C	2.23604	0.03524	0.26405
O	3.11630	0.13340	1.37319
C	2.55020	-1.21777	-0.56462
C	2.36275	-2.53762	0.17861
C	2.68971	-3.73737	-0.70808
N	0.88792	-0.05375	0.82643
C	-0.21135	-0.14848	-0.12806

C	-1.54841	-0.00346	0.58666
C	-2.73913	-0.13897	-0.35854
C	-4.08262	0.01552	0.34998
C	-5.27716	-0.12272	-0.59126
C	-6.61317	0.03548	0.13028
H	2.17650	4.74754	-0.23901
H	2.13811	3.79670	-1.72731
H	3.61001	3.85384	-0.75461
H	0.95889	2.61538	0.15842
H	2.44806	2.63176	1.10086
H	3.57621	1.33046	-0.76806
H	2.01020	1.18488	-1.55177
H	2.82559	-0.52492	2.01348
H	3.58996	-1.12592	-0.89526
H	1.93312	-1.21557	-1.47013
H	1.33538	-2.60830	0.54668
H	3.01209	-2.55480	1.06025
H	2.03765	-3.76180	-1.58545
H	2.56258	-4.67901	-0.17058
H	3.72215	-3.69058	-1.06411
H	0.75034	0.76141	1.41860
H	-0.16968	-1.12889	-0.61440
H	-0.14533	0.60911	-0.92649
H	-1.58719	0.97571	1.08102
H	-1.60810	-0.75802	1.37771
H	-2.70140	-1.11756	-0.85195
H	-2.66213	0.61143	-1.15446
H	-4.12260	0.99429	0.84337
H	-4.16125	-0.73373	1.14695
H	-5.23630	-1.10091	-1.08245
H	-5.19612	0.62559	-1.38706
H	-6.72443	-0.72010	0.91232
H	-7.45573	-0.06636	-0.55636
H	-6.68443	1.01726	0.60561

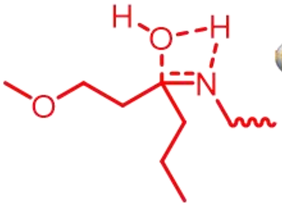

TSB2

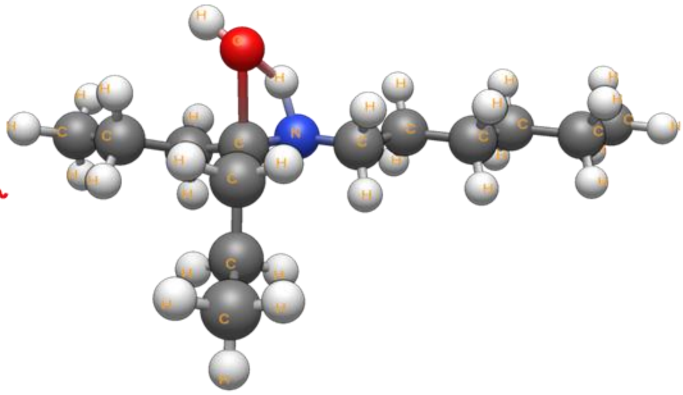

C	2.44454	3.52680	0.05746
C	2.19070	2.13022	-0.50342
C	2.03070	1.09608	0.62304
C	1.73852	-0.26469	0.05618
O	1.62397	-1.43002	1.65036

C	2.86322	-0.96387	-0.66464
C	4.25154	-0.83884	-0.04618
C	5.26045	-1.74906	-0.74279
N	0.51750	-0.69475	-0.24117
C	-0.63366	0.09598	0.20204
C	-1.92080	-0.57426	-0.25719
C	-3.16680	0.20188	0.15887
C	-4.46338	-0.46878	-0.28831
C	-5.71416	0.30528	0.12164
C	-7.00190	-0.37933	-0.32985
H	2.57085	4.25833	-0.74254
H	3.34761	3.53957	0.67278
H	1.60997	3.85182	0.68330
H	3.02158	1.83479	-1.15182
H	1.29475	2.13509	-1.13156
H	1.22935	1.39384	1.29978
H	2.94916	1.04820	1.20953
H	2.26173	-2.13585	1.80663
H	2.86880	-0.53845	-1.67815
H	2.57394	-2.01095	-0.78907
H	4.19457	-1.08080	1.01956
H	4.59584	0.19820	-0.11048
H	4.96752	-2.79786	-0.65092
H	6.25765	-1.63979	-0.31323
H	5.32669	-1.51617	-1.80874
H	0.76915	-1.49849	0.64537
H	-0.58526	1.10894	-0.21484
H	-0.64831	0.19470	1.29696
H	-1.95759	-1.58698	0.15904
H	-1.89074	-0.68551	-1.34581
H	-3.12305	1.21532	-0.25915
H	-3.17866	0.31883	1.24939
H	-4.51074	-1.48112	0.13083
H	-4.45285	-0.58827	-1.37852
H	-5.66593	1.31545	-0.29941
H	-5.72178	0.42524	1.21043
H	-7.02389	-0.48696	-1.41750
H	-7.88582	0.18761	-0.03111
H	-7.08245	-1.37967	0.10344

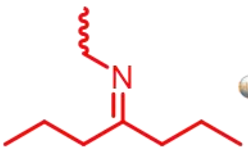

B2

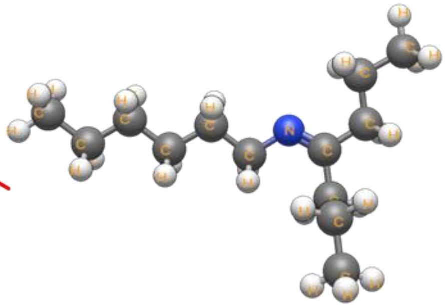

C	1.50624	-0.54415	-0.08089
C	0.27870	0.23551	-0.53288
N	-0.92755	-0.55923	-0.33119

C	-2.06703	-0.00714	-0.40665
C	-3.30993	-0.84567	-0.21092
C	-3.06127	-2.28072	0.23134
C	-4.36556	-3.05537	0.40280
C	-2.31868	1.46721	-0.65576
C	-2.39129	2.25576	0.66133
C	-2.64206	3.74229	0.42409
C	2.80360	0.23135	-0.28828
C	4.04072	-0.54320	0.15945
C	5.34186	0.22957	-0.04482
C	6.56929	-0.55828	0.40623
H	-2.69871	4.29182	1.36555
H	-3.58103	3.89754	-0.11336
H	-1.83935	4.18117	-0.17398
H	1.53935	-1.49099	-0.62904
H	1.38801	-0.80202	0.97689
H	0.37728	0.47859	-1.59974
H	0.23661	1.19103	0.00659
H	-3.86909	-0.83465	-1.15665
H	-3.95986	-0.33299	0.51131
H	-2.49572	-2.27177	1.16660
H	-2.41677	-2.77152	-0.50093
H	-5.00823	-2.58576	1.15273
H	-4.17837	-4.08259	0.72139
H	-4.92611	-3.09457	-0.53533
H	-3.26805	1.58269	-1.18874
H	-1.53766	1.88981	-1.29329
H	-1.46017	2.11749	1.21877
H	-3.18774	1.83949	1.28562
H	2.75562	1.18075	0.25966
H	2.90728	0.49407	-1.34852
H	4.09290	-1.49131	-0.38927
H	3.93926	-0.80745	1.21904
H	5.28793	1.17576	0.50459
H	5.44079	0.49317	-1.10347
H	6.50130	-0.80916	1.46803
H	7.49010	0.00821	0.25395
H	6.65568	-1.49521	-0.15023

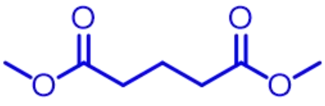

DG

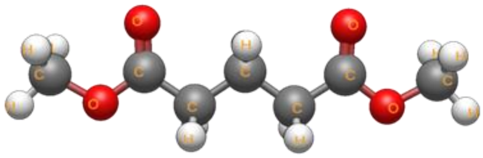

O	-3.60867	-0.69039	0.00056
C	-2.52064	0.09791	-0.00027
C	-1.25467	-0.72249	-0.00031
C	-0.00001	0.13965	0.00011
C	1.25466	-0.72251	-0.00055
O	-2.57801	1.29696	-0.00096
C	2.52063	0.09793	0.00006
O	3.60868	-0.69039	-0.00008

O	2.57801	1.29695	0.00058
C	-4.86180	-0.00420	0.00057
C	4.86180	-0.00420	0.00021
H	-1.29034	-1.38199	0.87283
H	-1.29013	-1.38134	-0.87397
H	0.00010	0.79789	0.87103
H	-0.00008	0.79901	-0.86995
H	1.29025	-1.38236	0.87232
H	1.29021	-1.38093	-0.87452
H	-5.62339	-0.77925	0.00210
H	-4.95103	0.62149	-0.88776
H	-4.94977	0.62380	0.88739
H	5.62341	-0.77923	-0.00109
H	4.95080	0.62150	0.88856
H	4.95001	0.62379	-0.88658

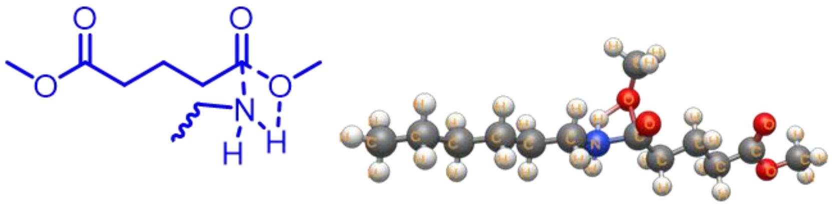

TSC1

N	-0.98373	-0.27796	-0.38191
C	0.35016	0.07936	0.35877
C	1.50380	-0.75505	-0.14875
C	2.83131	-0.09741	0.20592
C	4.01020	-0.93215	-0.27291
O	0.25106	0.52670	1.47676
C	5.34054	-0.30121	0.05356
O	6.35898	-1.06806	-0.37643
O	5.50309	0.74463	0.61969
C	-2.19649	-0.10408	0.43391
C	7.66460	-0.55203	-0.11550
C	-3.45278	-0.36883	-0.38233
C	-4.72047	-0.17073	0.44611
C	-5.99534	-0.43013	-0.35254
C	-7.26659	-0.23206	0.47000
C	-8.53353	-0.49311	-0.34051
O	0.40627	1.42544	-0.94911
C	0.47829	2.71961	-0.42209
H	1.39106	3.22297	-0.75968
H	1.41736	-0.88300	-1.23118
H	1.42397	-1.74205	0.32616
H	2.88787	0.05233	1.28563
H	2.86176	0.89304	-0.25333
H	3.97916	-1.08523	-1.35661
H	4.00411	-1.93243	0.17241
H	-0.71025	0.62624	-1.03845
H	-2.17640	0.91984	0.81000
H	-2.13652	-0.76321	1.30354
H	7.79484	0.41462	-0.60275

H	8.35869	-1.28279	-0.52203
H	7.81764	-0.43136	0.95726
H	-3.43193	-1.39364	-0.77326
H	-3.46982	0.30000	-1.24977
H	-4.73942	0.85213	0.83936
H	-4.69479	-0.83642	1.31667
H	-5.97603	-1.45321	-0.74745
H	-6.02047	0.23586	-1.22342
H	-7.28387	0.78957	0.86419
H	-7.23989	-0.89785	1.33924
H	-8.59231	0.18152	-1.19849
H	-9.43200	-0.34786	0.26197
H	-8.54750	-1.51737	-0.72199
H	-0.38075	3.32786	-0.73025
H	0.48981	2.67868	0.67749
H	-0.94555	-1.18530	-0.84098

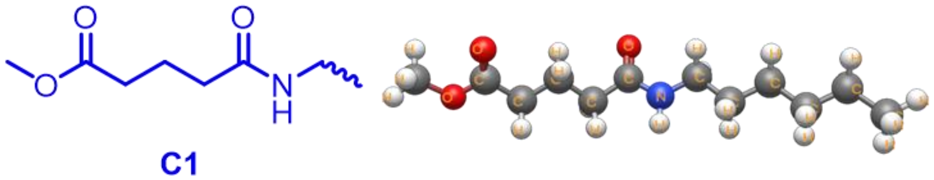

C1

N	0.83958	-0.31374	0.52337
C	-0.33872	-0.92262	0.22957
C	-1.56690	-0.38069	0.94294
C	-2.74633	-0.23626	-0.01626
C	-4.02149	0.14247	0.72366
O	-0.40403	-1.85516	-0.54815
C	-5.18492	0.37712	-0.20749
O	-6.33041	0.53650	0.47728
O	-5.12402	0.43195	-1.40527
C	2.09123	-0.77240	-0.05932
C	-7.48994	0.78481	-0.31919
C	3.23498	0.15406	0.32554
C	4.56784	-0.30146	-0.26293
C	5.72679	0.61971	0.10958
C	7.06278	0.16743	-0.47569
C	8.21250	1.09747	-0.09681
H	0.85657	0.44662	1.18382
H	-1.34677	0.57272	1.43328
H	-1.81486	-1.10074	1.73068
H	-2.88416	-1.17741	-0.55119
H	-2.52336	0.51916	-0.77362
H	-3.88893	1.06142	1.30577
H	-4.31395	-0.62874	1.44213
H	1.97496	-0.81301	-1.14593
H	2.29559	-1.79515	0.27444
H	-7.36464	1.70136	-0.89629
H	-8.31481	0.88241	0.38146
H	-7.66324	-0.04499	-1.00468
H	3.31765	0.19793	1.41845
H	3.01041	1.16976	-0.02021

H	4.48262	-0.35481	-1.35437
H	4.78772	-1.31886	0.08060
H	5.81115	0.67531	1.20176
H	5.50759	1.63766	-0.23485
H	6.97661	0.11141	-1.56606
H	7.28045	-0.84901	-0.13067
H	8.02602	2.11283	-0.45617
H	9.15905	0.75998	-0.52306
H	8.33197	1.14626	0.98866

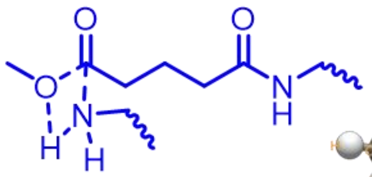

TSC2

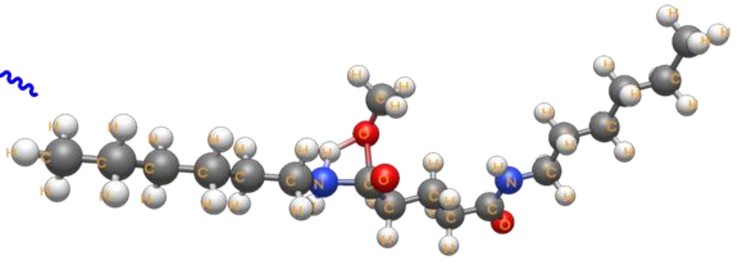

N	3.48463	1.47998	-0.08492
C	2.97817	2.73748	-0.02952
C	1.46262	2.87947	-0.02443
C	0.63506	1.60258	-0.12829
C	-0.86021	1.89479	-0.11448
O	3.69917	3.71751	0.01906
C	-1.64265	0.60860	-0.24618
N	-3.11610	0.77958	0.24021
O	-1.42868	-0.25711	-1.06176
C	4.92111	1.25273	-0.09502
C	-4.08723	-0.09680	-0.43693
C	5.22729	-0.23741	-0.10893
C	6.72766	-0.51818	-0.12079
C	7.05114	-2.01005	-0.13257
C	8.55071	-2.29833	-0.14429
C	8.85903	-3.79344	-0.15539
C	-5.47647	0.05357	0.16447
C	-6.48996	-0.86699	-0.51241
C	-7.89239	-0.73624	0.07631
C	-8.90993	-1.65606	-0.59486
C	-10.30721	-1.51677	0.00380
O	-1.41314	-0.01030	1.52614
C	-0.91920	-1.31516	1.60492
H	-10.67503	-0.49274	-0.10026
H	2.86566	0.68691	-0.13781
H	1.22718	3.56076	-0.84750
H	1.21883	3.42058	0.89485
H	0.84008	0.93609	0.71572
H	0.86826	1.06031	-1.04927
H	-1.13202	2.53399	-0.96445
H	-1.13860	2.40703	0.80983
H	-3.41116	1.75352	0.25790
H	0.02353	-1.33914	2.16440
H	5.36150	1.73015	0.78548

H	5.35791	1.74464	-0.97006
H	-3.71603	-1.11692	-0.32806
H	-4.08446	0.13132	-1.50552
H	4.76444	-0.69605	-0.99089
H	4.77472	-0.70900	0.77128
H	7.19102	-0.05306	0.75702
H	7.17963	-0.04168	-0.99844
H	6.58662	-2.47595	-1.01013
H	6.59823	-2.48711	0.74504
H	9.01314	-1.83208	0.73230
H	9.00155	-1.82147	-1.02121
H	8.43917	-4.28349	0.72692
H	9.93422	-3.98203	-0.16374
H	8.42733	-4.27289	-1.03781
H	-5.81142	1.09379	0.06917
H	-5.43412	-0.16825	1.23641
H	-6.15266	-1.90582	-0.42102
H	-6.52373	-0.64558	-1.58548
H	-8.22996	0.30339	-0.01393
H	-7.85797	-0.95715	1.14989
H	-8.57080	-2.69349	-0.50454
H	-8.94309	-1.43415	-1.66687
H	-10.29999	-1.76040	1.06930
H	-11.02134	-2.18066	-0.48665
H	-1.62607	-1.98736	2.10678
H	-0.73580	-1.71185	0.59415
H	-2.71241	0.36542	1.23014

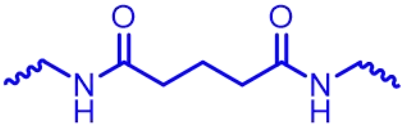

C2

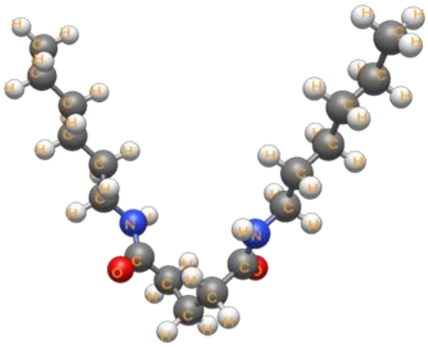

N	2.34531	-1.62635	0.29035
C	1.82195	-2.75588	-0.25321
C	1.05530	-3.64995	0.70857
C	0.00065	-4.49574	-0.00037
C	-1.05427	-3.65008	-0.70908
O	1.98121	-3.03232	-1.42942
C	-1.82064	-2.75614	0.25302
N	-2.34152	-1.62498	-0.28951
O	-1.98193	-3.03419	1.42858
C	3.16216	-0.71436	-0.49402
C	-3.15660	-0.71201	0.49554
C	3.66054	0.43636	0.36750
C	4.51824	1.42076	-0.42336

C	5.02923	2.57961	0.42894
C	5.88652	3.56912	-0.35712
C	6.39146	4.72148	0.50743
C	-3.66049	0.43532	-0.36732
C	-4.51688	1.42043	0.42407
C	-5.03242	2.57647	-0.42932
C	-5.88910	3.56625	0.35705
C	-6.39819	4.71605	-0.50848
H	2.17666	-1.42323	1.26301
H	0.59085	-3.05519	1.49975
H	1.79110	-4.30291	1.18924
H	-0.48781	-5.13386	0.73794
H	0.48926	-5.13362	-0.73878
H	-1.79023	-4.30314	-1.18936
H	-0.59009	-3.05540	-1.50045
H	-2.16868	-1.41906	-1.26085
H	2.57263	-0.33674	-1.33558
H	4.00348	-1.26724	-0.92322
H	-2.56478	-0.33070	1.33391
H	-3.99505	-1.26553	0.92935
H	4.24378	0.03620	1.20524
H	2.80259	0.96579	0.79889
H	3.93571	1.81695	-1.26320
H	5.37065	0.88893	-0.86156
H	5.61293	2.18354	1.26876
H	4.17678	3.11060	0.86983
H	5.30231	3.96415	-1.19516
H	6.73674	3.03724	-0.79731
H	5.55615	5.28165	0.93564
H	7.00173	5.41885	-0.06945
H	7.00007	4.34882	1.33542
H	-4.24614	0.03160	-1.20162
H	-2.80524	0.96519	-0.80355
H	-3.93208	1.81952	1.26097
H	-5.36702	0.88844	0.86645
H	-5.61789	2.17750	-1.26652
H	-4.18218	3.10795	-0.87390
H	-5.30324	3.96390	1.19269
H	-6.73730	3.03395	0.80063
H	-5.56491	5.27674	-0.93997
H	-7.00813	5.41356	0.06859
H	-7.00831	4.34074	-1.33415

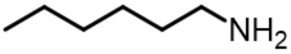

Hea

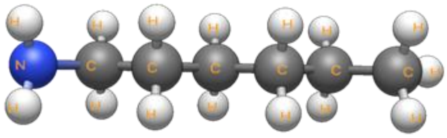

C	3.80091	-0.34607	0.00002
C	2.53065	0.50079	0.00001
C	1.26052	-0.34706	0.00001

C	-0.01914	0.48579	-0.00009
C	-1.28883	-0.36230	-0.00001
C	-2.56519	0.48021	0.00008
N	-3.82179	-0.26533	-0.00003
H	4.69868	0.27506	0.00002
H	3.83868	-0.99026	-0.88234
H	3.83868	-0.99025	0.88238
H	2.52434	1.15737	-0.87673
H	2.52433	1.15737	0.87675
H	1.26531	-1.00523	0.87741
H	1.26537	-1.00532	-0.87733
H	-0.02228	1.14386	-0.87750
H	-0.02228	1.14406	0.87718
H	-1.29242	-1.01869	0.87899
H	-1.29257	-1.01870	-0.87899
H	-2.56623	1.13754	-0.87509
H	-2.56629	1.13721	0.87549
H	-3.87728	-0.86745	0.81461
H	-3.87713	-0.86733	-0.81476

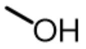

Methanol

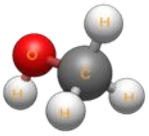

C	0.66241	-0.02054	-0.00000
O	-0.74444	0.12214	0.00000
H	-1.14478	-0.74904	-0.00000
H	1.02205	-0.54472	0.89155
H	1.02205	-0.54472	-0.89155
H	1.08170	0.98458	0.00000

H_2O

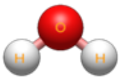

O	0.00000	0.11674	0.00000
H	0.76110	-0.46694	-0.00000
H	-0.76110	-0.46694	-0.00000

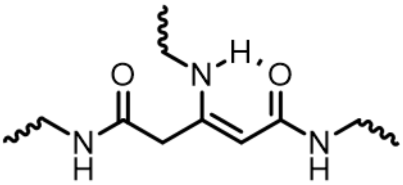

EADA (SMD, water)

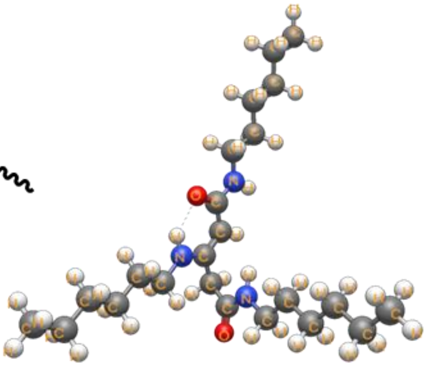

N	3.05227	-0.89140	0.79881
C	1.75175	-1.24104	0.68311
C	0.87711	-0.85503	1.77345
C	-0.46463	-1.14017	1.81213
C	-1.28088	-0.66285	2.98522
O	1.36008	-1.86941	-0.33031
C	-2.15664	0.54799	2.69143
H	-0.82109	1.23542	1.32554
O	-3.21283	0.72354	3.31044
C	4.04085	-1.20780	-0.22147
H	-2.49941	3.26874	2.34767
N	-1.13373	-1.79782	0.84630
C	-2.55527	-2.12576	0.87406
H	-2.35358	-3.63809	-0.63461
H	-0.61437	-0.37738	3.80151
H	-1.93967	-1.45119	3.35114
H	-2.51031	5.32359	0.87047
H	3.34821	-0.37654	1.61673
C	5.41947	-0.73601	0.21158
H	-3.45064	2.37952	1.15840
H	-1.67271	2.74672	-0.55120
H	-0.70140	3.61577	0.63671
C	-2.96741	-2.74948	-0.44990
H	1.30788	-0.31993	2.60952
H	-6.61527	-4.05181	-3.95907
C	-4.44371	-3.13325	-0.46159
H	-7.83456	-5.03153	-3.13696
C	-4.87981	-3.75077	-1.78726
H	-4.64107	-3.84181	0.35122
H	-5.05170	-2.24396	-0.25831
C	-6.35494	-4.14287	-1.80666
H	-4.68433	-3.04076	-2.59993
H	-4.26781	-4.63720	-1.99335
C	-6.77749	-4.75609	-3.13828
H	-6.54822	-4.85291	-0.99526
H	-6.96462	-3.25706	-1.59806
H	-6.19602	-5.65708	-3.35297
H	-2.76839	-2.82221	1.69206
H	-3.14838	-1.22202	1.04888
H	-2.76284	-2.04138	-1.26021
H	-0.58059	-2.11246	0.05688
C	-1.80690	7.50408	-2.64094
C	-2.49422	6.75068	-1.50613
C	-1.77651	5.45130	-1.15042
C	-2.45581	4.68513	-0.01893
C	-1.72977	3.39056	0.33319
C	-2.43096	2.64124	1.45395
N	-1.70759	1.41673	1.78197
H	-2.33110	8.43114	-2.88398
H	-1.76975	6.89227	-3.54665
H	-0.77889	7.76079	-2.37019
H	-3.52860	6.52410	-1.78646
H	-2.54425	7.38815	-0.61676
H	-0.74153	5.67664	-0.86610
H	-1.72387	4.81255	-2.04034

H	-3.48874	4.45528	-0.30514
H	4.05108	-2.28819	-0.39438
H	3.76017	-0.73152	-1.16644
C	6.48215	-1.04574	-0.83819
H	5.39050	0.34336	0.39807
H	5.68522	-1.21976	1.15802
C	7.87635	-0.59086	-0.41615
H	6.49628	-2.12449	-1.03290
H	6.21197	-0.55915	-1.78263
C	8.94568	-0.89202	-1.46306
H	7.86114	0.48731	-0.21560
H	8.14719	-1.08011	0.52732
C	10.33432	-0.43595	-1.02551
H	8.95722	-1.96862	-1.66487
H	8.67507	-0.40082	-2.40399
H	10.63358	-0.93595	-0.10005
H	11.08814	-0.65670	-1.78467
H	10.34900	0.64198	-0.84142

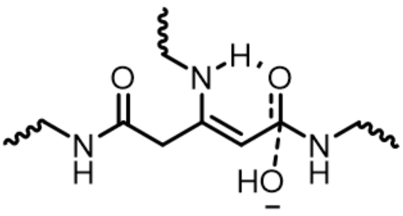

TSH1 (SMD, water)

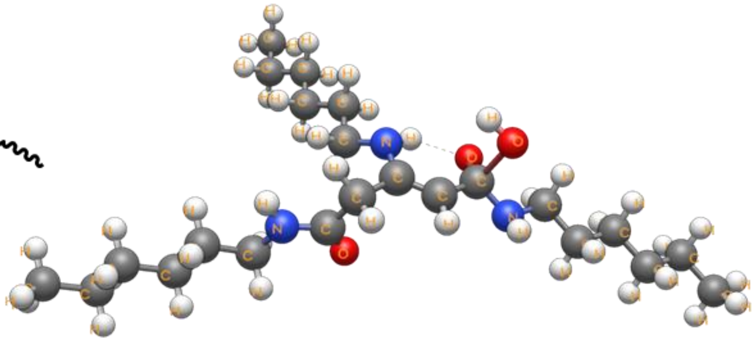

N	3.93908	-0.60845	0.39443
C	2.91159	0.22592	0.89370
C	1.72802	-0.56353	1.34189
C	0.45739	-0.11141	1.39440
C	-0.65249	-1.04774	1.80082
O	2.76636	1.37062	0.35509
C	-1.48410	-1.51392	0.61469
N	-2.79236	-1.68238	0.83470
O	-0.96630	-1.76984	-0.47912
C	5.19874	0.04224	0.04129
C	-3.70026	-2.14056	-0.21157
N	0.09031	1.20357	1.14676
C	-1.22259	1.52048	0.58472
H	1.91735	-1.58470	1.65218
H	-1.29631	-0.58958	2.55516
H	-0.21547	-1.94905	2.23688
O	3.63480	0.59038	2.62955
H	2.89055	1.04141	3.04178
C	6.26111	-0.98986	-0.30460
H	-5.34971	7.03195	-1.66100
H	-4.29867	7.30164	-0.26620
C	-5.12311	-2.22564	0.31454
H	0.85342	1.71118	0.70565

H	12.02678	-1.27064	-1.99525
H	11.25448	-2.43089	-0.90902
C	-1.36506	3.02172	0.38821
H	4.09990	-1.38403	1.02957
H	-3.17980	-1.39922	1.72538
H	5.02727	0.69225	-0.81954
H	5.55955	0.67774	0.85986
C	7.59513	-0.34238	-0.66544
H	6.40180	-1.66343	0.54957
H	5.91308	-1.60484	-1.14182
C	8.67286	-1.36139	-1.02501
H	7.44876	0.34219	-1.50948
H	7.94178	0.26872	0.17631
C	10.00992	-0.71728	-1.38256
H	8.81764	-2.04915	-0.18290
H	8.32883	-1.97094	-1.86947
C	11.07640	-1.74691	-1.74360
H	9.86325	-0.02827	-2.22146
H	10.35337	-0.11087	-0.53743
H	10.76305	-2.34556	-2.60352
H	-1.38022	1.01568	-0.37881
H	-2.00192	1.17426	1.26844
C	-2.71287	3.39233	-0.22364
H	-1.24591	3.52473	1.35378
H	-0.55662	3.37733	-0.26134
C	-2.88084	4.89640	-0.41993
H	-2.82486	2.88666	-1.18995
H	-3.51814	3.01912	0.42048
C	-4.22754	5.27264	-1.03248
H	-2.76961	5.40339	0.54623
H	-2.07475	5.27033	-1.06283
C	-4.38155	6.77855	-1.22305
H	-4.33733	4.76533	-1.99711
H	-5.03142	4.89782	-0.38958
H	-3.60200	7.16805	-1.88382
H	-3.64851	-1.44905	-1.05839
H	-3.36680	-3.11906	-0.56825
C	-6.09439	-2.69737	-0.76324
H	-5.15387	-2.91443	1.16580
H	-5.43168	-1.24112	0.68344
C	-7.53135	-2.79576	-0.25925
H	-6.05455	-2.00762	-1.61431
H	-5.77311	-3.67665	-1.13676
C	-8.50864	-3.26767	-1.33274
H	-7.57052	-3.48504	0.59290
H	-7.85242	-1.81654	0.11606
C	-9.94077	-3.36421	-0.81566
H	-8.46857	-2.57792	-2.18275
H	-8.18494	-4.24455	-1.70800
H	-10.29099	-2.39192	-0.45784
H	-10.62786	-3.70199	-1.59483
H	-10.00603	-4.06903	0.01799

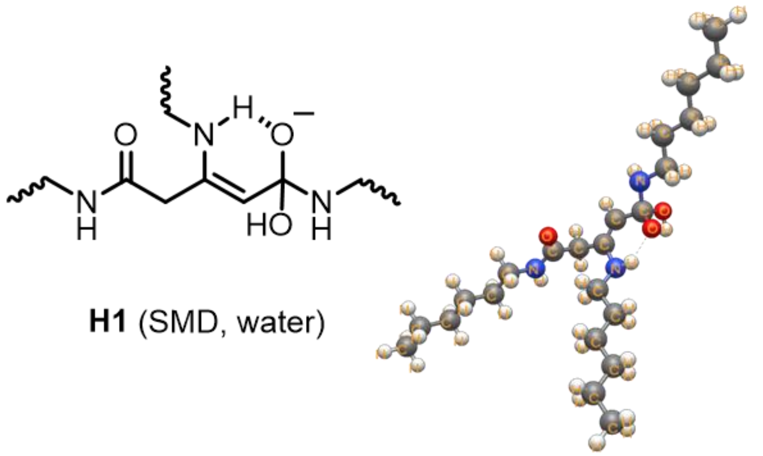

H1 (SMD, water)

N	3.92648	-0.57474	0.52598
C	2.95538	0.31280	1.18509
C	1.73222	-0.53624	1.51055
C	0.45460	-0.11853	1.55111
C	-0.65130	-1.08265	1.90321
O	2.72170	1.43655	0.49943
C	-1.45385	-1.53936	0.69437
N	-2.77121	-1.68677	0.87602
O	-0.90866	-1.81047	-0.38264
C	5.21797	0.05159	0.23212
C	-3.65875	-2.12386	-0.19608
N	0.06956	1.21257	1.35204
C	-1.19546	1.49170	0.66983
H	1.93156	-1.56687	1.78992
H	-1.31865	-0.65060	2.65317
H	-0.21144	-1.98787	2.32876
O	3.57536	0.64375	2.50917
H	2.98094	1.28756	2.91214
C	6.19965	-0.96588	-0.33171
H	-5.28109	6.88609	-1.91735
H	-4.32484	7.20024	-0.46487
C	-5.09370	-2.19905	0.29816
H	0.85481	1.71116	0.93166
H	11.87914	-1.32098	-2.28415
H	11.06157	-2.59460	-1.37195
C	-1.36612	2.98803	0.45906
H	4.09339	-1.36273	1.14909
H	-3.18043	-1.37916	1.74861
H	5.05844	0.84560	-0.50088
H	5.66162	0.51945	1.12089
C	7.56453	-0.35204	-0.63222
H	6.32099	-1.78669	0.38612
H	5.78565	-1.40321	-1.24698
C	8.55773	-1.36002	-1.20431
H	7.44123	0.47591	-1.34079
H	7.97879	0.08168	0.28573
C	9.92476	-0.75097	-1.50564
H	8.68066	-2.18925	-0.49682
H	8.14587	-1.79380	-2.12372
C	10.90609	-1.77130	-2.07488

H	9.80107	0.07633	-2.21295
H	10.33478	-0.31724	-0.58707
H	10.52593	-2.19738	-3.00761
H	-1.25126	0.98515	-0.30477
H	-2.02410	1.12018	1.27922
C	-2.67269	3.32199	-0.25505
H	-1.33568	3.49521	1.42949
H	-0.52010	3.36622	-0.12701
C	-2.85825	4.81950	-0.48339
H	-2.70305	2.80287	-1.22032
H	-3.51493	2.93831	0.33333
C	-4.16973	5.15954	-1.18676
H	-2.81980	5.34159	0.48043
H	-2.01997	5.20176	-1.07845
C	-4.33811	6.65868	-1.41493
H	-4.20912	4.63340	-2.14677
H	-5.00601	4.78086	-0.58904
H	-3.52554	7.05093	-2.03290
H	-3.58097	-1.42472	-1.03490
H	-3.32739	-3.10229	-0.55460
C	-6.04852	-2.64927	-0.80308
H	-5.14943	-2.89586	1.14160
H	-5.39928	-1.21498	0.67097
C	-7.49533	-2.73846	-0.32612
H	-5.98598	-1.95085	-1.64566
H	-5.73006	-3.62775	-1.18107
C	-8.45841	-3.18922	-1.42126
H	-7.55647	-3.43619	0.51784
H	-7.81326	-1.76004	0.05396
C	-9.89999	-3.27728	-0.92948
H	-8.39719	-2.49077	-2.26285
H	-8.13773	-4.16516	-1.80151
H	-10.24676	-2.30556	-0.56675
H	-10.57706	-3.59965	-1.72380
H	-9.98619	-3.99053	-0.10494

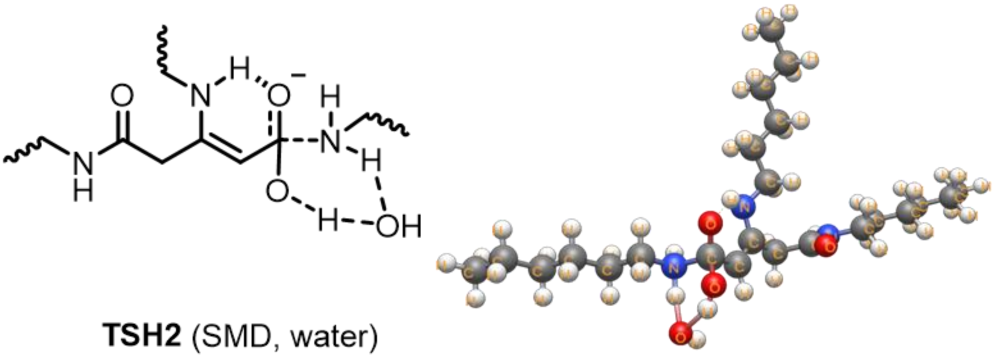

TSH2 (SMD, water)

N	-3.70792	-1.13308	0.16952
C	-2.57761	-1.13005	1.26088
C	-1.36320	-1.71053	0.55223
C	-0.34022	-0.99573	0.04302
C	0.80710	-1.70204	-0.63473
O	-2.43801	0.04600	1.82447

C	2.06485	-1.73991	0.22038
N	3.22883	-1.64899	-0.43202
O	2.01156	-1.89832	1.44601
C	-4.96408	-0.47676	0.58084
C	4.51243	-1.69042	0.26051
N	-0.29671	0.39428	0.04167
C	0.98413	1.09912	0.10000
H	-1.35379	-2.78667	0.41460
H	1.02523	-1.24641	-1.60364
H	0.52873	-2.74388	-0.80982
H	3.88426	7.73347	0.34116
H	2.42640	7.50246	-0.63057
C	-6.00251	-0.54504	-0.52955
H	-3.46034	-4.09788	-0.35381
H	-3.35846	-2.88468	1.69755
C	5.65686	-1.55705	-0.73012
H	-0.98101	0.75838	0.70252
H	11.66320	-1.36358	-0.80083
H	10.68277	-2.17318	-2.02798
C	0.75725	2.60291	0.10856
H	-3.33472	-0.67405	-0.66137
H	3.21813	-1.45927	-1.42585
H	-3.90989	-2.24458	-0.01366
O	-4.13449	-3.62513	0.14411
O	-3.06142	-2.10354	2.21000
H	4.54907	-0.88008	0.99532
H	4.59076	-2.63309	0.80921
C	7.01353	-1.58279	-0.03291
H	5.60127	-2.37272	-1.45922
H	5.54612	-0.61886	-1.28520
C	8.17896	-1.45554	-1.00979
H	7.06120	-0.76681	0.69751
H	7.11352	-2.51699	0.53186
C	9.54070	-1.47449	-0.32028
H	8.13208	-2.27344	-1.73892
H	8.07540	-0.52363	-1.57870
C	10.69618	-1.34950	-1.30871
H	9.58624	-0.65625	0.40651
H	9.64144	-2.40443	0.24976
H	10.62632	-0.41454	-1.87174
H	1.55945	0.81830	0.99339
H	1.58367	0.83304	-0.77434
C	2.06727	3.38046	0.19488
H	0.21534	2.89086	-0.79868
H	0.11857	2.86382	0.96069
C	1.85878	4.89232	0.19639
H	2.60469	3.08844	1.10478
H	2.70904	3.10433	-0.65044
C	3.16561	5.67682	0.28025
H	1.31986	5.18530	-0.71283
H	1.21709	5.16866	1.04185
C	2.94088	7.18594	0.28112
H	3.70303	5.38313	1.18846
H	3.80555	5.39952	-0.56451
H	2.32390	7.48607	1.13274

H	-5.32447	-0.99285	1.47333
H	-4.75441	0.56049	0.84743
C	-7.30560	0.13812	-0.12382
H	-5.60042	-0.06549	-1.42948
H	-6.19740	-1.59294	-0.78191
C	-8.37150	0.07242	-1.21395
H	-7.69123	-0.33119	0.78887
H	-7.10304	1.18673	0.12367
C	-9.67496	0.76059	-0.81685
H	-7.98334	0.53488	-2.12961
H	-8.57693	-0.97707	-1.45709
C	-10.73405	0.68049	-1.91221
H	-10.05789	0.30134	0.10096
H	-9.46878	1.80966	-0.57847
H	-10.97222	-0.36083	-2.14653
H	-11.66001	1.17730	-1.61370
H	-10.37935	1.15583	-2.83106

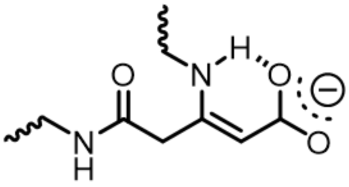
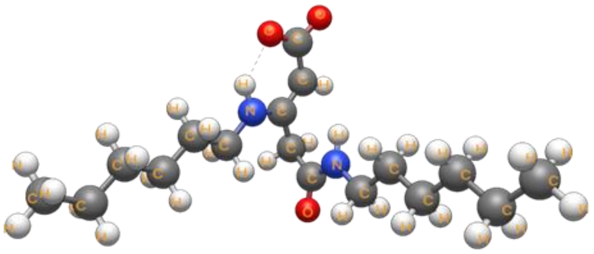

H2 (SMD, water)

O	-0.30310	4.75591	-2.18779
C	0.35357	3.78170	-1.70459
C	0.19291	3.49498	-0.26897
C	0.81002	2.47979	0.41011
C	0.52701	2.29501	1.87954
O	1.13342	3.05602	-2.40244
C	-0.38894	1.12363	2.20466
H	-1.35039	1.33209	0.42986
O	-0.30053	0.53684	3.29093
H	-3.70589	-2.46612	0.71273
H	-2.88043	-0.02227	2.40099
N	1.64342	1.57517	-0.15433
C	2.38769	0.54946	0.56420
H	3.79365	0.29403	-1.03558
H	0.03904	3.18984	2.27133
H	1.44835	2.14433	2.44395
H	-4.82475	-1.28947	1.38883
H	-5.64724	-0.75107	-0.91264
H	-4.53061	-1.92949	-1.58714
H	-1.75312	-1.19373	1.71833
H	-2.57037	-0.64699	-0.57775
H	-3.69282	0.53179	0.10045
C	3.13632	-0.33298	-0.42252
H	-0.46587	4.14541	0.29175
H	5.61404	-4.76474	-1.66380
C	3.96221	-1.40499	0.28151

H	6.90243	-5.01628	-0.48078
C	4.72030	-2.29882	-0.69597
H	4.67555	-0.92533	0.96183
H	3.30288	-2.02318	0.90209
C	5.55596	-3.37099	-0.00158
H	4.00685	-2.78067	-1.37538
H	5.37531	-1.67895	-1.32019
C	6.30940	-4.25424	-0.99158
H	6.26692	-2.88799	0.67752
H	4.90086	-3.98987	0.62112
H	6.98827	-3.65662	-1.60643
H	3.10133	1.00530	1.26040
H	1.70461	-0.06919	1.15638
H	2.41740	-0.80502	-1.10086
H	1.84548	1.73166	-1.13649
C	-7.08600	-2.96526	-1.71777
C	-6.17653	-2.79770	-0.50426
C	-5.13619	-1.69839	-0.70251
C	-4.21954	-1.52034	0.50446
C	-3.18481	-0.41818	0.29982
C	-2.28546	-0.26037	1.51428
N	-1.31046	0.80221	1.29323
H	-7.82496	-3.75433	-1.56084
H	-6.50369	-3.22291	-2.60689
H	-7.62512	-2.03778	-1.93018
H	-5.66449	-3.74282	-0.29341
H	-6.77985	-2.56536	0.38000

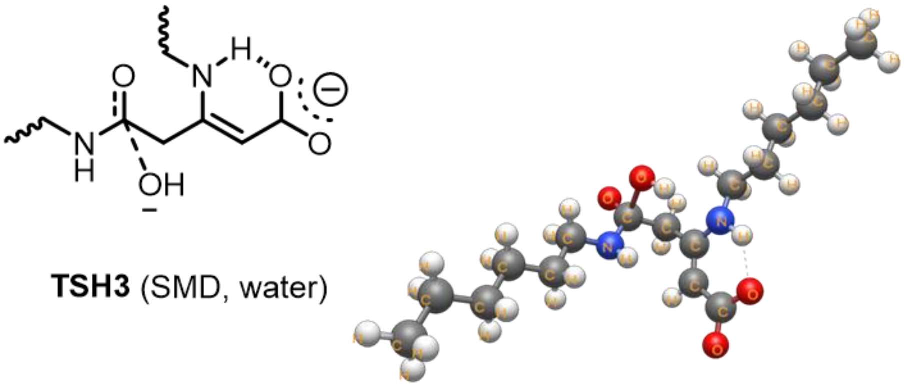

TSH3 (SMD, water)

O	-0.57312	4.42200	-0.88367
C	0.21628	3.44082	-0.71835
C	0.00256	2.59124	0.46927
C	0.74464	1.50146	0.82835
C	0.37844	0.74165	2.08200
O	1.16649	3.17384	-1.52233
C	-0.72161	-0.33204	1.96768
H	-1.63663	0.58825	0.43138
O	-0.87149	-1.09711	2.96616
O	0.12948	-1.51738	0.64646
H	-3.37871	-1.10197	2.07933
N	1.78244	1.02628	0.07378
C	2.90417	0.26317	0.62019

H	9.21296	-2.74960	-1.27349
H	-0.00174	1.45601	2.81687
H	1.24642	0.25088	2.51864
N	-1.87049	0.10930	1.29511
H	2.54199	-0.67207	1.04970
C	-2.94132	-0.86588	1.10649
H	-2.54617	-1.80207	0.69211
H	7.88267	-2.81972	-2.43461
C	-5.14527	-1.31202	-0.05007
C	3.90180	-0.04492	-0.48603
H	-0.82003	2.87142	1.11497
H	2.01764	1.65467	-0.69055
H	3.40425	0.82510	1.41979
C	-4.01514	-0.31314	0.18186
C	5.11584	-0.80768	0.03529
H	0.51863	-0.94217	-0.02192
H	4.22887	0.89283	-0.95046
H	3.40257	-0.63206	-1.26487
C	6.12522	-1.13212	-1.06251
H	5.60841	-0.21677	0.81646
H	4.78330	-1.73892	0.50887
C	7.34549	-1.89141	-0.54816
H	5.63390	-1.72504	-1.84348
H	6.45449	-0.20068	-1.53895
C	8.34577	-2.20668	-1.65632
H	7.83436	-1.29848	0.23250
H	7.01551	-2.82156	-0.07294
H	8.70620	-1.28767	-2.12703
H	-4.42276	0.61055	0.60732
H	-3.55997	-0.04779	-0.78012
C	-6.23190	-0.77329	-0.97651
H	-4.73268	-2.23478	-0.47484
H	-5.59285	-1.58368	0.91339
C	-7.36221	-1.77056	-1.21771
H	-6.64818	0.14781	-0.55074
H	-5.78348	-0.49677	-1.93853
C	-8.44007	-1.21785	-2.14546
H	-6.94498	-2.68977	-1.64307
H	-7.80888	-2.04651	-0.25629
H	-8.01777	-0.95923	-3.12056
H	-9.24122	-1.94240	-2.30852
H	-8.88696	-0.31227	-1.72562

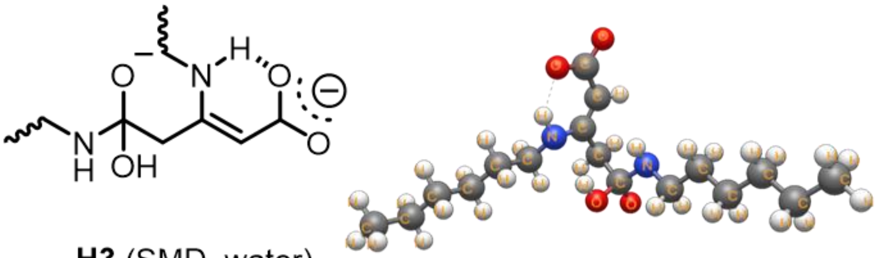

H3 (SMD, water)

O	-0.33670	4.60876	-1.34501
C	0.34606	3.56773	-1.10080
C	0.10777	2.89459	0.19487
C	0.75234	1.79173	0.66979
C	0.36349	1.21928	2.00893
O	1.20852	3.09801	-1.90929
C	-0.57635	-0.02904	1.96994
H	-1.51433	0.68188	0.30341
O	-0.84337	-0.48317	3.19349
H	-4.42943	-2.28975	-0.52274
H	-3.10162	-1.06972	2.02390
N	1.68727	1.09516	-0.06802
C	2.85056	0.45903	0.55905
H	3.98232	0.51021	-1.26653
H	-0.16144	1.98476	2.57993
H	1.24630	0.91953	2.57700
H	-5.27650	-1.81796	0.94340
H	-6.63850	-0.18089	-0.36967
H	-5.79171	-0.65154	-1.83614
H	-2.27493	-1.63370	0.57520
H	-3.56291	0.03861	-0.78125
H	-4.42881	0.52741	0.67197
C	3.69775	-0.22392	-0.50346
H	-0.65837	3.33416	0.82153
H	7.33520	-3.68692	-1.95221
C	4.95260	-0.86236	0.08416
H	8.80447	-3.35201	-1.02927
C	5.80633	-1.56252	-0.96953
H	5.55282	-0.09266	0.58343
H	4.66432	-1.58544	0.85637
C	7.06545	-2.20341	-0.39154
H	5.20637	-2.33278	-1.46918
H	6.09213	-0.83946	-1.74318
C	7.90806	-2.89757	-1.45742
H	7.66334	-1.43342	0.10805
H	6.77854	-2.92559	0.38052
H	8.22618	-2.18637	-2.22491
H	3.45582	1.20265	1.09446
H	2.51920	-0.28143	1.28731
H	3.09323	-0.98882	-1.00382
H	1.91586	1.59449	-0.92503
C	-8.34260	-1.70685	-1.91300
C	-7.14210	-2.14893	-1.08149
C	-6.13583	-1.02194	-0.86269
C	-4.93022	-1.44822	-0.02937

C	-3.92693	-0.31890	0.19002
C	-2.74263	-0.75547	1.04106
N	-1.79058	0.34089	1.22274
H	-9.05293	-2.52352	-2.06102
H	-8.02428	-1.35611	-2.89877
H	-8.87299	-0.88555	-1.42290
H	-6.63978	-2.98789	-1.57530
H	-7.48341	-2.51888	-0.10850
O	0.13675	-1.09957	1.24169
H	0.45488	-0.73718	0.40274

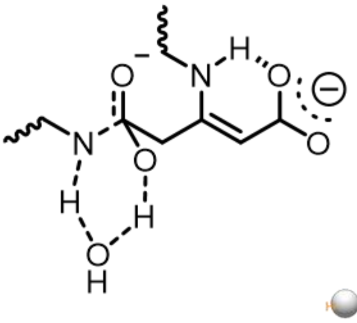

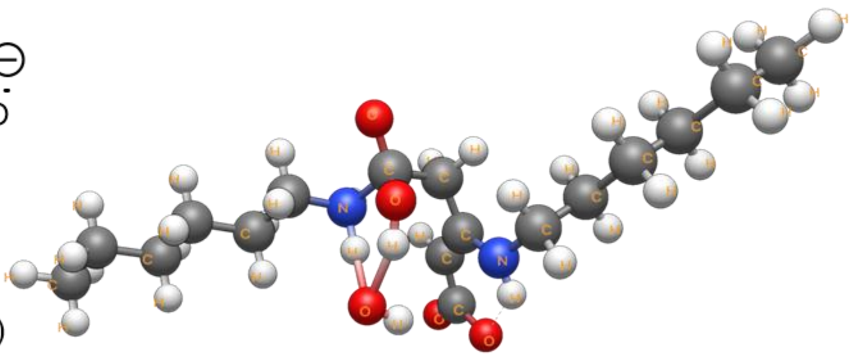

TSH4 (SMD, water)

O	0.35375	4.30558	-1.05786
C	-0.34430	3.45197	-0.42358
C	-0.35476	2.07050	-0.92750
C	-1.04102	1.01684	-0.38306
C	-0.84724	-0.35524	-0.98097
O	-1.01686	3.74898	0.61778
C	0.23736	-1.26513	-0.32742
H	1.55400	0.27917	0.37566
O	0.31032	-2.43607	-0.90074
H	5.23987	-1.77208	0.93135
H	2.81802	-2.08899	-0.95453
N	-1.83452	1.12823	0.71653
C	-2.87892	0.18176	1.08576
H	-3.83587	0.09681	-0.84245
H	-0.56742	-0.24479	-2.02986
H	-1.76634	-0.94132	-0.94518
H	5.36211	-2.07322	-0.79710
H	6.65262	0.05454	-1.07393
H	6.52907	0.35286	0.65391
H	2.69129	-1.75620	0.77910
H	3.99392	0.35335	0.47089
H	4.10513	0.03737	-1.26015
C	-4.12276	0.26308	0.20251
H	0.26970	1.86871	-1.78944
H	-9.20415	-0.65211	-0.64611
C	-5.17894	-0.75691	0.61709
H	-9.49776	-2.37461	-0.38280
C	-6.43780	-0.69663	-0.24373
H	-4.74970	-1.76459	0.56047
H	-5.44970	-0.59147	1.66695

C	-7.49795	-1.71211	0.17517
H	-6.86448	0.31246	-0.19248
H	-6.16822	-0.86828	-1.29293
C	-8.74804	-1.64480	-0.69697
H	-7.06906	-2.71900	0.12814
H	-7.76834	-1.53678	1.22213
H	-8.50279	-1.84524	-1.74372
H	-2.47475	-0.83143	1.07515
H	-3.15319	0.39839	2.12092
H	-4.53792	1.27531	0.26197
H	-1.94530	2.08619	1.03314
C	9.11535	-0.41674	0.08513
C	7.84906	-1.26647	0.13414
C	6.58520	-0.44277	-0.09875
C	5.30896	-1.27772	-0.04468
C	4.05171	-0.44443	-0.27712
C	2.79350	-1.29665	-0.20644
N	1.58952	-0.46819	-0.42844
H	10.01104	-1.01910	0.25311
H	9.08808	0.36477	0.84963
H	9.21755	0.07265	-0.88755
H	7.77738	-1.76636	1.10618
H	7.90618	-2.05829	-0.62062
O	0.04024	-1.37276	1.08328
H	0.32586	-0.52353	1.50444
H	1.64334	-0.01658	-1.34210
O	1.35492	0.89735	1.76383
H	0.80962	1.67522	1.60340

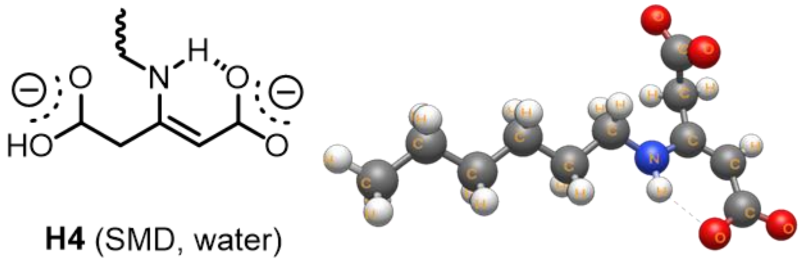

H4 (SMD, water)

O	4.55376	-2.55144	0.15166
C	3.39202	-2.03578	0.09913
C	3.28755	-0.63263	-0.32955
C	2.12767	0.08293	-0.45651
C	2.18345	1.52307	-0.88600
O	2.33678	-2.68395	0.40026
C	1.77846	2.52418	0.21122
H	-1.56630	-1.03696	-1.18077
O	1.99220	2.20626	1.40718
C	-6.61583	-0.83880	0.01981
H	0.89992	-1.37890	0.13818
N	0.89077	-0.44066	-0.25098
C	-0.33079	0.33652	-0.08603
H	-1.46029	-1.37661	0.54536
H	1.55259	1.68878	-1.76134
H	3.20898	1.77151	-1.16845

H	-5.46136	0.89338	-0.54644
H	-0.38404	1.10554	-0.86028
H	-5.36799	0.49982	1.16240
H	-6.57383	-1.66725	0.73252
O	1.29600	3.61911	-0.17662
H	-0.34099	0.84872	0.88457
C	-1.54907	-0.56911	-0.19069
H	4.21488	-0.12752	-0.56688
H	-6.67081	-1.26669	-0.98516
C	-2.84955	0.19371	0.04290
H	-7.54151	-0.28762	0.20056
C	-4.08477	-0.69351	-0.08462
H	-2.83048	0.64743	1.04068
H	-2.92068	1.01890	-0.67566
C	-5.39020	0.05907	0.15983
H	-4.10809	-1.14065	-1.08583
H	-4.00896	-1.52502	0.62659

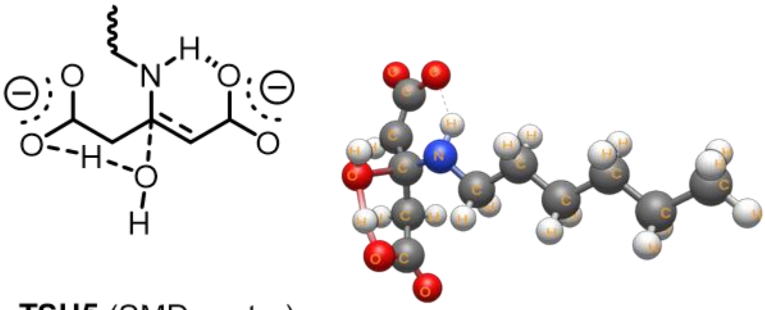

TSH5 (SMD, water)

O	3.90638	-3.11262	-0.62682
C	2.91796	-2.35823	-0.24291
C	3.04572	-0.94939	-0.35949
C	2.09771	0.07157	0.06341
C	2.15585	1.32006	-0.83077
O	1.83703	-2.92281	0.21370
C	1.49715	2.59918	-0.34504
H	-1.53573	-1.49245	-0.21853
O	0.95682	3.37760	-1.11787
C	-6.69577	-0.81073	0.04524
H	0.76202	-1.39490	0.29220
N	0.80652	-0.38357	0.40908
C	-0.39762	0.31753	-0.02561
H	-1.66805	-0.72226	1.36049
H	1.73708	1.07827	-1.80691
H	3.21209	1.56673	-0.97740
H	-5.41826	0.22902	-1.34807
H	-0.38248	0.53560	-1.10284
H	-5.53223	0.99659	0.22716
H	-6.76702	-1.00580	1.11899
O	1.58115	2.85681	0.94013
H	-0.48858	1.27382	0.49943
C	-1.62876	-0.52238	0.28420
H	3.99822	-0.57720	-0.72011

H	-6.65418	-1.77632	-0.46651
C	-2.91891	0.16255	-0.15694
H	-7.61336	-0.30578	-0.26536
C	-4.16470	-0.66175	0.15593
H	-2.99798	1.13815	0.33740
H	-2.87686	0.35980	-1.23458
C	-5.45918	0.02457	-0.27266
H	-4.08998	-1.63579	-0.34271
H	-4.20408	-0.86511	1.23300
O	2.69229	0.78706	1.47945
H	2.06419	2.00702	1.37487
H	2.32345	0.22956	2.17498

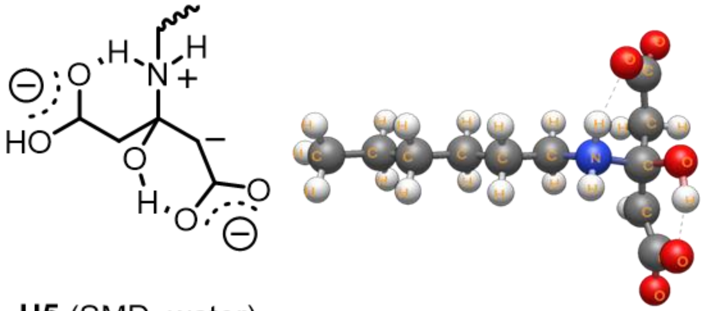

H5 (SMD, water)

O	-2.79330	-3.51333	0.91577
C	-2.68751	-2.41242	0.24562
C	-2.26297	-1.20991	0.83383
C	-2.10426	0.02404	0.03004
C	-2.23249	1.29989	0.85338
O	-3.01148	-2.41986	-1.03989
C	-1.96520	2.64172	0.15861
H	1.75131	-1.10442	-0.98301
O	-2.34687	3.66356	0.78267
C	6.84298	-0.39903	-0.18234
H	-0.63141	-0.79463	-1.22214
N	-0.67919	0.05652	-0.65650
C	0.49512	0.12936	0.24405
H	1.87276	0.62218	-1.32832
H	-1.57521	1.24555	1.72661
H	-3.25301	1.32302	1.23936
H	5.43907	-0.87629	1.38481
H	0.35217	-0.61803	1.02632
H	5.55844	0.83958	1.03027
H	6.97070	0.32908	-0.98828
O	-1.37750	2.66150	-0.95696
H	0.49650	1.12225	0.69710
C	1.78657	-0.11211	-0.52000
H	-1.99659	-1.18264	1.88227
H	6.85146	-1.39666	-0.63028
C	3.00202	-0.01283	0.39765
H	7.70824	-0.32148	0.47993
C	4.31514	-0.25571	-0.34071
H	3.02416	0.97963	0.86239

H	2.90185	-0.74018	1.21150
C	5.53743	-0.15321	0.56792
H	4.29200	-1.24910	-0.80470
H	4.41088	0.46908	-1.15813
O	-2.96789	0.07656	-1.07790
H	-0.69097	0.88428	-1.26744
H	-3.11267	-0.91309	-1.28051

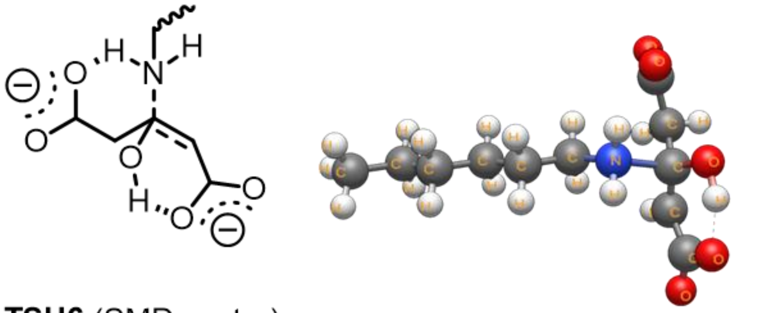

TSH6 (SMD, water)

O	-2.82870	-3.45256	0.93688
C	-2.86259	-2.34906	0.29328
C	-2.38591	-1.13184	0.86898
C	-2.32900	0.07216	0.11102
C	-2.19967	1.36388	0.88569
O	-3.36017	-2.31400	-0.91918
C	-1.74685	2.65480	0.18776
C	1.83309	-0.18238	-0.63446
O	-1.53960	3.62633	0.96404
H	-1.94190	-1.15088	1.85515
H	-0.67979	-0.84145	-1.26690
N	-0.65258	0.06479	-0.80238
C	0.52582	0.18363	0.05742
C	3.02954	-0.03438	0.30169
H	-1.54101	1.19901	1.74106
H	-3.19075	1.57559	1.30203
H	-3.36014	-0.83149	-1.17433
H	0.36388	-0.46741	0.92348
H	-0.69470	0.81315	-1.49186
O	-3.14236	0.15276	-0.98632
O	-1.62318	2.69954	-1.06133
H	0.57172	1.21539	0.41761
C	4.35432	-0.39330	-0.36538
H	3.07637	0.99845	0.66682
H	2.88175	-0.67156	1.18165
C	5.55322	-0.23616	0.56659
H	4.31052	-1.42788	-0.72672
H	4.49888	0.24012	-1.24899
C	6.87137	-0.59753	-0.11137
H	5.59457	0.79772	0.92621
H	5.40661	-0.86790	1.44939
H	7.04765	0.03976	-0.98247
H	7.71877	-0.47874	0.56773
H	6.85967	-1.63561	-0.45523

H	1.76941	-1.21501	-0.99610
H	1.96865	0.45962	-1.51185

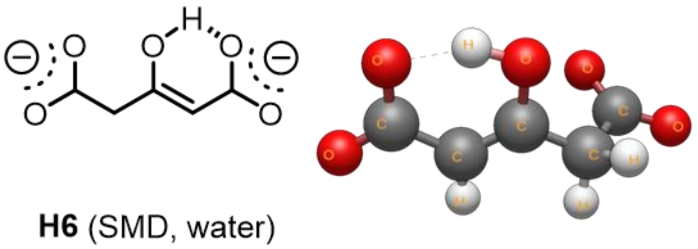

H6 (SMD, water)

O	-3.30631	-1.06217	-0.33827
C	-2.38432	-0.24050	-0.11228
C	-1.04560	-0.74454	0.26466
C	-0.01046	0.08769	0.51199
C	1.36411	-0.36414	0.87161
O	-2.53511	1.03122	-0.19144
C	2.40317	-0.14375	-0.24421
O	-0.13956	1.42781	0.44353
O	3.60206	-0.34370	0.07906
H	-0.89636	-1.81343	0.33572
H	1.70613	0.17377	1.75983
H	-1.10371	1.56725	0.17678
H	1.36043	-1.43003	1.10840
O	2.00043	0.18857	-1.38430

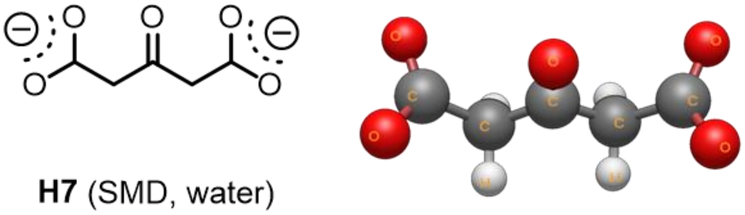

H7 (SMD, water)

O	-2.93945	-1.12425	-0.33366
C	-2.50498	-0.00304	0.03256
C	-1.27911	0.00355	0.94449
C	-0.00022	-0.01031	0.13924
C	1.27905	-0.12359	0.93580
O	-2.97433	1.11120	-0.30980
C	2.50506	0.00044	0.03252
O	2.90961	1.16243	-0.22407
O	3.00488	-1.06337	-0.41188
H	1.27078	0.65755	1.70020
H	1.25885	-1.09128	1.44448
O	-0.00054	0.06373	-1.07404
H	-1.27162	-0.86682	1.60534
H	-1.25810	0.90028	1.56998

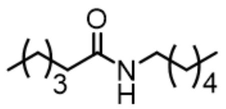

N-hexylhexanamide
(SMD, water)

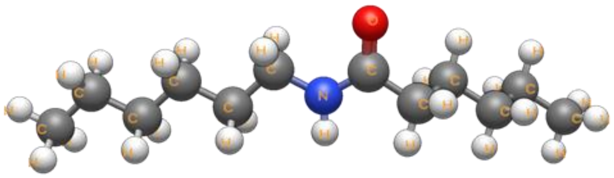

N	0.04077	0.35430	0.41605
C	1.17731	1.04104	0.24870
C	2.43118	0.44608	0.84031
C	3.47192	0.15261	-0.24383
C	4.75725	-0.42337	0.34191
O	1.20300	2.11473	-0.37346
C	5.80831	-0.73006	-0.72162
C	7.08923	-1.30357	-0.12326
H	-7.14032	-2.13959	-0.37265
C	-1.23885	0.81388	-0.11200
H	-7.40564	-1.18684	1.09106
C	-2.35119	-0.14693	0.27367
C	-3.70382	0.30785	-0.26563
C	-4.83964	-0.63226	0.12786
C	-6.19747	-0.18528	-0.40743
C	-7.32441	-1.12877	0.00200
H	0.06970	-0.51727	0.92800
H	2.19634	-0.46519	1.39531
H	2.83408	1.17775	1.54731
H	3.69686	1.07521	-0.78737
H	3.04948	-0.55270	-0.96821
H	4.52550	-1.34050	0.89646
H	5.17225	0.28559	1.06842
H	-1.16923	0.89848	-1.20054
H	-1.44727	1.81350	0.27999
H	6.03723	0.18672	-1.27545
H	5.39008	-1.43710	-1.44612
H	-8.28899	-0.79683	-0.38879
H	-2.39778	-0.22496	1.36545
H	-2.11740	-1.14535	-0.11213
H	-3.65349	0.38011	-1.35827
H	-3.92196	1.31556	0.10673
H	-4.88842	-0.70329	1.22119
H	-4.62032	-1.64148	-0.24119
H	-6.14907	-0.11978	-1.49976
H	-6.41158	0.82533	-0.04314
H	6.88424	-2.23335	0.41476
H	7.83167	-1.51915	-0.89494
H	7.53577	-0.60023	0.58516

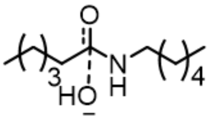

TSI1 (SMD, water)

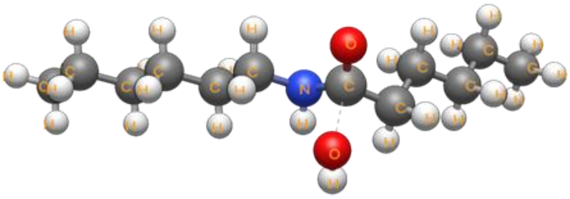

N	-0.11826	0.35139	-0.09178
C	-1.15491	1.24354	0.21640
C	-2.47177	0.90153	-0.46291
C	-3.21314	-0.21698	0.27451
C	-4.58002	-0.50708	-0.34017
O	-1.17011	1.77476	1.36157
C	-5.33628	-1.61701	0.38580
C	-6.70139	-1.89636	-0.23575
H	2.00846	-1.29831	0.03340
C	1.20154	0.64972	0.45762
H	3.90035	1.10198	0.18053
H	8.19473	-1.20089	0.02696
H	7.21958	-1.29333	-1.44379
H	-2.29639	0.61161	-1.50344
H	-4.45431	-0.78539	-1.39371
H	-5.18424	0.40821	-0.32950
H	1.17647	0.49939	1.53950
H	1.46362	1.70087	0.28129
C	2.25957	-0.24996	-0.16224
H	-3.08617	1.80417	-0.46445
H	-0.72848	2.40619	-1.84282
H	-4.73161	-2.53036	0.37447
H	6.99151	-2.48193	-0.15712
H	-3.34225	0.06857	1.32425
C	6.13407	-0.53596	0.27564
C	7.19792	-1.42700	-0.35855
H	-0.07621	0.16931	-1.08895
H	-5.45978	-1.33769	1.43777
H	4.49241	-1.88886	-0.05558
H	4.72104	-0.70942	-1.33884
O	-0.70406	2.76501	-0.94933
H	3.66486	-0.07958	1.46113
H	2.25043	-0.11700	-1.25101
H	6.37125	0.51602	0.08322
H	-2.61237	-1.13242	0.26517
H	6.14524	-0.66454	1.36343
C	3.65699	0.05028	0.37238
C	4.73245	-0.83631	-0.24939
H	-6.59736	-2.20238	-1.28055
H	-7.23034	-2.69118	0.29519
H	-7.32945	-1.00137	-0.21199

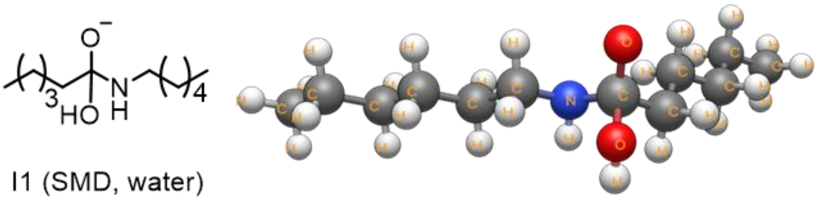

I1 (SMD, water)

N	-0.14494	0.42263	-0.16984
C	-1.16793	1.45795	0.07143
C	-2.50801	0.96621	-0.51155
C	-3.14105	-0.20841	0.22644
C	-4.53304	-0.54518	-0.30551
O	-1.22472	1.84145	1.34666
C	-5.17616	-1.73431	0.40368
C	-6.56754	-2.05305	-0.13559
H	1.96275	-1.22306	0.16028
C	1.19604	0.78256	0.29471
H	3.90952	1.12654	-0.06755
H	8.14417	-1.29085	0.12144
H	7.16271	-1.56389	-1.32247
H	-2.37215	0.70584	-1.56953
H	-4.47098	-0.75892	-1.37990
H	-5.18391	0.33167	-0.20106
H	1.19373	0.79790	1.38727
H	1.49002	1.78744	-0.03903
C	2.23211	-0.22104	-0.19200
H	-3.19189	1.82133	-0.47688
H	-0.81435	2.41223	-1.66571
H	-4.52839	-2.61089	0.29387
H	6.90581	-2.55144	0.11936
H	-3.21186	0.03060	1.29334
C	6.10314	-0.54094	0.27126
C	7.14045	-1.54174	-0.22925
H	-0.09854	0.25966	-1.17432
H	-5.23559	-1.52322	1.47698
H	4.42383	-1.88087	0.13659
H	4.68055	-0.90538	-1.30280
O	-0.81287	2.66528	-0.73454
H	3.65838	0.15638	1.37667
H	2.21471	-0.25473	-1.28860
H	6.36867	0.46630	-0.06783
H	-2.50356	-1.09392	0.14196
H	6.11435	-0.51440	1.36633
C	3.64253	0.12162	0.28065
C	4.69194	-0.87406	-0.20635
H	-6.52569	-2.28856	-1.20284
H	-7.01363	-2.90743	0.37873
H	-7.23812	-1.19830	-0.00978

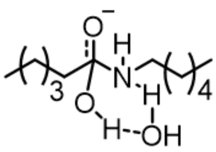

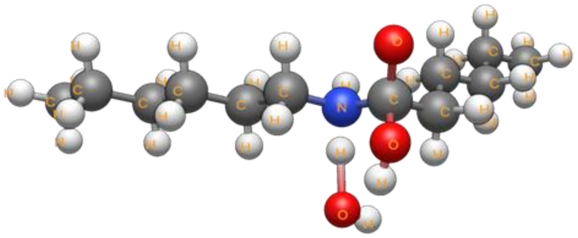

N	-0.17264	-0.61722	-0.01814
C	-1.17006	-1.48106	-0.49602
C	-2.52341	-1.00033	0.11622
C	-2.99364	0.38923	-0.30229
C	-4.41468	0.68674	0.17461
O	-1.21294	-1.51943	-1.85032
C	-4.89635	2.08233	-0.21319
C	-6.32000	2.36040	0.26003
H	1.81928	1.19443	-0.10536
C	1.19654	-0.80736	-0.57005
H	4.23477	1.99819	0.00769
H	7.98393	1.65387	-0.09064
H	7.00684	1.62666	1.38154
H	-2.46189	-1.05608	1.21067
H	-4.46348	0.57798	1.26500
H	-5.09901	-0.06103	-0.24393
H	1.15929	-0.66514	-1.64946
H	1.48921	-1.83797	-0.36390
C	2.16284	0.16999	0.07917
H	-0.71833	-1.23713	2.92539
H	-0.71018	-2.62506	0.95531
H	-4.21450	2.82811	0.20975
H	6.66900	2.81548	0.11950
H	-2.95531	0.47639	-1.39325
C	5.99248	0.80849	-0.35339
C	6.97133	1.77996	0.29930
H	-0.44171	0.35461	-0.17737
H	-4.84141	2.19108	-1.30183
H	-0.09588	-0.82688	1.08893
O	-0.01220	-1.55476	2.35432
O	-0.89717	-2.70460	-0.00455
H	3.57483	0.15085	-1.54744
H	2.16162	0.01893	1.16389
H	6.32567	-0.22107	-0.18360
H	-6.39032	2.27968	1.34844
H	-7.02071	1.64099	-0.17299
C	3.57985	0.00085	-0.46156
H	-3.25704	-1.74604	-0.20519
C	4.57093	0.96840	0.17913
H	5.98900	0.96071	-1.43815
H	3.91328	-1.02882	-0.28757
H	4.57339	0.81761	1.26541
H	-2.32830	1.16237	0.10278
H	-6.65014	3.36230	-0.02384

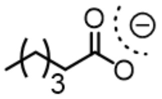

I2 (SMD, water)

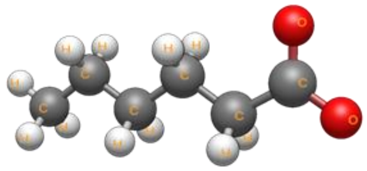

C	-3.99075	-0.23335	-0.00000
C	-2.67009	0.53199	0.00014
C	-1.46056	-0.39868	-0.00017
C	-0.12555	0.34279	0.00012
C	1.05508	-0.61872	-0.00011
H	-4.06917	-0.87152	0.88469
C	2.44768	0.01199	0.00003
O	3.42327	-0.79109	0.00018
O	2.55740	1.26517	-0.00015
H	-2.62208	1.18477	0.87846
H	1.00612	-1.27762	-0.87385
H	-1.51114	-1.05418	0.87799
H	-1.51114	-1.05352	-0.87883
H	-0.07096	0.99454	0.87843
H	-0.07088	0.99503	-0.87782
H	1.00609	-1.27812	0.87324
H	-4.84878	0.44263	-0.00329
H	-4.06615	-0.87626	-0.88153
H	-2.62214	1.18543	-0.87770

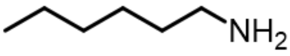

Hea (SMD, water)

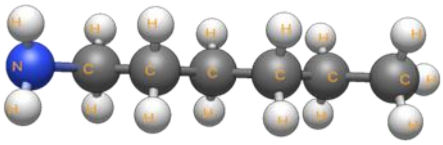

C	3.79250	-0.35079	0.00000
C	2.52605	0.50010	0.00000
C	1.25484	-0.34518	0.00000
C	-0.02207	0.49097	-0.00000
C	-1.29189	-0.35598	-0.00001
C	-2.56084	0.48864	0.00001
N	-3.81460	-0.27513	-0.00001
H	4.69316	0.26744	0.00001
H	3.82763	-0.99487	-0.88320
H	3.82762	-0.99488	0.88320
H	2.52150	1.15523	-0.87798
H	2.52150	1.15522	0.87799
H	1.25839	-1.00161	0.87878
H	1.25839	-1.00161	-0.87878
H	-0.02445	1.14712	-0.87886
H	-0.02446	1.14711	0.87886
H	-1.29929	-1.00943	0.88096
H	-1.29930	-1.00939	-0.88101
H	-2.56124	1.14198	-0.87674
H	-2.56124	1.14193	0.87680
H	-3.81378	-0.89243	0.80847
H	-3.81375	-0.89244	-0.80848

H_2O (SMD, water) 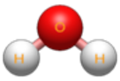

O	0.00000	0.11816	0.00000
H	0.76021	-0.47264	-0.00000
H	-0.76021	-0.47264	0.00000

OH^{-1} (SMD, water) 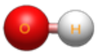

O	0.00000	0.00000	0.10678
H	0.00000	0.00000	-0.85425
